# Substitution of Dairy Products and Risk of Death and Cardiometabolic Diseases: A Systematic Review and Meta-Analysis of Prospective Studies

**DOI:** 10.1016/j.cdnut.2024.102159

**Published:** 2024-04-23

**Authors:** Eva Kiesswetter, Manuela Neuenschwander, Julia Stadelmaier, Edyta Szczerba, Lara Hofacker, Kathrin Sedlmaier, Martin Kussmann, Christine Roeger, Hans Hauner, Sabrina Schlesinger, Lukas Schwingshackl

**Affiliations:** 1 Institute for Evidence in Medicine, Medical Center and Faculty of Medicine, University of Freiburg, Freiburg, Germany; 2 German Diabetes Center, Institute for Biometrics and Epidemiology, Düsseldorf, Germany; 3 German Center for Diabetes Research (DZD), Munich-Neuherberg, Partner Düsseldorf, Germany; 4 Competence Center for Nutrition, Bavarian State Ministry for Nutrition, Agriculture and Forestry, Freising, Germany; 5 Kussmann Biotech GmbH, Nordkirchen, Germany; 6 Else Kröner Fresenius Center for Nutritional Medicine, ZIEL – Institute for Food and Health, Technical University of Munich, Freising, Germany; 7 Institute of Nutritional Medicine, School of Medicine, Technical University of Munich, Munich, Germany

**Keywords:** dairy products, substitution, cardiometabolic disease, meta-analysis, plant-based food, animal-based food

## Abstract

Substitution models in epidemiologic studies specifying both substitute and substituted food in relation to disease risk may be useful to inform dietary guidelines. A systematic review of prospective observational studies was performed to quantify the risks of all-cause mortality, cardiovascular disease, and type 2 diabetes (T2D) associated with the substitution of dairy products with other foods and between different dairy products. We systematically searched MEDLINE, Embase, and Web of Science until 28th June, 2023. We calculated summary relative risks (SRRs) and 95% confidence intervals (95% CI) in random-effects meta-analyses. We assessed the risk of bias with the Risk Of Bias In Non-randomized Studies - of Exposure (ROBINS-E) tool and certainty of evidence (CoE) using the Grading of Recommendations Assessment, Development, and Evaluations (GRADE) approach. Fifteen studies (with 34 publications) were included. There was moderate CoE that the substitution of low-fat dairy with red meat was associated with a higher risk of mortality, coronary artery disease, and T2D [SRR (95% CI): 1.11 (1.06, 1.16), 1.13 (1.08, 1.18), and 1.20 (1.16, 1.25)]. A higher risk of mortality and T2D was also observed when substituting low-fat dairy with processed meat [SRR (95% CI): 1.19 (1.11, 1.28) and 1.41 (1.33, 1.49); moderate CoE]. A lower mortality risk was associated with the substitution of dairy and yogurt with whole grains [SRR (95% CI): 0.89 (0.84, 0.93) and 0.91 (0.85, 0.97)], and butter with olive oil [SRR (95% CI): 0.94 (0.92, 0.97); all moderate CoE]. Mainly no associations were observed when substituting dairy products against each other on disease and mortality risk. Our findings indicate associations between substituting dairy with red or processed meat and higher disease risk, whereas its substitution with whole grains was associated with a lower risk. However, there is little robust evidence that substituting whole-fat with low-fat dairy is associated with disease risk. (CRD42022303198).

## Introduction

Noncommunicable diseases (NCDs) accounted for 73% of deaths worldwide in 2017 [1]. According to the Global Burden of Disease Study, a suboptimal diet leads to 22% of all deaths worldwide [2], with low-milk intake being among the 15 dietary risk factors that characterize a suboptimal diet.

In several Western countries, dietary guidelines recommend 2–3 servings of dairy per day for adults [3]. The EAT-Lancet Commission on Healthy Diets from Sustainable Food Systems suggests 250 mL of whole milk or derivative equivalents per day [4].

In recent systematic reviews of prospective observational studies, each daily serving increase in dairy was not associated with the risk of adiposity [5] but inversely associated with hypertension [6], type 2 diabetes (T2D) [7,8], and stroke [9]. For each daily serving increase in total dairy, whole-fat dairy, low-fat dairy, milk, cheese, and yogurt, no association with coronary artery disease (CAD) was observed [9,10]. These systematic reviews are typically based on studies adjusting their statistical models for total energy intake. The results can, therefore, be interpreted as an isocaloric substitution of dairy with other nonspecified foods [11]. However, this so-called single-exposure model does not account for the fact that changes in dairy intake – under isocaloric conditions – are inevitably accompanied by an in- or decreased intake of other foods and that these foods may vary in their impact on NCD risk [11]. Therefore, substitution models in epidemiologic studies specifying both the substituted foods as well as their substitutes are considered particularly useful to inform dietary guidelines [12]. In recent years, several prospective cohort studies have been published investigating the association of substituting total dairy or specific dairy products with other foods or other dairy products and the risk of all-cause mortality [13,14], cardiovascular disease (CVD) [15,16], and T2D [17,18]. However, to our knowledge, this evidence has not been synthesized and evaluated yet. Therefore, we aimed to conduct a systematic review and meta-analysis on the substitution of dairy products with plant-based foods, other animal-based foods, and specifically other dairy products regarding these health outcomes in the general adult population.

## Methods

We report this systematic review with meta-analysis according to the PRISMA checklist [19] and the PRISMA Statement for Reporting Literature Searches in Systematic Reviews (PRISMA-S) [20]. The protocol of this work was predefined and registered on PROSPERO, registration number CRD42022303198.

### Systematic literature search

We conducted a comprehensive literature search in 3 electronic databases, including MEDLINE (via OVID), Embase (via OVID), and Web of Science (via Clarivate), from inception to 28 June 2023. The search strategy combined 3 search blocks on “dairy products,” “analysis” (e.g., substitution), “study design” (i.e., cohort studies), and was revised by an experienced information specialist. No language filter was applied. The detailed search strategies can be found in Supplementary File 1.

In addition, we conducted backward citation tracking on systematic and narrative reviews identified by our searches, and we screened the reference lists of all included studies.

### Eligibility criteria

We included studies in this systematic review fulfilling the following eligibility criteria:

#### Population

Studies in the general adult population (age ≥18 y) were eligible for inclusion. Studies exclusively investigating children, adolescents, pregnant females, or patients with chronic diseases (e.g., chronic kidney disease, CVD, and T2D) were excluded.

#### Exposure and comparator

Studies were eligible for inclusion that used specified substitution models (i.e., the statistical approach of leave-1-out method or partition method [11]) describing the substitution of dairy products (e.g., total dairy, whole-fat dairy, low-fat dairy, milk, yogurt, and cheese) with all available other food groups (e.g., red meat, processed meat, poultry, fish, vegetable oil, eggs, nuts, and legumes) or the substitution between different types of dairy (e.g., yogurt compared with milk) and products with different fat content (e.g., whole-fat milk compared with low-fat milk). The substitution models can refer to baseline dietary assessment only or multiple dietary assessments [11].

Substitution analyses were only eligible if information on portion size/serving was available.

#### Outcomes

The following outcomes were included: all-cause mortality, CVD, CVD mortality, CAD, stroke, T2D, and T2D mortality.

We deviated from our protocol by not including studies with cancer or cancer mortality as outcomes.

#### Study design

We included prospective observational studies. Cross-sectional studies and case-control studies were excluded.

Detailed eligibility criteria are displayed in Supplementary Table 1.

### Study selection

After deduplication of search hits using Endnote 20 (Clarivate), 2 reviewers from a group of 5 (EK, JS, LS, MN, and SS) screened each title/abstract and full text of potentially eligible studies independently. On the full-text level, reasons for exclusion were recorded (Supplementary Table 2). Any disagreements were resolved by discussion or with the help of a third reviewer if no agreement could be reached. The screening process was implemented using Covidence systematic review software (Veritas Health Innovation, Melbourne, Australia, www.covidence.org).

If multiple publications investigated the same cohort and presented data on the same association, the publication with more outcome events and/or a longer follow-up time was included to avoid duplication.

### Data extraction

After the identification of eligible articles, 2 reviewers (LS and RL) extracted the data independently in a piloted data extraction form (Microsoft Excel). Conflicts were solved by discussion with a third reviewer if no agreement could be reached (EK). We extracted data on study characteristics [i.e., first author, publication year, cohort name, study location (country), study design (i.e., prospective observational study, nested case-control study, case-cohort study), and follow-up duration], number of participants and outcome events, sex and age, outcome and outcome assessment, (dietary) exposure assessment, dairy foods with units (e.g., butter 5 g/d), substitute foods with units (e.g., olive oil 5 g/d), risk estimates (relative risk and hazard ratio) and 95% confidence intervals (95% CI), and adjustment factors [age, sex, energy intake, BMI (in kg/m^2^), smoking, alcohol consumption, education/socioeconomic status, diet, physical activity, family history of disease, and comorbidity]. If studies reported the relevant data only in figures, we used the “Web plot digitizer” (https://automeris.io/WebPlotDigitizer/) for extraction.

### Risk of bias assessment

Two reviewers out of a group of 4 (EK, ES, JS, and MN) assessed the risk of bias of each included study independently and any disagreements were resolved by consensus. We used the Risk Of Bias In Non-randomized Studies - of Exposure (ROBINS-E) tool to evaluate the risk of bias [21]. The tool includes the following 7 domains of bias: *1*) confounding, *2*) measurement of exposure, *3*) selection of participants into the study (or into the analysis), *4*) postexposure interventions, *5*) missing data, *6*) measurement of the outcome, and *7*) selection of the reported result. We judged each domain as well as the overall risk of bias as low risk of bias, some concerns, or high risk of bias. Due to the study design of the included studies, a very high risk of bias was not assigned to single bias domains. Details of the ROBINS-E assessment are provided in Supplementary Table 3.

### Statistical analysis

Meta-analyses were conducted if results of ≥2 studies were available reporting the same exposure, comparator, and outcome. For each substitution meta-analysis, we calculated summary relative risks (SRR) with their 95% CIs based on relative risks and hazard ratios from individual studies (Supplementary Tables 4 and 5). Random-effects models were applied that take both within- and between-study variability into account [22]. To ensure comparability of the results, we converted relative risks and 95% CIs for standardized food portions as previously applied for the substituted dairy foods (dairy, milk, yogurt to 200 g/d; cheese to 30g/d; butter to 5g/d) [23]. Likewise, we recalculated the portion size of the substitute according to the conversion of the substituted food. For example, if a study substituted 70 g/d of yogurt with 50 g/d of red meat, we calculated the relative risk and 95% CI for a substituted portion of 200 g/d and for a substitute of 143 g/d. For the analyses, we made the assumption of monotonic associations between both the amount of dairy products consumed and their substitutes and the risk of NCD or mortality.

We calculated I^2^ and tau^2^ as measures of the inconsistency and between-study variability in the risk estimates. In addition, we computed 95% prediction intervals (95% PI) to show the range within which the true effect of future studies lies with 95% certainty [24].

In some included publications, pooled analyses of multiple cohorts were reported. In case only these pooled risk estimates were available, and no further relevant studies were identified, we extracted and presented these pooled risk estimates (“extracted pooled results”). If substitution analyses were only reported by a single study, we present effect estimates as single study findings.

In our protocol, we planned to perform additional sensitivity analyses excluding studies rated as high risk of bias and, if applicable, subgroup analyses by sex, region, and dietary assessment method. However, due to the limited number of studies, we were unable to conduct any additional analysis. Similarly, due to the low number of studies, funnel plots and Egger’s test to evaluate publication bias were not performed [25,26]. Statistical analyses were conducted using Stata 15.1.

### Certainty of evidence

For each association, 1 investigator (LS) evaluated the certainty of evidence (CoE) by using the Grading of Recommendations Assessment, Development, and Evaluations approach (GRADE/software GRADEpro) [27,28]. The results were reviewed by a second investigator (EK) and any disagreements were solved by discussion. By using the ROBINS-E tool, the initial CoE level is “high” for observational studies. However, the CoE can be downgraded (≤3 levels) due to the risk of bias, inconsistency, indirectness, imprecision, and publication bias. Large effects and a dose-response gradient are the other considered domains [29]. The CoE is classified as high, moderate, low, or very low [29]. The GRADE ratings are presented in evidence profiles and contain information on the type of comparison, the number of included studies, the study design, the number of participants and cases, relative and absolute effect estimates with 95% CI, the overall rating, and the domain-specific judgments with explanations for down- or upgrading as informative footnotes.

## Results

**FIGURE 1:**
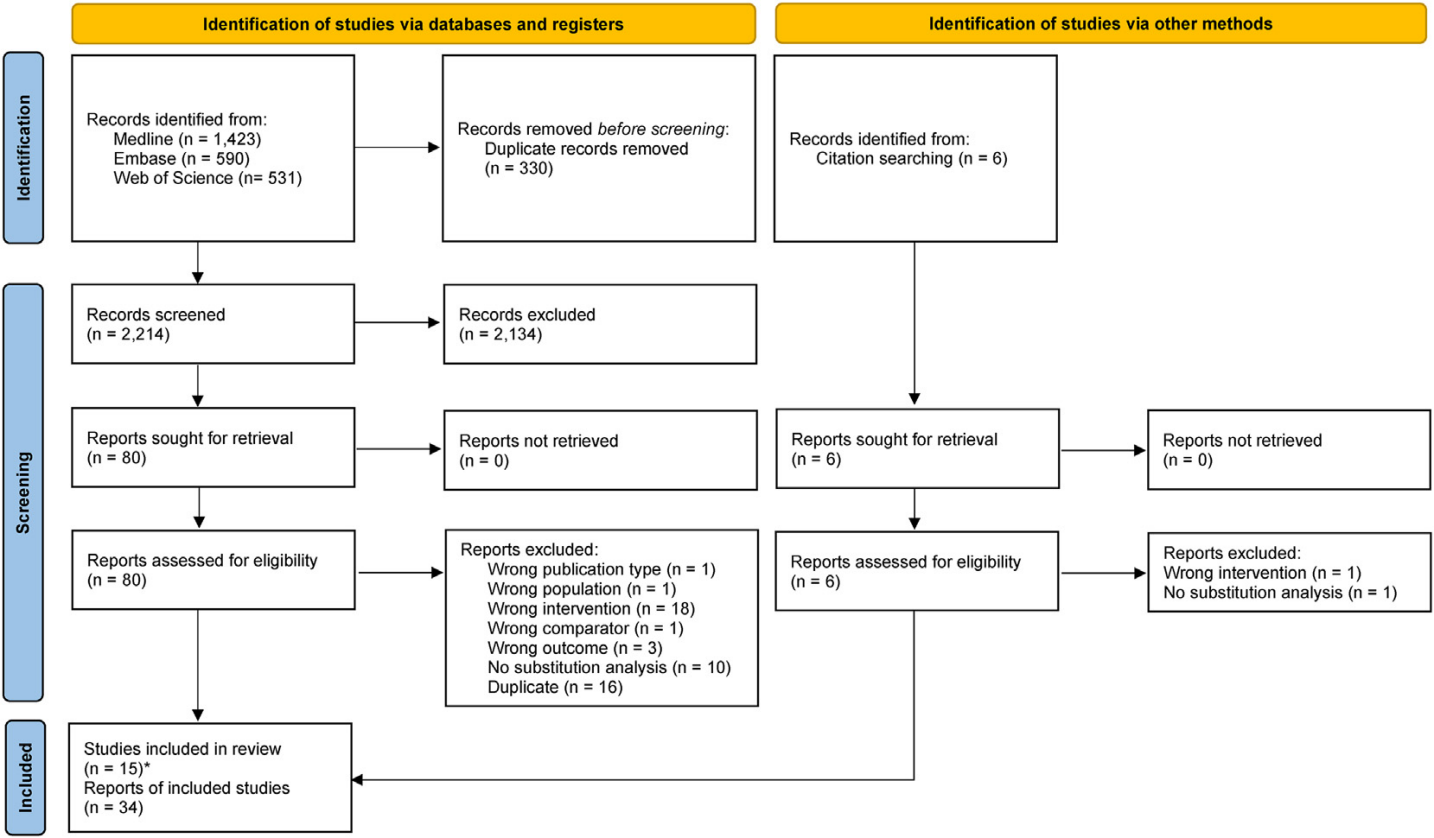
PRISMA 2020 flow diagram. PRISMA, preferred reporting items for systematic reviews and meta-analyses. ∗Refers to the different cohort studies included.Reproduced from reference [19] with permission. For more information, visit: http://www.prisma-statement.org/.

The database searches resulted in 2544 hits. After excluding duplicates, we screened the eligibility for 2214 titles/abstracts and 80 full texts. Finally, we included 34 publications from 15 cohort studies in the systematic review [[13], [14], [15], [16], [17], [18],[30], [31], [32], [33], [34], [35], [36], [37], [38], [39], [40], [41], [42], [43], [44], [45], [46], [47], [48], [49], [50], [51], [52], [53], [54], [55], [56], [57]]. Reasons for the exclusion of full texts are given in Supplementary Table 2. The flow of the search and screening process is depicted in Figure 1.

In Supplementary Table 4**,** the characteristics of included studies investigating the substitution of dairy products with other foods or dairy products based on a single dietary assessment at baseline (*n* = 18; [14,18,[34], [35], [36], [37], [38], [39], [40], [41], [42],^44^,[51], [52], [53], [54], [55],^57^]) or on multiple dietary assessments at certain time intervals (*n* = 16; i.e., using cumulative mean intake or updated intake [13,[15], [16], [17],[30], [31], [32], [33],^43^,[45], [46], [47], [48], [49], [50],^56^]) are presented. Nine cohort studies were conducted in the United States, 4 studies in Europe, and 2 studies in Asia. The mean follow-up duration was 18.1 (range 8–32 y) y. All cohorts except for 7 included both males and females. Three cohorts (Alpha-Tocophenol and Beta-Carotene Cancer Prevention Study (ATBC), Health Professionals Follow-Up Study (HPFS), and Kuopio Ischaemic Heart Disease Risk Factor Study (KIHDRF)) only included males, and 4 cohorts (The Iowa Women's Health Study (IWHS), Nurses Health Study (NHS), NHS II, and Women’s Health Initiative (WHI)) were only females. In all cohorts except for 2 (using consecutive 24-h recalls or 4-d food records [54,56]), the diet was assessed using validated food-frequency questionnaires, sometimes in combination with other methods (e.g., diet history interviews or 24-h recalls [34,35,37]).

**FIGURE 2:**
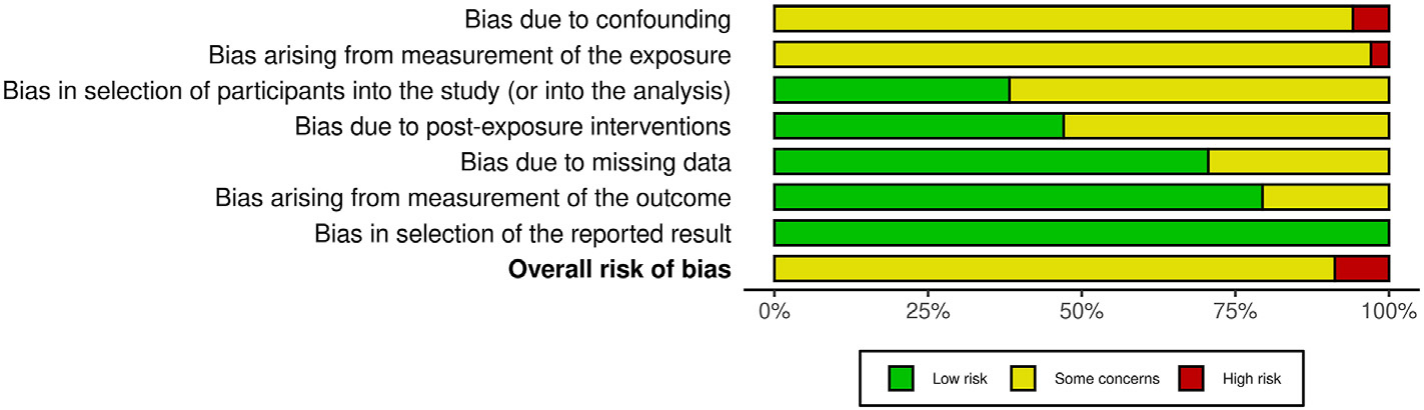
Overall risk of bias of the included studies assessed with the ROBINS-E tool. ROBINS-E, Risk Of Bias In Non-randomized Studies - of Exposure.

Thirty-one studies were judged as being at moderate risk of bias, and 3 studies were judged as being at high risk of bias [17,49,54] (Supplementary Figure 1). Overall, 5.9% of the studies [17,49] were rated as being at high risk of bias in the confounding domain (Figure 2), indicating that the majority of studies adjusted for the most relevant confounders: sex, age, education/socioeconomic status, smoking, BMI, physical activity and total energy intake. One study each did not adjust for BMI [17] and total energy intake [49]. One study (2.9%) was rated as being at high risk of bias due to the measurement of the exposure because the validation of the used instrument to assess food intake was unclear [54].

Out of the 34 publications, 25 were included in meta-analyses. Nine publications could not be included in a meta-analysis because there were <2 studies available for the same exposure [14,37,38,[42], [43], [44],[52], [53], [54]].

### All-cause mortality

A summary forest plot presenting the pooled estimates from substitution meta-analyses regarding all-cause mortality (*n*_cohorts_ = 2–3 per comparison) is shown in Figure 3. Forest plots of individual studies can be found in Supplementary Figures 2–11.

**FIGURE 3:**
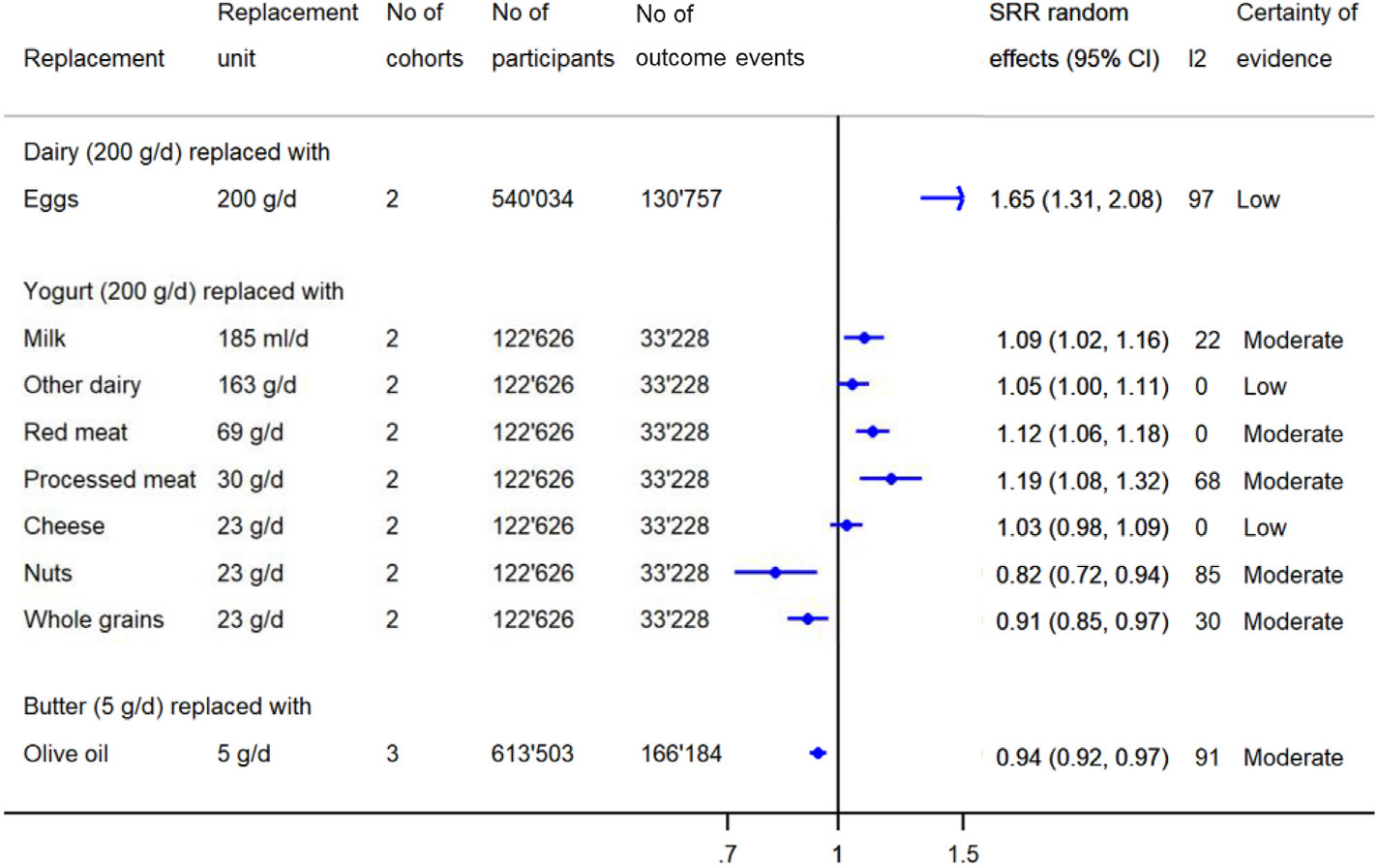
Summary forest plots presenting the pooled results from meta-analyses substituting dairy products with other foods regarding all-cause mortality (replacement and substitution are used synonymously). CI, confidence interval; SRR, summary relative risk.

We found moderate CoE for a lower risk of all-cause mortality associated with the substitution of butter (5 g/d) with olive oil (5 g/d), yogurt (200 g/d) with nuts (23 g/d), yogurt (200 g/d) with whole grains (23 g/d), and dairy (200 g/d) with whole grains (30 g/d) [SRR (95% CI): 0.94 (0.92, 0.97); 0.82 (0.72, 0.94); 0.91 (0.85, 0.97); and 0.89 (0.84, 0.93), respectively] (Figure 3 and Supplementary Figure 2).

There was moderate CoE for higher risk of all-cause mortality associated with the substitution of low-fat dairy (200 g/d) with red meat (142 g/d) [SRR (95% CI): 1.11 (1.06, 1.16)] or processed red meat (85 g/d) [SRR (95% CI): 1.19 (1.11, 1.28)], and of yogurt (200 g/d) with red meat (69 g/d) [SRR (95% CI): 1.12 (1.06, 1.18)], or processed meat (30 g/d) [SRR (95% CI): 1.19 (1.08, 1.32)] (Figure 3 and Supplementary Figure 2). The other associations were rated as low CoE (Supplementary Table 5).

### CVD

Figure 4 and the figures in the supplement (Supplementary Figures 12–26) show the findings on CVD (n_cohorts_ = 2–3 per comparison), CVD mortality (n_cohorts_ = 2–3 per comparison), CAD (n_cohorts_ = 2–6 per comparison), and stroke (n_cohorts_ = 2 per comparison).

**FIGURE 4:**
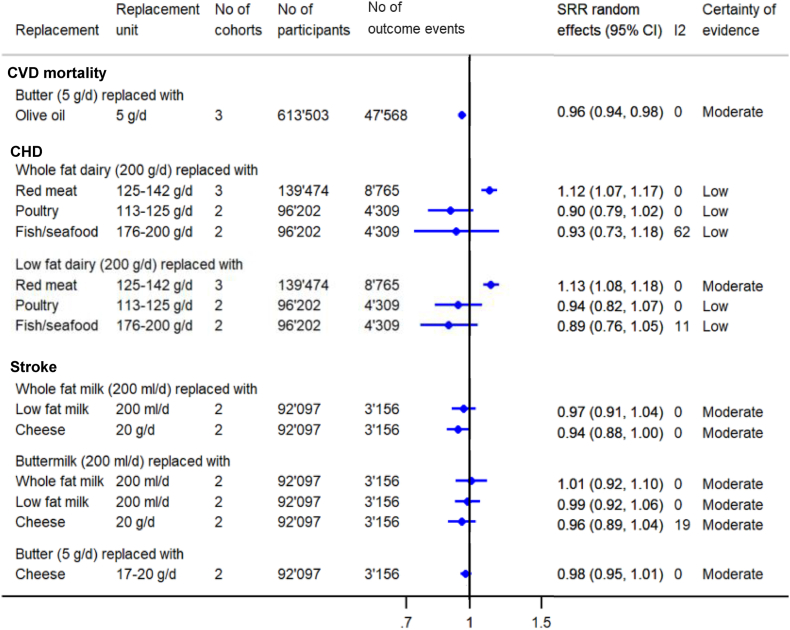
Summary forest plots presenting the pooled results from meta-analyses substituting dairy products with other foods regarding CVD outcomes (replacement and substitution are used synonymously). CI, confidence interval; CVD, cardiovascular disease; SRR, summary relative risk.

Regarding CVD mortality, there was moderate CoE that substituting butter (5 g/d) with the same amount of olive oil was associated with a lower risk of CVD mortality [SRR (95% CI): 0.96 (0.94, 0.98)] (Figure 4). The other associations were rated as low or very low CoE (Supplementary Tables 6 and 7).

There was moderate CoE that substituting low-fat dairy (200 g/d) with red meat (125–142 g/d) was associated with a higher CAD risk [SRR (95% CI): 1.13 (1.08, 1.18)] (Figure 4) and that substituting cheese (30 g/d) with an equal amount of avocado was inversely associated with CAD risk [SRR (95% CI): 0.81 (0.72, 0.90)] (Supplementary Table 8 and Supplementary Figure 13).

There was moderate CoE that substituting both whole- and low-fat dairy products (200 g/d) with red meat (125 g/d) was associated with a higher risk of stroke [SRR (95% CI): 1.10 (1.04, 1.17); 1.11 (1.04, 1.17)], and that substituting different types of dairy (i.e., whole-fat milk, low-fat milk, cheese, buttermilk, and butter) against each other or butter (5 g/d) with equal amounts of olive oil or avocado was not associated with risk of stroke (Figure 4 and Supplementary Figure 13). There were no clear associations found based on the other meta-analyses and the CoE was rated as low (Supplementary Table 9).

### T2D

A summary forest plot presenting the pooled estimates from substitution meta-analyses regarding T2D incidence (*n*_cohorts_ = 2–3 per comparison) is shown in Figure 5, and forest plots of individual studies are in Supplementary Figures 27–47.

**FIGURE 5:**
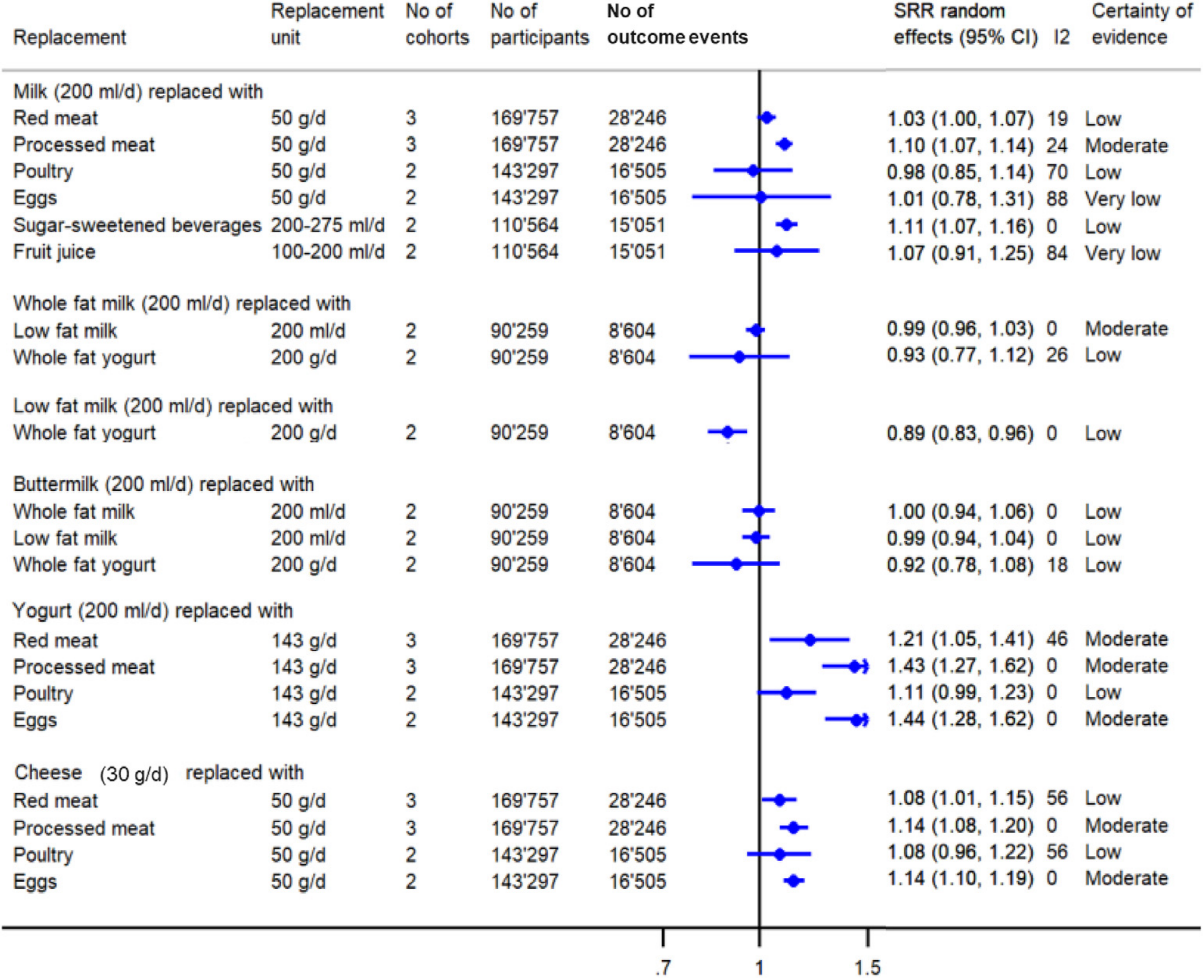
Summary forest plots presenting the pooled results from meta-analyses substituting dairy products with other foods regarding type 2 diabetes incidence (replacement and substitution are used synonymously). CI, confidence interval; SRR, summary relative risk.

There was moderate CoE that substituting low-fat dairy (200 g/d) with red meat (142 g/d) [SRR (95% CI): 1.20 (1.16, 1.25)], processed red meat (57 g/d) [SRR (95% CI): 1.41 (1.33, 1.49)], and unprocessed red meat (142 g/d) [SRR (95% CI): 1.19 (1.15, 1.23)] was associated with a higher risk of T2D incidence (Supplementary Figure 27). No association was observed when dairy (200 g/d) was substituted with whole grains (30 g/d) or nuts/peanuts (28 g/d) (Supplementary Figure 27). For different types of dairy, there was moderate CoE that substituting milk (200 g/d) with processed meat (50 g/d) [SRR (95% CI): 1.10 (1.07, 1.14)], yogurt (200 g/d) with red meat, processed meat or eggs (all 143 g/d) [SRR (95% CI): 1.21 (1.05, 1.41); 1.43 (1.27, 1.62); 1.44 (1.28, 1.62)] or cheese (30 g/d) with processed meat or eggs (both 50 g/d) [SRR (95% CI): 1.14 (1.08, 1.20); 1.14 (1.10, 1.19)] was associated with a higher risk of T2D incidence (Figure 5). No association was observed when whole-fat milk (200 g/d) was substituted with an equal amount of low-fat milk (Figure 5). The remaining associations were rated as low or very low (Supplementary Table 10).

### Statistical heterogeneity

I^2^ is reported in FIGURE 3, FIGURE 4, FIGURE 5, presenting the summary pooled results from the substitution meta-analyses, and in addition, in the forest plots of the individual studies in Supplementary Figures 3–11, 14–26, and 28–47. The calculation of the 95% PI was possible for 8 substitution meta-analyses (Supplementary Figures 17, 20, 32, 34, 38, 40, 45, and 47) as ≥3 studies were included. All calculated 95% PIs crossed the null effect, indicating that the true effect of future studies could be null or point in the opposite direction compared to the results of the meta-analyses.

## Discussion

In the present systematic review and meta-analysis, we provided a comprehensive overview of 15 prospective cohort studies (34 publications) investigating associations of substituting dairy with other foods or other dairy products on cardiometabolic diseases, all-cause, and cardiometabolic mortality risks.

There was moderate CoE for an association with a higher risk of all-cause mortality, CAD, and T2D when substituting low-fat dairy with red meat. The substitution of low-fat dairy with processed meat was also associated with a higher risk of T2D. Substituting cheese or butter with avocado was associated with lower CVD risk. A lower risk of all-cause mortality was observed when substituting dairy and yogurt with whole grains and butter with olive oil. For the other associations, especially regarding the substitution of dairy products such as yogurt, milk, cheese, and buttermilk against each other, the CoE was mainly low or very low.

### Comparison with other studies

To our knowledge, only very few systematic reviews have been published focusing on associations of substituting different food groups with each other and the risk of NCDs or mortality. Neuenschwander et al. [23] recently evaluated the meta-evidence on the substitution of animal-based with plant-based foods regarding cardiometabolic health outcomes. Similar to our results, they found that substituting butter with olive oil is probably associated with a lower risk of CVD, CVD mortality, diabetes, and all-cause mortality (moderate CoE) [23]. In contrast to our work, they did not explicitly investigate the association of dairy substitution with all other available food groups and different types of dairy products against each other.

Hidayat et al. [58] investigated whether substituting red meat with other protein sources is associated with lower risks of CAD and all-cause mortality and observed associations when processed red meat was substituted with dairy. These associations were rated with moderate certainty using the NutriGrade scoring system [59]. However, Hidayat et al. [58] did neither assess the risk of bias with the ROBINS-E tool nor did they investigate further NCD outcomes, such as CVD and T2D.

Other recent systematic reviews on observational studies investigating the association of dairy and NCDs focused on dose-response analyses. Regarding T2D [7,8] and stroke [9], inverse associations were shown for each daily serving increase in dairy intake, whereas no association with CAD was found for each daily serving increase in total dairy, whole-fat dairy, low-fat dairy, milk, cheese, and yogurt intake [9,10]. Moreover, dairy intake (per serving increase) showed a neutral association with all-cause mortality [60] and CVD mortality [60,61], whereas each additional serving of yogurt per day was associated with a reduced risk of all-cause (by 7%) and CVD mortality (by 14%) [62]. Previous meta-analyses also suggest that higher meat intake, especially processed meat, was associated with an increased risk of T2D [7], CAD, and stroke [10], as well as all-cause mortality [63]. This also applies to the inverse associations between olive oil and CVD, T2D, and all-cause mortality [64,65]. In contrast, a higher intake of eggs was not associated with these outcomes in previous studies [7,10,63]. However, the results of all these systematic reviews are not directly comparable to our findings as they refer to single-exposure models not considering the potential differential influence of dairy depending on the foods substituted for dairy. Therefore, the findings of the present systematic review and meta-analysis might be more suited to inform dietary recommendations than those of previous (dose-response) meta-analyses due to a more practical interpretation.

### Possible mechanisms

There are different mechanisms that may explain the observed associations. First, persons consuming less red and processed meat likely follow a healthier lifestyle in general. However, all included studies except 1 adjusted for important lifestyle factors such as total energy intake, physical activity, alcohol intake, as well as smoking, and the associations persisted. Moreover, low-fat dairy products are established indicators of a high-quality diet and are an important component of the “Healthy Eating Index” and the “Dietary Approaches to Stop Hypertension” pattern [66], whereas the intake of red and processed meat is discouraged according to these diet quality indicators [66].

Compared with ingredients of red and processed meat, active ingredients found in dairy products might explain some health benefits on CVD and T2D outcomes. For example, potassium and calcium [67], or lacto-tripeptides [68], are components of dairy products that may contribute to antihypertensive effects, with high-blood pressure being the most important modifiable risk factor for CVD and stroke [69]. Derived from casein, these lacto-tripeptides are proposed to inhibit the activity of the angiotensin I–converting enzyme and may improve the regulation of blood pressure [70]. Moreover, milk fat – mainly present as milk fat globules in bovine milk – with its complex fatty acid composition is discussed in the protection of cardiometabolic health by affecting various biological pathways, including lipid metabolism and inflammatory response [71]. In contrast, red and processed meat contains compounds such as sodium, nitrates and nitrites, heme-iron, and saturated fatty acids, including stearic and palmitic acid, which may increase the risk of CVD and T2D [[72], [73], [74], [75]].

Nuts and whole grains, as well as olive oil, may be favorably compared with dairy products because they contain high amounts of antioxidative and anti-inflammatory compounds, including dietary fiber, phytochemicals, vitamins and minerals, and polyphenols that show beneficial associations with cardiovascular health [76]. Another potential mechanism of action on NCD risk is the association of food groups with body weight. Although whole grains might show favorable associations with anthropometric measures, dairy products showed a neutral association, and red and processed meat might be associated with a higher adiposity risk [5].

### Strengths and limitations

Our systematic review with meta-analysis has several strengths and limitations that need to be considered. Among the strengths are the large number of considered diet-disease associations, the a priori deposited protocol, the comprehensive search strategy, the ROBINS-E assessment, and the GRADE CoE assessment. Moreover, we used standardized portions for the substituted foods, which increased the comparability of the results.

However, our work also has several limitations. First, for all but 3 substitution comparisons, only 2–3 cohort studies were available for the meta-analyses, not allowing conducting subgroup and sensitivity analyses as well as the assessment of publication bias. As most included cohorts were from the United States or Europe, a global perspective on the topic is limited. Second, we were not able to assume causality for the identified associations because of the observational nature of the included studies. Moreover, substitution models of most included studies referred to a single dietary assessment at baseline, not considering dietary changes over time [11]. Therefore, results may be interpreted as theoretical rather than an actual substitution of foods. Third, all analyses were based on the assumption of a monotonic relationship. However, this may not fully capture the functional form of the relationships between the analyzed food groups and all-cause mortality [63], as well as NCDs [7,10]. Fourth, the portion sizes used in the substitution models varied between the included studies and a standardization to usual portion sizes was only possible for the substituted dairy foods (e.g., 200 g/d for dairy) but not for the food substitutes as well. Moreover, due to the differences between studies, in some meta-analyses, a comparison of different amounts of food substitution was necessary. Fifth, when interpreting the results, we need to consider that the studies included in the meta-analyses modeled substitutions in grams per day and adjusted for total energy intake, implying that a difference in energy intake between the substituted foods needs to be compensated by the intake of other foods. In addition, adjustment for food intake varied between included primary studies, which limits the comparability regarding the underlying dietary patterns [11]. Dietary patterns and food choices, moreover, may be closely linked to other demographic, health-related, and lifestyle factors. Although we had defined several potential confounders a priori, and all but 2 studies performed the corresponding adjustments, residual and unmeasured confounding cannot be excluded [12]. Therefore, no study could be judged as low risk of bias in ROBINS-E, leading to a downgrade in the GRADE risk of bias domain.

## Conclusions

In summary, there is little robust evidence that substituting milk, buttermilk, yogurt, cheese, or butter against each other, or whole-fat with low-fat dairy, is associated with chronic disease risk. However, our findings indicate that substituting dairy with red and/or processed meat is probably associated with a higher disease risk, especially for CAD, stroke, and all-cause mortality. In general, we found that substituting dairy foods with plant-based foods may be associated with a lower disease risk. The substitution of dairy products with whole grains, nuts, legumes, avocado, or olive oil mostly showed inverse associations with all-cause mortality, CVD, and T2D. However, more robust evidence is needed to strengthen the certainty of existing associations regarding dairy substitution. In addition, future studies should focus more on dietary changes over time to better address the time at risk until developing NCDs.

## Author contributions

The authors’ responsibilities were as follows – EK: conceptualization, screening, data extraction, risk of bias assessment, Grading of Recommendations, Assessment, Development and Evaluations (GRADE) assessment, drafting the manuscript; MN: conceptualization, screening, risk of bias assessment, statistical analysis, revision of the manuscript; JS: conceptualization, search, deduplication of search results, screening, risk of bias assessment, revision of the manuscript; ES: risk of bias assessment, revision of the manuscript; LH: data extraction, revision of the manuscript; CR, HH: acquisition of funding, conceptualization, revision of the manuscript; KS, MK, SS: conceptualization, revision of the manuscript; LS: acquisition of funding conceptualization, screening, data extraction, GRADE assessment, drafting the manuscript, supervision; EK, LS: are responsible for design, writing, and final content; and all authors: read and approved the final manuscript.

## Conflict of interest

MK is CEO and founder of Kussmann Biotech GmbH. HH is a member of the advisory board of the Competence Center for Nutrition of the Bavarian State Ministry for Food, Agriculture and Forestry and a member of the Food-Based Dietary Guidelines working group of the German Nutrition Society. LS is a member of the Grading of Recommendations, Assessment, Development, and Evaluations working group. All other authors report no conflicts of interest.

## Funding

This project was funded by the Bavarian State Ministry of Nutrition, Agriculture and Forestry [StMELF (Bayerisches Staatsministerium für Ernährung, Landwirtschaft und Forsten)].

## Data availability

This manuscript makes use of publicly available data from published studies; therefore, no original data are available for sharing. All extracted data from the datasheet, which included study and participant characteristics as well as study results, are provided in Supplementary Table 4.
