# Supplementary material for: Substitution of Dairy Products and Risk of Death and Cardiometabolic Diseases: A Systematic Review and Meta-Analysis of Prospective Studies
Source: Curr Dev Nutr. 2024 Apr 23;8(5):102159. doi: 10.1016/j.cdnut.2024.102159 (PMC11108848; doi:10.1016/j.cdnut.2024.102159)
Supplement: Multimedia component 1 [file mmc1.pdf]

Substitution of dairy products and risk of death and cardiometabolic diseases: a systematic review and meta-analysis of prospective studies

Eva Kiesswetter[1*], Manuela Neuenschwander[2,3*], Julia Stadelmaier[1], Edyta Szczerba[2], Lara Hofacker[1], Kathrin Sedlmaier[4], Martin Kussmann[4,5], Christine Roeger[4], Hans Hauner[6,7], Sabrina Schlesinger[2,3], Lukas Schwingshackl[1]

[1]Institute for Evidence in Medicine, Medical Center & Faculty of Medicine, University of Freiburg, Freiburg, Germany.

[2]German Diabetes Center, Institute for Biometrics and Epidemiology, Düsseldorf, Germany.

[3]German Center for Diabetes Research (DZD), Munich-Neuherberg, Partner Düsseldorf Germany.

[4]Competence Center for Nutrition, Bavarian State Ministry for Nutrition, Agriculture and Forestry, Freising, Germany.

[5]Kussmann Biotech GmbH, Nordkirchen, Germany

[6]Else Kröner-Fresenius-Center for Nutritional Medicine, ZIEL – Institute for Food and Health, Technical University of Munich, Freising, Germany.

[7]Institute of Nutritional Medicine, School of Medicine, Technical University of Munich, Munich, Germany.

* contributed equally

Corresponding author:

Dr. Eva Kiesswetter

Breisacher Str. 86, 79110 Freiburg, Germany

Phone: +49(0)761 270-85327

Email: eva.kiesswetter@uniklinik-freiburg.de

Table of Contents

Supplementary file 1: Search terms 5

Supplementary table 1: Eligibility criteria by the PECOS statement 7

Supplementary table 2: List of excluded studies 8

Supplementary table 3: Description and decision criteria for each domain in ROBINS-E....... 9

Supplementary table 4: Characteristics of the included studies investigating whether substituting dairy product (e.g. milk) with another food (e.g. red meat) or another dairy product (e.g. cheese) are related to chronic disease risk. 14

Supplementary figure 1: Risk of bias of each study for each domain and overall 40

Supplementary table 5: GRADE assessment for the substitution analyses regarding all-cause mortality 41

Supplementary table 6: GRADE assessment for the substitution analyses regarding fatal and non-fatal cardiovascular disease 47

Supplementary table 7: GRADE assessment for the substitution analyses regarding cardiovascular disease mortality 49

Supplementary table 8: GRADE assessment for the substitution analyses regarding fatal and non-fatal coronary artery disease 51

Supplementary table 9: GRADE assessment for the substitution analyses regarding fatal and non-fatal stroke 56

Supplementary table 10: GRADE assessment for the substitution analyses regarding type 2 diabetes 61

Supplementary figure 2: Extracted pooled results for all-cause mortality 70

Supplementary figure 3: Meta-analysis comparing the substituting effect of substituting butter with an equal amount of olive oil on all-cause mortality 71

Supplementary figure 4: Meta-analysis comparing the substituting effect of substituting dairy with an equal amount of eggs on all-cause mortality 72

Supplementary figure 5: Meta-analysis comparing the substituting effect of substituting yogurt with cheese on all-cause mortality 73

Supplementary figure 6: Meta-analysis comparing the substituting effect of substituting yogurt with milk on all-cause mortality 74

Supplementary figure 7: Meta-analysis comparing the substituting effect of substituting yogurt with nuts on all-cause mortality 75

Supplementary figure 8: Meta-analysis comparing the substituting effect of substituting yogurt of other dairy on all-cause mortality 76

Supplementary figure 9: Meta-analysis comparing the substituting effect of substituting yogurt with processed meat on all-cause mortality 77

Supplementary figure 10: Meta-analysis comparing the substituting effect of substituting yogurt with red meat on all-cause mortality 78

Supplementary figure 11: Meta-analysis comparing the substituting effect of substituting yogurt with whole grains on all-cause mortality 79

Supplementary figure 12: Extracted pooled results for cardiovascular disease (fatal and non-fatal) 80
Supplementary figure 13: Extracted pooled results for coronary a disease (fatal and non-fatal), and stroke (fatal-non-fatal) 81
Supplementary figure 14: Meta-analysis comparing the substituting effect of substituting butter with an equal amount of olive oil on cardiovascular disease risk 82
Supplementary figure 15: Meta-analysis comparing the substituting effect of substituting low-fat dairy with fish/seafood on coronary artery disease risk 83
Supplementary figure 16: Meta-analysis comparing the substituting effect of substituting low-fat dairy with poultry on coronary artery disease risk 84
Supplementary figure 17: Meta-analysis comparing the substituting effect of substituting low-fat dairy with red meat on coronary artery disease risk 85
Supplementary figure 18: Meta-analysis comparing the substituting effect of substituting whole-fat dairy with fish/seafood on coronary artery disease risk 86
Supplementary figure 19: Meta-analysis comparing the substituting effect of substituting whole-fat dairy with poultry on coronary artery disease risk 87
Supplementary figure 20: Meta-analysis comparing the substituting effect of substituting whole-fat dairy with red meat on coronary artery disease risk....88
Supplementary figure 21: Meta-analysis comparing the substituting effect of substituting butter with cheese on stroke risk 89
Supplementary figure 22: Meta-analysis comparing the substituting effect of substituting buttermilk with cheese on stroke risk 90
Supplementary figure 23: Meta-analysis comparing the substituting effect of substituting buttermilk with an equal amount of low-fat milk on stroke risk....91
Supplementary figure 24: Meta-analysis comparing the substituting effect of substituting buttermilk with an equal amount of whole-fat milk on stroke risk....92
Supplementary figure 25: Meta-analysis comparing the substituting effect of substituting whole-fat milk with cheese on stroke risk....93
Supplementary figure 26: Meta-analysis comparing the substituting effect of substituting whole-fat milk with an equal amount of low-fat milk on stroke risk 94
Supplementary figure 27: Extracted pooled results for type 2 diabetes risk 95
Supplementary figure 28: Meta-analysis comparing the substituting effect of substituting buttermilk with an equal amount of low-fat milk on type 2 diabetes risk 96
Supplementary figure 29: Meta-analysis comparing the substituting effect of substituting buttermilk with an equal amount of whole-fat milk on type 2 diabetes risk 97
Supplementary figure 30: Meta-analysis comparing the substituting effect of substituting buttermilk with an equal amount of whole-fat yogurt on type 2 diabetes risk....98
Supplementary figure 31: Meta-analysis comparing the substituting effect of substituting cheese with eggs on type 2 diabetes risk 99
Supplementary figure 32: Meta-analysis comparing the substituting effect of substituting cheese with processed meat on type 2 diabetes risk....100

Supplementary figure 33: Meta-analysis comparing the substituting effect of substituting cheese with poultry on type 2 diabetes risk 101
Supplementary figure 34: Meta-analysis comparing the substituting effect of substituting cheese with red meat on type 2 diabetes risk 102
Supplementary figure 35: Meta-analysis comparing the substituting effect of substituting low-fat milk with an equal amount of whole-fat yogurt on type 2 diabetes risk 103
Supplementary figure 36: Meta-analysis comparing the substituting effect of substituting milk with eggs on type 2 diabetes risk..104
Supplementary figure 37: Meta-analysis comparing the substituting effect of substituting milk with fruit juice on type 2 diabetes risk 105
Supplementary figure 38: Meta-analysis comparing the substituting effect of substituting milk with processed meat on type 2 diabetes risk 106
Supplementary figure 39: Meta-analysis comparing the substituting effect of substituting milk with poultry on type 2 diabetes risk...107
Supplementary figure 40: Meta-analysis comparing the substituting effect of substituting milk with red meat on type 2 diabetes risk 108
Supplementary figure 41: Meta-analysis comparing the substituting effect of substituting milk with sugar sweetened beverages on type 2 diabetes risk 109
Supplementary figure 42: Meta-analysis comparing the substituting effect of substituting whole-fat milk with an equal amount of low-fat milk on type 2 diabetes risk 110
Supplementary figure 43: Meta-analysis comparing the substituting effect of substituting whole-fat milk with an equal amount of whole-fat yogurt on type 2 diabetes risk................111
Supplementary figure 44: Meta-analysis comparing the substituting effect of substituting yogurt with eggs on type 2 diabetes risk...112
Supplementary figure 45: Meta-analysis comparing the substituting effect of substituting yogurt with processed meat on type 2 diabetes risk 113
Supplementary figure 46: Meta-analysis comparing the substituting effect of substituting yogurt with poultry on type 2 diabetes risk..114
Supplementary figure 47: Meta-analysis comparing the substituting effect of substituting yogurt with red meat on type 2 diabetes risk...115
Supplementary References 116

Supplementary file 1: Search terms

Database(s): **Ovid MEDLINE(R) ALL** 1946 to June 28, 2023
Search Strategy:

#	**Searches**
1	exp dairy products/
2	(((dairy or milk) not (breast-milk or human-milk)) or butter* or ghee or cheese* or cream* or yoghurt* or yogurt* or kefir or buttermilk).ti,ab,kf.
3	1 or 2
4	(substitut* or replac* or exchang*).mp.
5	3 and 4
6	cohort studies/ or follow-up studies/ or longitudinal studies/ or prospective studies/
7	(prospective or cohort* or observational or longitudinal or follow-up or cases or (case* and control*)).ti,ab,kf.
8	6 or 7
9	**5 and 8**

Database(s): **Web of Science Core Collection** via Clarivate; June 28, 2023
Search Strategy:

#	**Searches**
5	#3 and #4
4	TI=(prospective OR cohort? OR observational OR longitudinal OR follow-up OR cases OR (case? AND control?)) OR AB=(prospective OR cohort? OR observational OR longitudinal OR follow-up OR cases OR (case? AND control?))
3	#1 and #2
2	TI=(substitut* OR replac* OR exchang*) OR AB=(substitut* OR replac* OR exchang*)
1	TS=((dairy OR milk OR butter* OR ghee OR cheese* OR cream* OR yogurt* OR yoghurt* OR kefir OR buttermilk) NEAR/9 (intake* OR diet* OR consum* OR nutriti* OR food* OR eat OR eating OR meal*))

Database(s): **EMBASE** via Ovid; June 28, 2023
Search Strategy:

#	**Searches**
1	exp dairy Products/
2	(((dairy or milk) not (breast-milk or human-milk)) or butter* or ghee or cheese* or cream* or yoghurt* or yogurt* or kefir or buttermilk) adj9 (intake* or diet* or consum* or nutriti* or food* or eat or eating or meal*)).tw,kw
3	1 or 2
4	(substitut* or replac* or exchang*).mp.
5	3 and 4
6	cohort studies/ or follow-up studies/ or longitudinal studies/ or prospective studies/
7	(prospective or cohort* or observational or longitudinal or follow-up or cases or (case* and control*)).ti,ab,kf.
8	6 or 7
9	**5 and 8**
10	**limit 9 to embase**

Supplementary table 1: Eligibility criteria by the PECOS statement

	Inclusion criteria	Exclusion criteria
**P** (population)	General healthy adult population	Specific patient populations (patients with chronic diseases such as cardiovascular disease, cancer, and type 2 diabetes), studies on children, adolescents or pregnant women
**E** (intervention/exposure)	Dairy products (e.g. milk, yogurt, cheese, butter) including different fat-content (e.g. Whole-fat vs. low-fat) with information on portion size/serving	Supplements
**C** (comparison)	Substitution of dairy products (e.g. meat, poultry, fish, shellfish, eggs, whole grains, nuts, legumes) with information on portion size/serving<br><br>Substitution of other dairy products (e.g. milk with yogurt) with information on portion size/serving	No substitution
**O** (outcome)	Cardiovascular disease, coronary artery disease, stroke, type 2 diabetes, related morality, all-cause mortality	Biomarkers of cardiometabolic risk (e.g. fasting glucose, blood lipids, etc.)
**S** (study design)	Prospective observational studies (e.g. prospective observational study, nested case-control study, case-cohort study)	In vitro/animal experiments, cross-sectional and retrospective case-control studies

Supplementary table 2: List of excluded studies

Reason for exclusion	References
Wrong publication type	(1)
Wrong population	(2)
Wrong intervention	(3-21)
Wrong comparator	(22)
Wrong outcome	(23-25)
No substitution analysis	(26-36)
Duplicate	(37-52)

Supplementary table 3: Description and decision criteria for each domain in ROBINS-E

Domain	Explanation	Judgements
**Bias due to confounding***	• Is there potential for confounding of the effect of exposure in this study?<br>• Did the authors use a multivariable-adjusted analysis method that controlled at least for age, sex, smoking, physical activity, education/socioeconomic status, alcohol, body mass index, total energy intake and include mutual adjustment of relevant food groups (at least the substituted food groups)?<br>• Did the authors avoid adjusting for post-exposure variables?<br><br>Notes: **Confounding is expected in all observational studies, low risk of bias was not assigned to any study.** Time-varying confounding was expected to be unlikely and is not expected to cause risk of bias in the present study.	<u>Low risk of bias:</u> No bias expected due to confounding, including time-varying confounding.<br><br><u>Some concerns:</u> Confounding is expected: age, sex, smoking, physical activity, education/socioeconomic status, alcohol, body mass index, total energy intake have been appropriately controlled for in a multivariable-adjusted analysis and the model included mutual adjustment of relevant food groups (at least the substituted food groups)<br>*or* confounding is expected: age, sex, smoking, physical activity, alcohol, body mass index, total energy intake have been appropriately controlled for in a multivariable-adjusted analysis and the model included mutual adjustment of relevant food groups (at least the substituted food groups) *and* education/socioeconomic status is not expected to vary substantially within the cohort (e.g. NHS, HPFS)<br>*or* the authors statistically investigated whether the confounding domains have an effect on the risk estimate and excluded the confounder from the multivariable model if there was no effect on the overall effect estimate.<br><br><u>High risk of bias:</u> At least one known important domain was not measured or appropriately controlled for
**Bias arising from measurement of exposure***	• Were exposure groups clearly defined and adequately assessed?<br>• Was the information used to define the exposure groups based on reasonable a priori data?	<u>Low risk of bias:</u> Exposure status was well defined (substitution per serving, plausible substitution portions (e.g. 200 g skimmed milk)); *and* no measurement error is expected in its assessment.

	Note: The start of follow-up is considered to coincide with the baseline exposure assessment. **Any dietary assessment method involves measurement error, thus, no study was assigned low risk of bias.**	Some concerns: Exposure status is well defined (substitution per serving, plausible substitution portions); *and* exposure was measured using a validated tool (e.g. a validated FFQ).<br><br>High risk of bias: Exposure status is not well defined, e.g. implausible substitution portions (e.g. 100 g nuts); *and* exposure was measured using not validated tools.
**Bias due to selection of participants into the study (or into the analysis)***	• Was selection of participants into the study based on participants characteristics observed after start of the study/exposure assessment?<br>• Do start of follow-up and start of exposure coincide for most participants? Were methods used that are likely to correct for the presence of selection biases?<br><br>Notes: In observational studies, it is unlikely that post-exposure variables influenced selection of participants into the study. Exclusion of participants may be mostly based on missing data, which will be considered in the domain referring to missings (see below). The start of follow-up is considered to coincide with the baseline exposure assessment. However, participants are already exposed at start of the study, which might have influenced outcome measured that occurred shortly after start of the study.	Low risk of bias: All participants who would have been eligible for the target stdy were included in the study; *and* the authors conducted a sensitivity analysis excluding cases of the respective outcome (e.g. CVD, mortality, type 2 diabetes) which occurred <2 years after the start of the study and the results did not change.<br><br>Some concerns: Selection into the study may have been related to exposure and outcome (e.g. inclusion of postmenopausal women only); *and* the authors used appropriate methods to correct for the selection bias;<br>*or* the authors conducted no sensitivity analysis excluding cases of the respective outcome (e.g. CVD, mortality, type 2 diabetes) which occurred <2 years after start.<br><br>High risk of bias: Selection into the study was related to exposure and outcome (e.g. only participants with prediabetes were included in the analysis for type 2 diabetes, or specific patients groups for analysis on mortality); *and* this could not be corrected for in the analyses;<br>*or* start of follow up and start of exposure do not coincide *and* the rate ratio is not constant over time.
**Bias due to post-exposure interventions***	• Were there deviations from the exposure beyond what would be expected in usual practice?	Low risk of bias: Repeated measurements of the exposure status during follow-up are available. No or only slight changes were observed and the changes were considered in the analysis.

	• Were these deviations unbalanced between groups and likely to have affected the outcome?<br><br>Notes: Repeated measurements of the exposure are mostly not available in observational studies. It is not expected that there are high changes in diet in healthy participants. Changes in diet may be similar between studies and may also be similar between groups (differential misclassification is not expected). Recent studies have shown that diet is constant or change only slightly over time (53, 54). Thus, if repeated measures are not available, some concerns could be assigned to a study.	<u>Some concerns:</u> Repeated measurements of the exposure are not available, but high changes are not expected during follow-up (compare notes)<br>or repeated measurements of the exposure status during follow-up are available and some changes in lifestyle factors were observed. The analysis was appropriate to estimate the effect of changes in lifestyle factors, allowing for deviations that were likely to impact on the outcome;<br><br><u>High risk of bias:</u> Exposure status is measured during follow-up and high changes in lifestyle factors have been observed, and the analysis was not appropriate to estimate the effect of changes in lifestyle factors, allowing for deviations that were likely to impact on the outcome.
**Bias due to missing data***	• Were there missing outcome data?<br>• Were participants excluded due to missing data on exposure status?<br>• Were participants excluded due to missing data on other variables needed for analysis?<br><br>Notes: Missing data on exposure variables and other variables are expected to be missing at random and not related to exposure or outcome that have been assessed during follow-up.	<u>Low risk of bias:</u> Little loss-to-follow-up (<20%) and data on exposure and other variables were reasonably complete (<10% missing data) and was unlikely to introduce bias;<br>*or* the analysis addressed missing data and is likely to have removed any risk of bias.<br><br><u>Some concerns:</u> There is a proportion of missing data in the original cohort or a high proportion of loss-to-follow-up; *and* the analysis is unlikely to have removed the risk of bias arising from the missing data (e.g. using logistic regression).<br><br><u>High risk of bias:</u> High proportions (>50%) of missing data; *and* the analysis is unlikely to have removed the risk of bias arising from the missing data;<br>*or* missing data were addressed inappropriately in the analysis;<br>*or* the nature of the missing data means that the risk of bias cannot be removed through appropriate analysis.

**Bias arising from measurement of the outcome***	• Could the outcome measure have been influenced by knowledge of the exposure status?<br>• Were the methods of outcome assessment comparable across exposure groups?<br>• Were any systematic error in measurement of the outcome related to exposure status?<br><br>Notes: In observational studies, it is not expected that outcome assessors were aware of exposure status of the participants.	<u>Low risk of bias:</u> The methods of outcome assessment were comparable across exposure groups; *and* the outcome measure was unlikely to be influenced by knowledge of the exposure status of study participants; *and* any error in measuring the outcome is unrelated to exposure status (i.e. objective measures such as confirmed medical records, record linkage and death certificates).<br><br><u>Some concerns:</u> The methods of outcome assessment were comparable across exposure groups; *and* any error in measuring the outcome may be minimally related to exposure status *or* if the outcome measure was not reliable measured (i.e. confirmed records are not available for the whole study population).<br><br><u>High risk of bias:</u> The methods of outcome assessment were not comparable across exposure groups;<br>*or* the outcome measure was subjective (i.e. self-report of CVD, type 2 diabetes, etc. by study participants or next of kin); *and* error in measuring the outcome was related to exposure status.
**Bias in the selection of the result***	• Is the reported effect estimate likely to be selected from multiple analyses of exposure-outcome relationship?<br>• Is the reported effect estimate likely to be selected from different subgroups?<br><br>Notes: In observational studies, it is unusual to publish an a priori analysis plan or protocol. Multiple outcome measurements for the definition of CVD, mortality, type 2 diabetes, etc. are not expected.	<u>Low risk of bias:</u> There is a clear description of all analysis and the analyses are consistent and all reported results correspond to all intended outcomes, analyses and sub-cohorts.<br><br><u>Some concerns:</u> The analyses are clearly defined; *and* there is indication of selection of the reported analysis from among multiple analyses; *and* there is indication of selection of the cohort or subgroups for analysis and reporting on basis of the results (e.g. estimates not shown for all analyses).

		High risk of bias: There is a high risk of selective reporting from among multiple analyses; *or* the cohort or subgroup is selected from a larger study for analysis and appears to be reported based on the results.
**Overall judgement**	Low risk of bias	The study is judged to be at low risk of bias for all domains.
	Some risk of bias	The study is judged to be at low or some concerns of bias for all domains.
	High risk of bias	The study is judged to be at high risk of bias in at least one domain.
	Very high risk of bias	The study is judged to be at high risk of bias in several domains.

*Due to the nature of the included prospective observational studies; studies were not considered to be at very high risk of bias in single bias domains

Supplementary table 4: Characteristics of the included studies investigating whether substituting dairy product (e.g. milk) with another food (e.g. red meat) or another dairy product (e.g. cheese) are related to chronic disease risk.

Author, year, cohort name, country	Follow-up (years)	Number of participants, sex, age	Number of cases	Outcome	Outcome assessment	Exposure assessment	Food (unit)	Substitution (unit)	RR (95% CI)	Adjustment factors
Al-Shaar 2020, HPFS, USA (55)	30	43272, M, 53 years	4456	CAD incidence/ mortality	Self-report, validation via medical records, vital records, National Death Index, reports by kin or postal system	Validated, semi-quantitative FFQ (calculated cumulative mean intake based on multiple assessments)	Total red meat (142 g/d)	Whole-fat dairy (1 serving/d)	0.88 (0.82; 0.94)	Age, race or ethnicity, work status, profession, living arrangement, year of questionnaire return, BMI, smoking status, alcohol intake, physical activity, aspirin use, family history of early coronary heart disease or stroke, marital status multivitamin use, intakes of poultry, fish, egg, combined plant protein sources of nuts, legumes, and soy, whole grains, fruit, vegetables, coffee, total milk, yogurt, cheese, other dairy products, glycemic index, total energy intake.
								Low-fat dairy (1 serving/d)	0.89 (0.84; 0.95)	
								Total milk (1 serving/d)	0.90 (0.85; 0.96)	
								Skimmed milk (1 serving/d)	0.90 (0.85; 0.96)	
								Whole-fat milk (1 serving/d)	0.90 (0.82; 0.99)	
								Yogurt (1 serving/d)	0.78 (0.64; 0.94)	
								Cheese (1 serving/d)	0.89 (0.82; 0.98)	
							Unprocessed red meat (142 g/d)	Whole-fat dairy (1 serving/d)	0.89 (0.81; 0.97)	
								Low-fat dairy (1 serving/d)	0.90 (0.82; 0.98)	
								Total milk (1 serving/d)	0.91 (0.83; 0.99)	
								Skimmed milk (1 serving/d)	0.91 (0.83; 0.99)	
								Whole-fat milk (1 serving/d)	0.90 (0.81; 1.01)	
								Yogurt (1 serving/d)	0.77 (0.63; 0.94)	
								Cheese (1 serving/d)	0.91 (0.81, 1.01)	

							Processed red meat (57 g/d)	Whole-fat dairy (1 serving/d)	0.85 (0.78; 0.93)	
								Low-fat dairy (1 serving/d)	0.86 (0.79; 0.94)	
								Total milk (1 serving/d)	0.87 (0.80; 0.95)	
								Skimmed milk (1 serving/d)	0.87 (0.80; 0.95)	
								Whole-fat milk (1 serving/d)	0.86 (0.77; 0.97)	
								Yogurt (1 serving/d)	0.74 (0.60; 0.90)	
								Cheese (1 serving/d)	0.86 (0.77; 0.96)	
Bernstein 2010, NHS, USA (56)	26	84136, W, 58 years	3162	CAD incidence/ mortality	Self-report, validated by medical records, state vital records, National Death Index, next of kin or postal system, autopsy report, death certificate	Validated, semi-quantitative FFQ (calculated cumulative mean intake based on multiple assessments)	Fish (200 g/d)	Whole-fat dairy (227 g/d)	1.24 (1.01; 1.53)	Age, calendar time, BMI, smoking status, alcohol intake, physical activity, aspirin use, menopausal status, parental history of early myocardial infarction, years of multivitamin use, vitamin E supplement use, cereal fiber, trans unsaturated fatty acids, total energy intake
								Low-fat dairy (227 g/d)	1.23 (0.98; 0.54)	
							Poultry (128 g/d)	Whole-fat dairy (227 g/d)	1.12 (0.94; 1.34)	
								Low-fat dairy (227 g/d)	1.09 (0.90; 1.31)	
							Red meat (142 g/d)	Whole-fat dairy (227 g/d)	0.89 (0.83; 0.95)	
								Low-fat dairy (227 g/d)	0.87 (0.81; 0.94)	
Bernstein 2012, NHS,	22 (M), 26 (W)		4030		Self-report validated by	Validated, semi-	Red meat (142 g/d)	Whole-fat dairy (227 g/d)	0.90 (0.84; 0.96)	Age, calendar time, BMI, smoking,

HPFS, USA (57)		127160, M/W, 62 years (HPFS), 57 (NHS)		Stroke incidence/ mortality	medical records, state vital records, National Death index, next of kin or the postal system	quantitative FFQ (calculated cumulative mean intake based on multiple assessments)		Low-fat dairy (227 g/d)	0.89 (0.83; 0.95)	alcohol, physical activity, aspirin use years of multivitamin use, vitamin E supplement use, parental history of early MI, menopausal status, fruit and vegetables, trans unsaturated fatty acids, included dietary protein sources, cereal fiber, total energy
Ding 2019, NHS, NHS II, HPFS, USA (58)	29-32	217755, M/W, 25-75 years (at baseline)	51438	All-cause mortality	State vital records, national death index, reports from family members and postal authorities	Validated, semi-quantitative FFQ (calculated cumulative mean intake based on multiple assessments)	Total dairy (1 serving/d e.g. 240 ml of milk)	Nuts and legumes (1 serving/d e.g. 28 g/d nuts / 50 g/d legumes)	0.86 (0.78; 0.95)	Age, sex, BMI, smoking status, alcohol consumption, physical activity, baseline disease status, family history of cancer, family history of cardiovascular disease, postmenopausal status, current postmenopausal HRT, overall dietary pattern (alternative healthy eating index score), total energy intake
								Whole grains (1 serving/d e.g. 32 g of bread or 200 g cooked brown rice or cereals)	0.89 (0.84; 0.93)	
								Poultry (1 serving/d)	0.93 (0.85; 1.03)	
								Red and processed meat (1 serving/d)	1.05 (1.01; 1.09)	
			12143	CVD mortality				Nuts and legumes (1 serving/d e.g. 28 g/d nuts / 50 g/d legumes)	0.89 (0.84; 0.93)	

								Whole grains (1 serving/d e.g. 32 g of bread or 200 g cooked brown rice or cereals)	0.93 (0.85; 1.01)	
								Poultry (1 serving/d)	1.01 (0.84; 1.20)	
								Red and processed meat (1 serving/d)	1.08 (1.02; 1.15)	
Guasch-Ferre 2015, NHS, NHS II, USA (59)	22	145087, W, NHS 56 years, NHS II 36 years	9652	T2D incidence	Self-report, validated supplementary questionnaire (symptoms, diagnostic tests, treatment), confirmed according to criteria of National Diabetes Association	Validated FFQ (calculated cumulative mean intake based on multiple assessments)	Butter (8 g/d)	Olive oil (8 g/d)	0.92 (0.87; 0.97)	Age, ethnicity, ancestry, smoking status, alcohol intake, physical activity, family history of diabetes, history of hypertension, history of hypercholesterolemia, postmenopausal status and menopausal HRT, multivitamin use, quintiles of the Alternative Healthy Eating Index score, total energy intake
Guasch-Ferre 2020, NHS, HPFS, USA (60)	24	92978, M/W, 65-67 years	6034	CAD incidence/ mortality	Self-report or report by a family member, validation via medical records, U.S. postal system, death certificates from vital statistics	Validated, semi-quantitative FFQ (calculated cumulative mean intake based on	Butter (5 g/d)	Olive oil (5 g/d)	0.94 (0.89; 1.01)	Age, ethnicity, Southern European and/or Mediterranean ancestry, smoking status, alcohol intake, physical activity, BMI, baseline diabetes, baseline hypertension

			9797	CVD incidence/ mortality	departments, National Death Index, autopsy reports	multiple assessments)			0.95 (0.91; 1.00)	or antihypertensive medication use, baseline hypercholesterolemia or cholesterol-lowering medication use, aspirin use, family history of diabetes, family history of MI, family history of cancer, postmenopausal status and menopausal HRT, red meat, fruits and vegetables nuts, soda, whole grains intake, trans fat, total energy intake, mutually adjusted for other types of fat
			3802	Stroke incidence/ mortality					0.96 (0.89; 1.04)	
Guasch-Ferre 2022, NHS, HPFS, USA (61)	28	92383, M/W, 56 years (NHS), 57 years (HPFS)	36856	All-cause mortality	State vital records, national death index, reports from next of kin and postal authorities	Validated, semi-quantitative FFQ	Butter (10 g/d)	Olive oil (10 g/d)	0.86 (0.84; 0.89)	Age, ethnicity, ancestry, married, living alone, BMI, smoking status, alcohol intake,

			8821	CVD mortality		(used time-updated consumption)			0.93 (0.87; 0.98)	physical, family history of diabetes, MI or cancer, postmenopausal status and menopausal HRT, multivitamin use, aspirin use, red meat, fruits and vegetables, nuts, soda, whole grains, intake of trans fat, mutually adjusted for intake of other types of fat, energy intake
Haring 2014, ARIC, USA (62)	22	12066, M/W, 54 years	1147	CAD incidence/ mortality	Information from study visits, yearly telephone follow-up calls, review of hospital discharge lists and medical charts, death certificates, next-of-kin interviews, and physician-completed questionnaires	FFQ (calculated cumulative mean intake based on multiple assessments)	Processed meat (1 serving/d)	Low-fat dairy (1 serving/d)	0.96 (0.84; 1.11)	Age, sex, race, study, center, education, smoking status, alcohol intake, BMI, waist-to-hip ratio, systolic blood pressure, use of antihypertensive medication, HDL-C, total cholesterol, use of lipid lowering medication, sports-related physical activity, leisure-related physical activity, carbohydrate intake, fiber intake, magnesium intake, intake of processed meat, red meat, poultry, Whole-fat dairy, low-fat dairy,
								Whole-fat dairy (1 serving/d)	1.04 (0.90; 1.20)	
							Red meat (1 serving/d)	Low-fat dairy (1 serving/d)	0.84 (0.68; 1.05)	
								Whole-fat dairy (1 serving/d)	0.91 (0.74; 1.12)	
							Poultry (1 serving/d)	Low-fat dairy (1 serving/d)	1.04 (0.83; 1.30)	
								Whole-fat dairy (1 serving/d)	1.12 (0.90; 1.40)	
							Whole-fat dairy (1 serving/d)	Low-fat dairy (1 serving/d)	0.93 (0.84; 1.02)	
								Fish / seafood (1 serving/d)	0.94 (0.75; 1.19)	
								Eggs (1 serving/d)	0.89 (0.75; 1.06)	
								Nuts (1 serving/d)	0.90 (0.78; 1.03)	
								Legumes (1 serving/d)	0.93 (0.73; 1.19)	

							Low-fat dairy (1 serving/d)	Fish / seafood (1 serving/d)	1.02 (0.80; 1.28)	eggs, nuts, seafood, legumes, total energy intake
								Eggs (1 serving/d)	0.96 (0.81; 1.14)	
								Nuts (1 serving/d)	0.97 (0.84; 1.11)	
								Legumes (1 serving/d)	1.01 (0.79; 1.28)	
Ibsen, 2017, DCH, Denmark (63)	15.3*	54277, W 56 years*, M 55 years*	7137	T2D incidence	Danish National Diabetes Register	Validated FFQ (baseline assessment)	Whole-fat milk (200 g/d)	Low-fat milk (200 g/d)	0.99 (0.96 ;1.03)	Age, sex, educational level, BMI, waist circumference, smoking status, alcohol intake, physical activity, history of hypertension, history of hypercholesterolemia, intake of other food groups, total energy intake
								Low-fat yogurt products (200 g/d)	1.04 (0.96; 1.12)	
								Whole-fat yogurt products (200 g/d)	0.89 (0.82; 0.96)	
								Cheese (20 g/d)	1.00 (0.98; 1.02)	
							Buttermilk (200 g/d)	Low-fat milk (200 g/d)	0.99 (0.94; 1.05)	
								Whole-fat milk (200 g/d)	1.00 (0.94; 1.07)	
								Low-fat yogurt products (200 g/d)	1.04 (0.95; 1.13)	
								Whole-fat yogurt products (200 g/d)	0.89 (0.81; 0.97)	
								Cheese (20 g/d)	1.00 (0.98; 1.02)	
							Whole-fat yogurt products (200 g/d)	Low-fat yogurt products (200 g/d)	1.17 (1.06; 1.29)	
								Cheese (20 g/d)	1.01 (0.99; 1.03)	
							Low-fat milk (200 g/d)	Low-fat yogurt products (200 g/d)	1.05 (0.98; 1.12)	

								Whole-fat yogurt products (200 g/d)	0.89 (0.83; 0.96)	
								Cheese (20 g/d)	1.00 (0.98; 1.02)	
							Low-fat yogurt products (200 g/d)	Cheese (20 g/d)	0.99 (0.97; 1.02)	
							Butter (6 g/d)		0.98 (0.94; 1.02)	
Ibsen, 2020, EPIC-Interact, Europe (64)	12.3	26460, M/W, 47-61 years	11741	T2D incidence	Self-report and/or linkage to primary care, secondary care, drug, hospital admission, mortality data, validation via medical records; Danish or Swedish National Diabetes Register	Validated FFQ or diet history interviews (baseline assessment)	Red and processed meat (50 g/d)	Cheese (30 g/d)	0.90 (0.83; 0.97)	Age, sex, center, education, BMI, smoking status, alcohol intake, physical activity, fruit, vegetables, sweets, soft drinks, coffee, tea, other dairy products, total energy intake
								Yogurt (70 g/d)	0.90 (0.86; 0.95)	
								Milk (200 g/d)	0.95 (0.89; 1.01)	
							Red meat (50 g/d)	Cheese (30 g/d)	0.91 (0.84; 0.98)	
								Yogurt (70 g/d)	0.92 (0.85; 0.99)	
								Milk (200 g/d)	0.96 (0.90; 1.02)	
							Processed meat (50 g/d)	Cheese (30 g/d)	0.85 (0.76;0.96)	
								Yogurt (70 g/d)	0.87 (0.81; 0.93)	
								Milk (200 g/d)	0.91 (0.82; 1.02)	
Imamura, 2019, EPIC-Interact, Europe (65)	15	27662, M/W, 52-65 years	12333	T2D incidence	Self-report and/or linkage to primary care, secondary care, drug, hospital admission, mortality data, validation via medical records; Danish or Swedish National Diabetes Register	Validated FFQ or diet history interviews (baseline assessment)	Sugar-sweetened beverages (250 g/d)	Milk (250 g/d)	0.91 (0.82; 1.02)	Age, sex, education, recruitment centers, BMI, waist circumference, smoking status, alcohol intake, physical activity, hypertension, dyslipidemia, family history of diabetes, prevalent diseases (CHD and stroke), marital status,
							Fruit juice (250 g/d)		1.01 (0.90; 1.18)	
							Milk (250 g/d)	Coffee (250 g/d)	0.85 (0.78; 0.92)	

								Tea (250 g/d)	0.82 (0.74; 0.91)	hormone replacement therapy, menopausal status, history of oral contraceptive use, dietary supplement use, dietary consumption, vegetables, fruits, nuts, cheese, yogurt, red meats, processed meats, fish, confectionary, cereals), mutual adjustment of all beverages, total energy intake,
Keller 2020, HPP (ARIC, ATBC, HPFS, IWHS, WHS, NHS), USA (66)	8.2	284345, M/W, 54.7 (M), 52 years (W)	4248	CAD incidence	Questionnaires supplemented by medical records, autopsy reports or death certificates reviewed by physicians	FFQ (baseline assessment)	Sugar-sweetened beverages (335 ml/d)	Low-fat milk (335 ml/d)	0.96 (0.90; 1.03)	Age, education, calendar year in which the baseline questionnaire was returned, BMI, smoking, alcohol, physical activity, hypertension, high cholesterol, confounding with diet, quintiles of cereal fibers, quintiles of trans-fat, quintiles of poly-unsaturated fat/saturated fat ratio, total energy
								Whole-fat milk (335 ml/d)	0.97 (0.88; 1.06)	
								Total milk (335 ml/d)	0.97 (0.90; 1.03)	
			1630	CAD mortality				Low-fat milk (335 ml/d)	0.99 (0.88; 1.12)	
								Whole-fat milk (335 ml/d)	1.04 (0.90; 1.20)	
								Total milk (335 ml/d)	1.01 (0.90; 1.14)	
Key 2019, EPIC, Europe (67)	12.6	409885, M/W, 52.7 years (M), 51.3 years (W)	7198	CAD incidence/ mortality	Morbidity or hospital registries and self-report followed by confirmation with	Country-specific diet assessment methods, mostly	Red and processed meat (100 kcal/d)	Milk (100 kcal/d)	0.95 (0.90; 1.00)	Age, employment status, education, EPIC center, smoking status and number of cigarettes per day,
								Yogurt (100 kcal/d)	0.84 (0.76; 0.92)	

					medical records and information on vital status from mortality registries or active follow-up	validated FFQ, computerized 24-hour recall method (baseline assessment)		Cheese (100 kcal/d)	0.85 (0.79; 0.92)	alcohol intake, physical activity, BMI, history of diabetes mellitus, pervious hypertension, prior hyperlipidemia, calibrated intakes of energy, fruit and vegetables combined, sugars, fiber from cereals, each other food as appropriate
Kvist 2020, DCH, Denmark (68)	15.9	54903, M/W, 56 years	3033	CAD incidence/ mortality	Linkage with national wide registries (Danish National Patient Register or Danish Register of Causes of Death) validated by a study or individually	Validated, semi-quantitative FFQ (baseline assessment)	Whole-fat milk (200 g/d)	Low-fat milk (200 g/d)	0.97 (0.92; 1.02)	Age, sex, education, date of entry into the study, age at entry into the study, BMI, waist circumference adjusted for BMI, smoking, alcohol intake, physical activity, hypertension, hypercholesterolemia, diabetes, red meat intake, processed meat intake, vegetable intake, fruit intake, intake of total energy
								Low-fat yogurt (200 g/d)	0.88 (0.78; 0.99)	
								Whole-fat yogurt (200 g/d)	0.89 (0.79; 1.00)	
								Cheese (20 g/d)	0.93 (0.88; 0.98)	
							Buttermilk (200 g/d)	Low-fat milk (200 g/d)	1.01 (0.93; 1.10)	
								Whole-fat milk (200 g/d)	1.05 (0.95; 1.14)	
								Low-fat yogurt (200 g/d)	0.92 (0.80; 1.06)	
								Whole-fat yogurt (200 g/d)	0.93 (0.81; 1.07)	
								Cheese (20 g/d)	0.97 (0.89; 1.05)	
							Whole-fat yogurt products (200 g/d)	Low-fat yogurt (200 g/d)	0.99 (0.85; 1.15)	
								Cheese (20 g/d)	1.04 (0.93; 1.17)	

							Low-fat milk (200 g/d)	Low-fat yogurt (200 g/d)	0.91 (0.81; 1.01)	
								Whole-fat yogurt (200 g/d)	0.92 (0.82; 1.02)	
								Cheese (20 g/d)	0.96 (0.92; 0.99)	
							Low-fat yogurt products (200 g/d)	Cheese (20 g/d)	1.05 (0.94; 1.19)	
							Butter (200 g/d)	Cheese (20 g/d)	0.98 (0.95; 1.02)	
Laursen 2018, DCH, Denmark (69)	13.4	55211, M/W, 56.1 years	2272	Stroke incidence/ mortality	Linkage to Danish National Patient Registry	Validated, semi-quantitative FFQ (baseline assessment)	Whole-fat milk (200 g/d)	Low-fat milk (200 g/d)	0.97 (0.91; 1.04)	Age, education, BMI, waist circumference adjusted for BMI, smoking, alcohol intake, physical activity, history of hypertension, hypercholesterolemia, diabetes, pervious MI, intakes of fruit, vegetables, red meat, processed meat, fish, total energy intake
								Semi-skimmed fermented milk (200 g/d)	0.96 (0.84; 1.10)	
								Whole-fat fermented milk (200 g/d)	0.89 (0.77; 1.02)	
								Cheese (20 g/d)	0.93 (0.87; 1.00)	
							Buttermilk (200 g/d)	Low-fat milk (200 g/d)	0.97 (0.88; 1.06)	
								Whole-fat milk (200 g/d)	1.00 (0.90; 1.11)	
								Semi-skimmed fermented milk (200 g/d)	0.96 (0.82; 1.12)	
								Whole-fat fermented milk (200 g/d)	0.89 (0.76; 1.04)	
								Cheese (20 g/d)	0.93 (0.85; 1.02)	
							Whole-fat fermented milk (200 g/d)	Semi-skimmed fermented milk (200 g/d)	1.08 (0.91; 1.28)	
								Cheese (20 g/d)	1.05 (0.92; 1.20)	

							Low-fat milk (200 g/d)	Semi-skimmed fermented milk (200 g/d)	0.99 (0.87; 1.13)	
								Whole-fat fermented milk (200 g/d)	0.91 (0.80; 1.04)	
								Cheese (20 g/d)	0.96 (0.92; 1.01)	
							Semi-skimmed fermented milk (200 g/d)	Cheese (20 g/d)	0.97 (0.85; 1.10)	
							Butter (6 g/d)	Cheese (20 g/d)	0.96 (0.92; 1.00)	
Laursen 2019, EPIC-NL, Netherlands (70)	15.2	36886, M/W, 51.4 years	884	Stroke incidence	Information from standardised register for hospital discharge diagnoses, municipal registries and Statistics Netherlands	Validated, semi-quantitative FFQ (baseline assessment)	Whole-fat yogurt (200 g/d)	Low-fat yogurt (200 g/d)	1.32 (0.73; 2.36)	Energy intake, cohort (stratum variable), education, BMI-adjusted waist circumference, smoking, physical activity, alcohol, Dutch Health Diet Index 2015, hypertension, hypercholesterolemia, diabetes mellitus, MI
							Low-fat milk (200 g/d)		0.91 (0.75; 1.11)	
								Whole-fat yogurt (200 g/d)	0.69 (0.40; 1.20)	
							Whole-fat milk (200 g/d)	Low-fat yogurt (200 g/d)	0.90 (0.70; 1.16)	
								Whole-fat yogurt (200 g/d)	0.68 (0.39; 1.22)	
								Low-fat milk (200 g/d)	0.99 (0.78; 1.25)	
							Buttermilk (200 g/d)	Low-fat yogurt (200 g/d)	0.93 (0.76; 1.14)	
								Whole-fat yogurt (200 g/d)	0.70 (0.41; 1.22)	
								Low-fat milk (200 g/d)	1.02 (0.90; 1.16)	
								Whole-fat milk (200 g/d)	1.03 (0.84; 1.26)	
							Cheese (20 g/d)	Low-fat yogurt (200 g/d)	0.91 (0.76; 1.10)	
								Whole-fat yogurt (200 g/d)	0.69 (0.40; 1.19)	

								Low-fat milk (200 g/d)	1.00 (0.90; 1.12)	
								Whole-fat milk (200 g/d)	1.01 (0.84; 1.23)	
								Buttermilk (200 g/d)	0.99 (0.88; 1.10)	
							Butter (6 g/d)	Low-fat yogurt (200 g/d)	0.91 (0.76; 1.10)	
								Whole-fat yogurt (200 g/d)	0.69 (0.40; 1.19)	
								Low-fat milk (200 g/d)	1.00 (0.90; 1.11)	
								Whole-fat milk (200 g/d)	1.01 (0.83; 1.24)	
								Buttermilk (200 g/d)	0.98 (0.88; 1.10)	
								Cheese (20 g/d)	1.00 (0.92; 1.08)	
Laursen 2022, DCH, Denmark (71)	19	26614, M, 56 years	6812	All-cause mortality	Danish Civil Registration System and Danish Causes of Death register	Validated, semi-quantitative FFQ (baseline assessment)	Whole-fat milk (200 g/d)	Low-fat milk (200 g/d)	0.97 (0.94; 1.00)	Age, education, date of inclusion, BMI, waist circumference adjusted for BMI, smoking, alcohol intake, physical activity, hypertension, hypercholesterolemia, intakes of fruit, vegetables, red meat, processed meat and fish, total energy intake
							Buttermilk (200 g/d)		1.04 (0.97; 1.10)	
								Whole-fat milk (200 g/d)	1.07 (1.00; 1.14)	
							Low-fat yogurt products (200 g/d)	Low-fat milk (200 g/d)	0.95 (0.88; 1.03)	
								Whole-fat milk (200 g/d)	0.98 (0.90; 1.06)	
								Buttermilk (200 g/d)	0.92 (0.83; 1.01)	
							Whole-fat yogurt products (200 g/d)	Low-fat milk (200 g/d)	1.03 (0.96; 1.11)	
								Whole-fat milk (200 g/d)	1.06 (0.98; 1.15)	
								Buttermilk (200 g/d)	1.00 (0.91; 1.10)	

								Low-fat yogurt products (200 g/d)	1.08 (0.98; 1.21)	
							Cheese (20 g/d)	Low-fat milk (200 g/d)	1.04 (1.01; 1.06)	
								Whole-fat milk (200 g/d)	1.07 (1.03; 1.11)	
								Buttermilk (200 g/d)	1.00 (0.94; 1.06)	
								Low-fat yogurt products (200 g/d)	1.09 (1.01; 1.18)	
								Whole-fat yogurt products (200 g/d)	1.02 (0.95; 1.10)	
			1491	CVD mortality			Whole-fat milk (200 g/d)	Low-fat milk (200 g/d)	0.97 (0.91; 1.04)	
							Buttermilk (200 g/d)		1.11 (0.96; 1.27)	
								Whole-fat milk (200 g/d)	1.14 (0.98; 1.32)	
							Low-fat yogurt products (200 g/d)	Low-fat milk (200 g/d)	0.95 (0.80; 1.12)	
								Whole-fat milk (200 g/d)	0.98 (0.82; 1.15)	
								Buttermilk (200 g/d)	0.86 (0.69; 1.06)	
							Whole-fat yogurt products (200 g/d)	Low-fat milk (200 g/d)	1.04 (0.89; 1.22)	
								Whole-fat milk (200 g/d)	1.07 (0.90; 1.27)	
								Buttermilk (200 g/d)	0.94 (0.76; 1.17)	
								Low-fat yogurt products (200 g/d)	1.1 (0.88; 1.38)	

							Cheese (20 g/d)	Low-fat milk (200 g/d)	1.07 (1.02; 1.13)	
								Whole-fat milk (200 g/d)	1.10 (1.02; 1.19)	
								Buttermilk (200 g/d)	0.97 (0.84; 1.11)	
								Low-fat yogurt products (200 g/d)	1.13 (0.96; 1.33)	
								Whole-fat yogurt products (200 g/d)	1.03 (0.87; 1.21)	
		29161, W, 56.3 years	4774	All-cause mortality			Whole-fat milk (200 g/d)	Low-fat milk (200 g/d)	0.93 (0.88; 0.97)	
							Buttermilk (200 g/d)		1.00 (0.95; 1.06)	
								Whole-fat milk (200 g/d)	1.08 (1.01; 1.16)	
							Low-fat yogurt products (200 g/d)	Low-fat milk (200 g/d)	0.95 (0.88; 1.02)	
								Whole-fat milk (200 g/d)	1.02 (0.94; 1.11)	
								Buttermilk (200 g/d)	0.94 (0.86; 1.03)	
							Whole-fat yogurt products (200 g/d)	Low-fat milk (200 g/d)	0.97 (0.90; 1.06)	
								Whole-fat milk (200 g/d)	1.05 (0.96; 1.15)	
								Buttermilk (200 g/d)	0.97 (0.88; 1.07)	
								Low-fat yogurt products (200 g/d)	1.03 (0.93; 1.14)	
							Cheese (20 g/d)	Low-fat milk (200 g/d)	1.04 (1.01; 1.08)	

								Whole-fat milk (200 g/d)	1.12 (1.07; 1.18)	
								Buttermilk (200 g/d)	1.04 (0.98; 1.10)	
								Low-fat yogurt products (200 g/d)	1.1 (1.02; 1.19)	
								Whole-fat yogurt products (200 g/d)	1.07 (0.99; 1.16)	
			668	CVD mortality			Whole-fat milk (200 g/d)	Low-fat milk (200 g/d)	0.95 (0.83; 1.08)	
							Buttermilk (200 g/d)	Low-fat milk (200 g/d)	0.96 (0.84; 1.10)	
								Whole-fat milk (200 g/d)	1.01 (0.85; 1.20)	
							Low-fat yogurt products (200 g/d)	Low-fat milk (200 g/d)	1.01 (0.83; 1.23)	
								Whole-fat milk (200 g/d)	1.07 (0.86; 1.32)	
								Buttermilk (200 g/d)	1.06 (0.84; 1.33)	
							Whole-fat yogurt products (200 g/d)	Low-fat milk (200 g/d)	0.94 (0.76; 1.16)	
								Whole-fat milk (200 g/d)	0.99 (0.78; 1.26)	
								Buttermilk (200 g/d)	0.98 (0.77; 1.26)	
								Low-fat yogurt products (200 g/d)	0.93 (0.71; 1.21)	
							Cheese (20 g/d)	Low-fat milk (200 g/d)	1.06 (0.98; 1.15)	
								Whole-fat milk (200 g/d)	1.12 (0.98; 1.28)	
								Buttermilk (200 g/d)	1.11 (0.96, 1.28)	

								Low-fat yogurt products (200 g/d)	1.05 (0.86; 1.27)	
								Whole-fat yogurt products (200 g/d)	1.13 (0.91; 1.39)	
Li 2022, UKB, United Kingdom (72)	11.4	34616, M/W, 58 years	663	T2D incidence	Cumulative medical records of hospital diagnoses	Validated FFQ (baseline assessment)	Red meat (50 g/d)	Yogurt (70 g/d)	0.87 (0.77; 0.97)	Age, sex, residence area, Townsend deprivation index at recruitment, BMI, WHR, smoking status, alcohol intake, physical activity, antihypertensive medication treatment, family history of diabetes, multivitamin use, other protein sources, total energy intake
								Milk (200 g/d)	1.10 (0.93; 1.29)	
								Cheese (30 g/d)	0.83 (0.70; 0.98)	
							Processed meat (50 g/d)	Yogurt (70 g/d)	0.84 (0.70; 1.01)	
								Milk (200 g/d)	1.07 (0.86; 1.32)	
								Cheese (30 g/d)	0.81 (0.65; 1.00)	
							Eggs (50 g/d)	Yogurt (70 g/d)	0.90 (0.78; 1.04)	
								Milk (200 g/d)	1.15 (0.96; 1.37)	
								Cheese (30 g/d)	0.87 (0.71; 1.06)	
							Poultry (50 g/d)	Yogurt (70 g/d)	0.88 (0.78; 0.99)	
								Milk (200 g/d)	1.12 (0.95; 1.31)	
								Cheese (30 g/d)	0.84 (0.71; 1.00)	
Li 2022, WHI, USA (72)	15.8	108681, W, 63 years	15842	T2D incidence	Self-report via follow-up validated questionnaires	Validated FFQ (baseline assessment)	Red meat (50 g/d)	Yogurt (70 g/d)	0.96 (0.93; 0.99)	Age, race, region of residence, family income, education, study group indicator, BMI, WHR, smoking status, alcohol intake, physical activity, antihypertensive medication use, family history of diabetes, hormone replacement therapy, multivitamin use, modified AHEI-2010 score, intake of other protein sources, total energy intake
								Milk (200 g/d)	0.96 (0.93; 0.98)	
								Cheese (30 g/d)	0.96 (0.94; 0.98)	
							Processed meat (50 g/d)	Yogurt (70 g/d)	0.89 (0.84; 0.94)	
								Milk (200 g/d)	0.89 (0.84; 0.94)	
								Cheese (30 g/d)	0.89 (0.84; 0.94)	
							Eggs (50 g/d)	Yogurt (70 g/d)	0.88 (0.84; 0.92)	
								Milk (200 g/d)	0.88 (0.84; 0.91)	
								Cheese (30 g/d)	0.88 (0.84; 0.91)	
							Poultry (50 g/d)	Yogurt (70 g/d)	0.97 (0.93, 1.01)	
								Milk (200 g/d)	0.96 (0.93, 1.00)	
								Cheese (30 g/d)	0.96 (0.93, 1.00)	

Lim 2022, SMEC, Asia (73)	10.1	12408, M/W, 45.6 years	746	CVD incidence/ mortality	Linkage with National Registry of Diseases Office, validated by registry staff through review of medical records	Interviewer-administered, validated, semi-quantitative FFQ (baseline assessment)	Refined grains (1 serving/d) (baseline assessment)	Dairy (1 serving/d)	0.90 (0.82; 0.98)	Age, sex, ethnicity, educational level, BMI, smoking, alcohol consumption, physical activity, history of diabetes, hypertension, dyslipidemia, family history of heart disease, menopausal status, intakes of fiber, cholesterol, other macronutrients except for carbohydrates, total energy intake
Liu 2017, WHI, USA (74)	13.2	69223, W, 63.1 years	4229	CAD incidence/ mortality	Medical record review or self-reported hospitalizations validated by trained physician adjudicators	FFQ (calculated cumulative mean intake based on multiple assessments)	Butter (1 teaspoon)	Stick margarine (1 teaspoon)	1.00 (0.99; 1.10)	Age, region, race/ethnicity, income, BMI, smoking status, physical activity, hypertension, family history of MI, postmenopausal HRT, aspirin use, hysterectomy, total energy intake
								Tub margarine (1 teaspoon)	1.00 (0.97; 1.10)	
								Low-fat margarine (1 teaspoon)	1.00 (0.97; 1.10)	
								Margarine (1 teaspoon)	1.10 (1.00; 1.10)	
			5507	CVD incidence/ mortality				Stick margarine (1 teaspoon)	1.00 (0.99; 1.10)	
								Tub margarine (1 teaspoon)	1.00 (0.97; 1.00)	
								Low-fat margarine (1 teaspoon)	1.00 (0.97; 1.10)	
								Margarine (1 teaspoon)	1.10 (1.00; 1.10)	
			1550					Stick margarine (1 teaspoon)	1.10 (0.97; 1.10)	

				Stroke incidence/ mortality				Tub margarine (1 teaspoon)	1.00 (0.93; 1.10)	
								Low-fat margarine (1 teaspoon)	1.00 (0.93; 1.10)	
								Margarine (1 teaspoon)	1.10 (0.97; 1.20)	
Lyskjær 2020, DCH, Denmark (75)	13.4	55095, M/W, 56.1 years	2260	Stroke incidence/ mortality	Linkage to Danish National Patient Register validated by a physician with neurological experience	Validated FFQ (baseline assessment)	Yogurt (200 g/d)	Oatmeal (50 g/d)	0.98 (0.96; 1.01)	Age, sex, educational level, calendar time, smoking, alcohol, physical activity, BMI, history of diabetes, history of hypercholesterolemia, history of hypertension, modified Alternative Healthy Eating Index
Malik 2016, NHS, NHS II, HPFS, USA (76)	20	205802, M/W, 53 years (HPFS), 50 years (NHS), 36 years (NHS II)	15580	T2D incidence	Self-report, validated via supplementary questionnaire (symptoms, diagnostic tests, treatment), confirmed according to criteria of National Diabetes Association	Validated FFQ (calculated cumulative mean intake based on multiple assessments)	Dairy (240 ml/d)	Peanuts and peanut butter (28 g/d)	0.98 (0.93; 1.03)	Age, ethnicity, smoking status, alcohol intake, physical activity, BMI, family history of diabetes, postmenopausal HRT (NHS, NHS II), oral contraceptive use (NHS II), intakes of sugar-sweetened beverages, fruit, vegetables, mutually adjusted for other food sources of animal protein, refined grains, potatoes
								Whole grains (32 g/d bread or 200 g/d cooked brown rice or cereals)	0.97 (0.95; 1.00)	

Pacheco 2022, NHS, HPFS, USA (77)	30 (13.3* (M), 14.2* (W))	110487, M/W, 52.6 years	9185	CAD incidence/ mortality	Self-report validated by medical records, National Death Index, report by family members, autopsy reports, death certificates	Validated, semi-quantitative FFQ (simple update approach for dietary variables)	Butter (40 g/d)	Avocado (40 g/d)	0.69 (0.63; 0.9)	Age, race, ancestry, BMI, smoking status, alcohol intake, physical activity, diabetes, hypertension or antihypertensive medication use, hypercholesterolemia or cholesterol-lowering medication use, aspirin use, family history of diabetes, family history of MI, family history of cancer, postmenopausal status and menopausal HRT, multivitamin use, intake of red and processed meat, fruits, vegetables, nuts, soda, whole grains, eggs, tortilla, breads, cheese, trans-fat, mutually adjusted for other types of fat-containing food, total energy intake
							Yogurt (40 g/d)		0.79 (0.69; 0.9)	
							Cheese (40 g/d)		0.75 (0.65; 0.87)	
							Dairy foods (milk, cheese, yogurt) (40 g/d)		0.84 (0.7; 1.01)	
			14274	CVD incidence/ mortality			Butter (40 g/d)		0.78 (0.63; 0.96)	
							Yogurt (40 g/d)		0.89 (0.80; 0.99)	
							Cheese (40 g/d)		0.87 (0.77; 0.98)	
							Dairy foods (milk, cheese, yogurt) (40 g/d)		0.91 (0.79; 1.05)	
			5390	Stroke incidence/ mortality			Butter (40 g/d)		0.89 (0.63; 1.25)	
							Yogurt (40 g/d)		1.05 (0.91; 1.22)	
							Cheese (40 g/d)		1.07 (0.90; 1.28)	
							Dairy foods (milk, cheese, yogurt) (40 g/d)		0.98 (0.79; 1.23)	
Pan 2011, HPFS, NHS,	16-28	204157, M/W, 35.9-53.6 years	13759	T2D incidence	Self-report and reconfirmation via medical records (in	Validated, semi-quantitative	Unprocessed red meat (85 g/d)	Low-fat dairy (1 serving/d: 240 ml milk, 28 g	0.84 (0.81; 0.87)	Age, race, BMI, smoking status, alcohol intake,

NHS II, USA (78)					the HPFS and NHS)	FFQ (calculated cumulative mean intake based on multiple assessments)	Processed red meat (50 g/d)	cheese, or 120 ml yogurt)	0.71 (0.67; 0.75)	physical activity level, family history of diabetes, history of hypertension and hypercholesterolemia, menopausal status and HRT in women, dietary score, energy intake
							Total red meat (140 g/d)		0.83 (0.80; 0.86)	
Pan 2012a, HPFS, NHS, USA (79)	22 (HPFS), 28 (NHS)	121342, M/W, 53 years (HPFS), 46 years (NHS)	23926	All-cause mortality	State vital records, National Death Index, next of kin or postal system, medical records	Validated, semi-quantitative FFQ (calculated cumulative mean intake based on multiple assessments)	Unprocessed red meat (85 g/d)	Low-fat dairy (1 serving/d)	0.91 (0.85; 0.99)	Age, race, BMI, alcohol, smoking, physical activity, family history of diabetes, MI or cancer, history of diabetes, hypertension, or hypercholesterolemia, menopausal status and HRT, corresponding two dietary variables, total energy intake
							Processed red meat (85 g/d)		0.84 (0.78; 0.90)	
							Total red meat (142 g/d)		0.90 (0.86; 0.94)	
Pan 2012b, NHS II, USA (80)	18	82902, W, 36 years	2718	T2D incidence	Self-report, validated via supplementary questionnaire (symptoms, diagnostic tests, treatment)	Validated FFQ (calculated cumulative mean intake based on multiple assessments)	Sugar-sweetened beverage (330 ml/d)	Milk (240 ml/d)	0.88 (0.83; 0.93)	Age, race, BMI, smoking status, alcohol intake, physical activity, family history of diabetes, menopausal status and HRT, oral contraceptive use, Alternative Healthy Eating Index
							Fruit juice (120 ml/d)		0.85 (0.79; 0.92)	
	26	40278, M, 63 years	12397	All-cause mortality	State vital records, National Death	Validated, semi-	Yogurt (245 g/d)	Red meat (85 g/d)	1.16 (1.05; 1.29)	Age, ethnicity, height, 2-y follow-up cycle,

Schmid 2020, HPFS, USA (81)					Index, next of kin or postal system, medical records	quantitative FFQ (calculated cumulative mean intake based on multiple assessments)		Processed meat (37 g/d)	1.16 (1.04; 1.28)	BMI, BMI at age 21y, smoking status, pack-years of smoking, alcohol consumption, physical activity, history of hypertension, history of hypercholesterolemia, history of diabetes, family history of cancer, family history of diabetes, family history of MI, current multivitamin use, regular aspirin use, total caloric intake
								Nuts (28 g/d)	0.86 (0.78; 0.95)	
								Whole grains (28 g/d)	0.93 (0.84; 1.02)	
								Other dairy foods (1 serving/d)	1.05 (0.95; 1.16)	
								Milk (227 g/d)	1.06 (0.95; 1.17)	
								Cheese (28 g/d)	1.07 (0.97; 1.18)	
Schmid, 2020, NHS, USA (76)	32	82348, W, 65 years	20831	All-cause mortality	State vital records, National Death Index, next of kin or postal system, medical records	Validated, semi-quantitative FFQ (calculated cumulative mean intake based on multiple assessments)	Yogurt (245 g/d)	Red meat (85 g/d)	1.14 (1.05, 1.24)	Age, ethnicity, height, 2-y follow-up cycle, BMI, BMI at age 21y, smoking status, pack-years of smoking, alcohol consumption, physical activity, history of hypertension, history of hypercholesterolemia, history of diabetes, family history of cancer, family history of diabetes, family history of MI, current multivitamin use, regular aspirin use, total caloric intake
								Processed meat (37 g/d)	1.31 (1.20, 1.43)	
								Nuts (28 g/d)	0.73 (0.67, 0.79)	
								Whole grains (28 g/d)	0.86 (0.79, 0.94)	
								Other dairy foods (1 serving/d)	1.08 (0.99, 1.17)	
								Milk (227 g/d)	1.15 (1.05, 1.25)	
								Cheese (28 g/d)	1.01 (0.93, 1.10)	

Stuber, 2021, EPIC-NL, Netherlands (82)	15	35982, M/W, 48-50 years	1467	T2D incidence	Self-report via follow-up questionnaires and/or linkage to hospital charge diagnosis registry validated by consulting the general practitioner or pharmacist	Validated FFQ (baseline assessment)	Whole-fat milk (200 g/d)	Skimmed milk (200 g/d)	1.01 (0.83; 1.23)	Age, sex, education level, smoking status, alcohol intake, physical activity, hypertension, dietary intake of fruits, vegetables, processed meat, red meat, coffee, sugar-sweetened beverages, energy adjusted fiber, total energy intake
								Buttermilk (200 g/d)	1.03 (0.87; 1.22)	
							Buttermilk (200 g/d)	Skimmed fermented milk (200 g/d)	0.93 (0.76; 1.15)	
								Whole-fat yogurt (200 g/d)	1.17 (0.74; 1.84)	
							Skimmed fermented milk (200 g/d)	Skimmed milk (200 g/d)	1.08 (0.93; 1.27)	
								Whole-fat yogurt (200 g/d)	1.25 (0.8; 1.98)	
								Skimmed fermented milk (200 g/d)	0.91 (0.78; 1.06)	
								Whole-fat yogurt (200 g/d)	1.14 (0.74; 1.75)	
							Skimmed milk (200 g/d)	Whole-fat yogurt (200 g/d)	1.01 (0.66; 1.56)	
								Buttermilk (200 g/d)	1.02 (0.92; 1.12)	
Sun 2021, WHI, USA (83)	18.1	102521, W, 63 years	25976	All-cause mortality	Death certificates, medical records, autopsy reports, linkage to National Death Index	Validated FFQ (baseline assessment)	Total red meat (57 g/d)	Dairy (57 g/d)	1.00 (0.98; 1.03)	Age, race/ethnicity, socioeconomic status, BMI, lifestyle, baseline health status, family history of heart attack/stroke, HRT history, whole grain consumption, vegetable consumption, fruit consumption, sugar-sweetened beverage consumption, and mutual adjustment for
							Dairy (57 g/d)	Nuts (57 g/d)	0.94 (0.89; 0.99)	
			6993	CVD mortality			Total red meat (57 g/d)	Dairy (57 g/d)	1.03 (0.98; 1.09)	
							Dairy (57 g/d)	Nuts (57 g/d)	0.90 (0.82; 0.99)	

										other protein sources, total energy intake
van den Brandt 2019, NLCS, Netherlands (84)	10	12025, M/W, 61.3 years	8823	All-cause mortality	Linkage with Dutch Central Bureau of Genealogy and Statistics Netherlands	Validated FFQ (baseline assessment)	Processed meat (50 g/d)	Low-fat dairy (50 g/d)	0.90 (0.76; 1.08)	Age, education, body height, BMI, smoking status, number of cigarettes smoked per day, years of smoking, alcohol intake, physical activity, history of hypertension, history of diabetes, postmenopausal HRT, intake of vegetables, fruit, use of nutritional supplements, total energy intake
			2985	CVD mortality					0.81 (0.66; 1.00)	
Virtanen 2017, KIHDRF, Finland (85)	19.3	2332, M, 42-60 years	432	T2D incidence	2h oral glucose tolerance test at re-examinations and information from national hospital discharge registry and Social Insurance Institution of Finland register	4d dietary records (baseline assessment)	Dairy (50 g/d)	Egg (50 g/d)	0.73 (0.59; 0.92)	Age, education, examination year, income, pack-years of smoking, alcohol intake physical activity, use of hypertension medication, family history of diabetes, serum ferritin, marital status, glycemic index, intakes of fiber, Mg, coffee, cholesterol, SFA, MUFA, PUFA, trans-fatty acids, energy intake
							Non-fermented dairy (50 g/d)		0.74 (0.59; 0.92)	
							Fermented dairy (50 g/d)		0.73 (0.59; 0.91)	
Zhang 2021, NIH-AARP	16	521120, M/W, 62 years	129328	All-cause mortality	Annual linkage to Social Security	Validated FFQ	Butter (8 g/d)	Margarine (8 g/d)	0.97 (0.96; 0.98)	Age, sex, race, education, marital

Diet and Health Study, USA (86)					Administration Death Master File validated by follow-up searches of National Death Index Plus	(baseline assessment)		Corn oil (8 g/d)	0.95 (0.93; 0.98)	status, household income, BMI, smoking, alcohol, vigorous physical activity, usual activity at work, perceived health condition, history of heart disease, stroke, diabetes, cancer at baseline, Healthy Eating Index-2015, total energy intake
								Canola oil (8 g/d)	0.94 (0.92; 0.96)	
								Olive oil (8 g/d)	0.93 (0.91; 0.96)	
			38747	CVD mortality				Margarine (8 g/d)	0.99 (0.97; 1.02)	
								Corn oil (8 g/d)	0.96 (0.92; 1.00)	
								Canola oil (8 g/d)	0.93 (0.89; 0.97)	
								Olive oil (8 g/d)	0.93 (0.89; 0.97)	
			3512	Diabetes mortality				Margarine (8 g/d)	0.95 (0.88; 1.02)	
								Corn oil (8 g/d)	0.88 (0.77; 1.02)	
								Canola oil (8 g/d)	0.98 (0.86; 1.11)	
								Olive oil (8 g/d)	0.85 (0.73; 0.98)	
Zhuang 2020, CHNS, China (87)	15	18914, M/W, 43 years	1429	All-cause mortality	Report from household members	3 consecutive 24-h recalls, changes in the home food inventory (calculated cumulative mean intake based on multiple assessments)	Eggs (50 g/d or 1 egg/d)	Dairy (50 g/d)	0.91 (0.84; 0.98)	Age, sex, nationality, marital status, household income, urbanization index, education, BMI, smoking, alcohol intake, physical activity, history of CVD, cancer, diabetes, hypertension, consumption of cereals, potatoes, dairy products, nuts/legumes, red meat, white meat, sugar-sweetened beverages, vegetables, fruit, total energy intake

Zhuang 2021, NIH-AARP Diet and Health Study, USA (88)	16	521120, M/W, 62 years	129328	All-cause mortality	Linkage to National Death Index Plus	Validated FFQ (baseline assessment)	Eggs (1 egg/d, 25g)	Dairy (25 g/d)	0.93 (0.93; 0.94)	Age, sex, race, education, marital status, household income, BMI, smoking, alcohol, physical activity, history of hypertension, hypercholesteremia, heart disease, stroke, diabetes, cancer at baseline, intakes of fruit, vegetables, potatoes, whole grains, refined grains, coffee, sugar-sweetened beverages, total energy intake
			38747	CVD mortality					0.93 (0.92; 0.94)	
			3512	Diabetes mortality					0.93 (0.89; 0.97)	

Abbreviations: AHEI Alternative healthy Eating Index; AHS-2 Adventist Health Study-2; ARIC Atherosclerosis Risk in Communities; ATBC Alpha-Tocophenol and Beta-Carotene Cancer Prevention Study; BMI body mass index; CAD coronary artery disease; CHNS China Health and Nutrition Survey; CI confidence interval; CVD cardiovascular disease; d day; DCH Danish Diet, Cancer and Health cohort; EPIC European Prospective Investigation into Cancer and Nutrition; FFQ food frequency questionnaire; HDL-C high-density lipoprotein cholesterol; HPFS Health Professionals Follow-Up Study; HPP Harvard Pooling Project; HRT hormone replacement therapy; IWHS The Iowa Women's Health Study; KIHDRF Kuopio Ischaemic Heart Disease Risk Factor Study; M men; Mg magnesium; MI myocardial infarction; MUFA monosaturated fatty acid; NIH-AARP National Institutes of Health - American Association of Retired Persons Diet and Health Study; NHS Nurses Health Study; NHS II Nurses Health Study II; NL Netherlands; NLCS Netherlands Cohort Study; PUFA poly saturated fatty acid; RR relative risk; SFA saturated fatty acid; SMEC Singapore Multi-Ethnic Cohort; T2D type 2 diabetes; UKB United Kingdom Biobank; W women; WHI Women’s Health Initiative; WHR waist-to-hip ratio; WHS The Women's Health Study; *values represent median

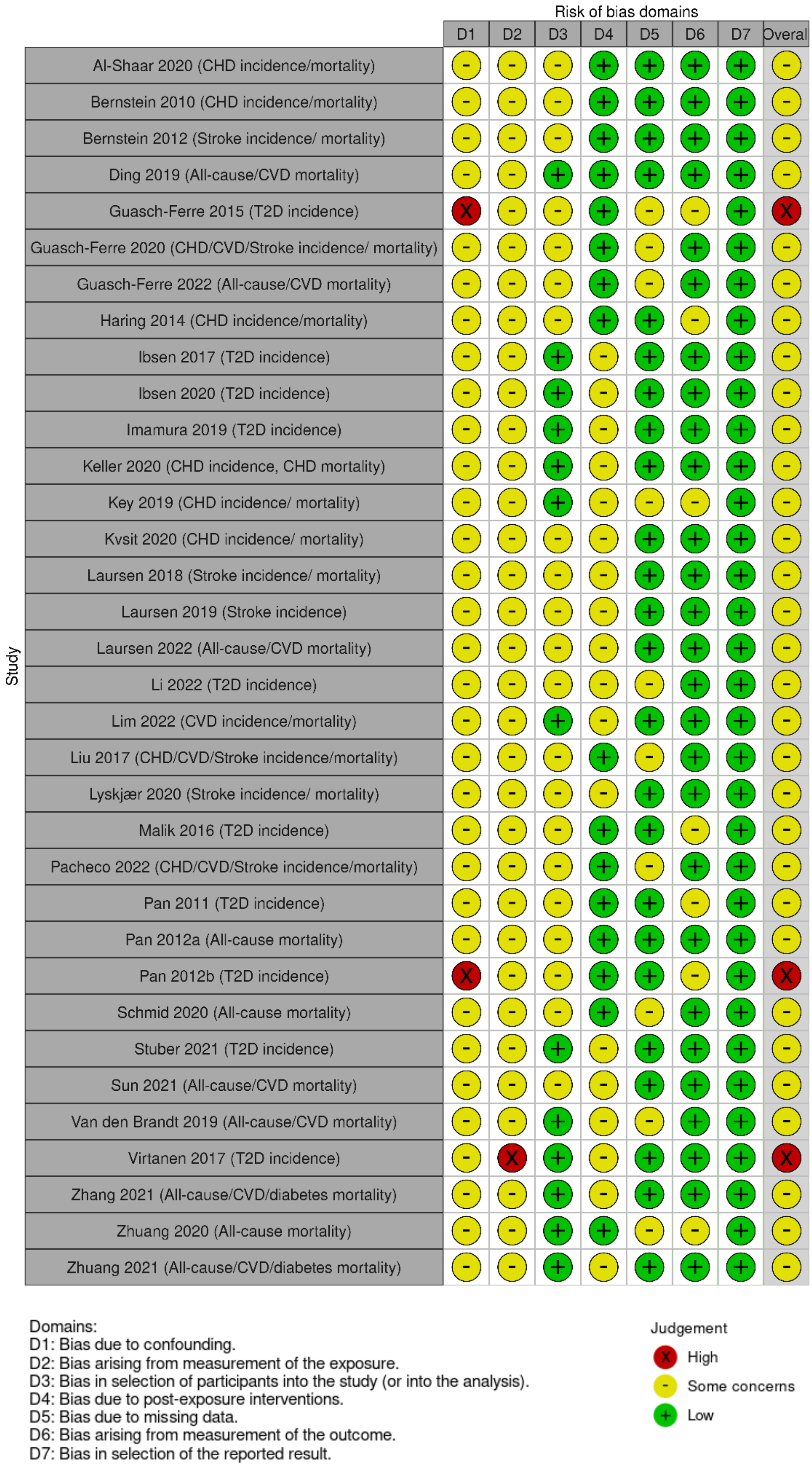

Supplementary figure 1: Risk of bias of each study for each domain and overall

T2D type 2 diabetes; CHD coronary heart disease (=coronary artery disease); CVD cardiovascular disease

Supplementary table 5: GRADE assessment for the substitution analyses regarding all-cause mortality

N of studies	Study design	Risk of bias	Inconsistency	Indirectness	Imprecision	Other considerations	Number of cases/participants	Relative effect (95% CI)	Absolute effect (95% CI)	Certainty
**Yogurt substituted with cheese**										
2	observational studies	serious[a]	not serious	not serious	serious[b]	none	33228/122626 (27.1%)	**RR 1.03** (0.98 to 1.09)	**8 more per 1.000** (from 5 fewer to 24 more)	⊕⊕◯◯ Low
**Yogurt substituted with milk**										
2	observational studies	serious[a]	not serious	not serious	not serious	none	33228/122626 (27.1%)	**RR 1.09** (1.02 to 1.16)	**24 more per 1.000** (from 5 more to 43 more)	⊕⊕⊕◯ Moderate
**Yogurt substituted with other dairy**										
2	observational studies	serious[a]	not serious	not serious	serious[b]	none	33228/122626 (27.1%)	**RR 1.05** (1.00 to 1.11)	**14 more per 1.000** (from 0 fewer to 30 more)	⊕⊕◯◯ Low

N of studies	Study design	Risk of bias	Inconsistency	Indirectness	Imprecision	Other considerations	Number of cases/participants	Relative effect (95% CI)	Absolute effect (95% CI)	Certainty
**Dairy substituted with eggs**										
2	observational studies	serious[a]	serious[c]	not serious	not serious	none	130757/540034 (24.2%)	**RR 1.65** (1.31 to 2.08)	**157 more per 1.000** (from 75 more to 261 more)	⊕⊕◯◯ Low
**Butter substituted with olive oil**										
3	observational studies	serious[a]	not serious[d]	not serious	not serious	none	166184/613503 (27.1%)	**RR 0.94** (0.92 to 0.97)	**16 fewer per 1.000** (from 22 fewer to 8 fewer)	⊕⊕⊕◯ Moderate
**Yogurt substituted with red meat**										
2	observational studies	serious[a]	not serious	not serious	not serious	none	33228/122626 (27.1%)	**RR 1.12** (1.06 to 1.18)	**33 more per 1.000** (from 16 more to 49 more)	⊕⊕⊕◯ Moderate
**Yogurt substituted with processed meat**										

N of studies	Study design	Risk of bias	Inconsistency	Indirectness	Imprecision	Other considerations	Number of cases/participants	Relative effect (95% CI)	Absolute effect (95% CI)	Certainty
2	observational studies	serious[a]	not serious	not serious	not serious	none	33228/122626 (27.1%)	**RR 1.19** (1.08 to 1.32)	**51 more per 1.000** (from 22 more to 87 more)	⨁⨁⨁◯ Moderate

Yogurt substituted with nuts

2	observational studies	serious[a]	not serious[d]	not serious	not serious	none	33228/122626 (27.1%)	**RR 0.82** (0.72 to 0.94)	**49 fewer per 1.000** (from 76 fewer to 16 fewer)	⨁⨁⨁◯ Moderate

Yogurt substituted with whole grains

2	observational studies	serious[a]	not serious	not serious	not serious	none	33228/122626 (27.1%)	**RR 0.91** (0.85 to 0.97)	**24 fewer per 1.000** (from 41 fewer to 8 fewer)	⨁⨁⨁◯ Moderate

Dairy substituted with red and processed meat

N of studies	Study design	Risk of bias	Inconsistency	Indirectness	Imprecision	Other considerations	Number of cases/participants	Relative effect (95% CI)	Absolute effect (95% CI)	Certainty
3	observational studies	serious[a]	not serious[e]	not serious	serious[b]	none	51438/217755 (23.6%)	**RR 1.05** (1.01 to 1.09)	**12 more per 1.000** (from 2 more to 21 more)	⊕⊕◯◯ Low
**Dairy substituted with poultry**										
3	observational studies	serious[a]	not serious[e]	not serious	serious[b]	none	51438/217755 (23.6%)	**RR 0.93** (0.85 to 1.03)	**17 fewer per 1.000** (from 35 fewer to 7 more)	⊕⊕◯◯ Low
**Dairy substituted with nuts and legumes**										
3	observational studies	serious[a]	not serious[e]	serious[f]	not serious	none	51438/217755 (23.6%)	**RR 0.86** (0.78 to 0.95)	**33 fewer per 1.000** (from 52 fewer to 12 fewer)	⊕⊕◯◯ Low
**Dairy substituted with whole grains**										

N of studies	Study design	Risk of bias	Inconsistency	Indirectness	Imprecision	Other considerations	Number of cases/participants	Relative effect (95% CI)	Absolute effect (95% CI)	Certainty
3	observational studies	serious[a]	not serious[e]	not serious	not serious	none	51438/217755 (23.6%)	**RR 0.89** (0.84 to 0.93)	**26 fewer per 1.000** (from 38 fewer to 17 fewer)	⊕⊕⊕◯ Moderate
**Low fat dairy substituted with red meat**										
2	observational studies	serious[a]	not serious[e]	not serious	not serious	none	23926/121342 (19.7%)	**RR 1.11** (1.06 to 1.16)	**22 more per 1.000** (from 12 more to 32 more)	⊕⊕⊕◯ Moderate
**Low fat dairy substituted with processed red meat**										
2	observational studies	serious[a]	not serious[e]	not serious	not serious	none	23926/121342 (19.7%)	**RR 1.19** (1.11 to 1.28)	**37 more per 1.000** (from 22 more to 55 more)	⊕⊕⊕◯ Moderate
**Low fat dairy substituted with unprocessed red meat**										

N of studies	Study design	Risk of bias	Inconsistency	Indirectness	Imprecision	Other considerations	Number of cases/participants	Relative effect (95% CI)	Absolute effect (95% CI)	Certainty
2	observational studies	serious[a]	not serious[e]	not serious	serious[b]	none	23926/121342 (19.7%)	**RR 1.10** (1.02 to 1.19)	**20 more per 1.000** (from 4 more to 37 more)	⊕⊕◯◯ Low

CI: confidence interval; **RR:** risk ratio

Explanations

a. Downgraded by one level for risk of bias, since all studies were rated with a moderate risk of bias.

b. Downgraded by one level for imprecision, since 95% CI crosses threshold of a minimal important difference (absolute risk: 5 fewer and/or 5 more events per 1.000).

c. Downgraded by one level for inconsistency, since point estimates and 95% CI did not fully overlap between studies and I^2 was substantial.

d. Not downgraded for inconsistency, although 95% CI did not overlap between studies and I^2 was very high, but point estimates are in agreement and 95% are very narrow.

e. Although RR and 95% CI of the individual cohort studies are not available (only pooled results are available), and therefore we were unable to calculate I^2 and tau, we did not assume statistical and clinical heterogeneity, due to the homogeneity in the design of the Harvard cohort studies.

f. Downgraded by one level for indirectness, since the food category was "nuts and legumes", as the health association is difficult to assign to one these two foods.

Supplementary table 6: GRADE assessment for the substitution analyses regarding fatal and non-fatal cardiovascular disease

N of studies	Study design	Risk of bias	Inconsistency	Indirectness	Imprecision	Other considerations	Number of cases/participants	Relative effect (95% CI)	Absolute effect (95% CI)	Certainty
**Butter substituted with olive oil**										
2	observational studies	serious[a]	not serious	not serious	serious[b]	none	9797/92978 (10.5%)	**RR 0.95** (0.91 to 1.00)	**5 fewer per 1.000** (from 9 fewer to 0 fewer)	⊕⊕◯◯ Low
**Dairy substituted with avocado**										
2	observational studies	serious[a]	not serious	not serious	serious[b]	none	14274/110487 (12.9%)	**RR 0.62** (0.31 to 1.28)	**49 fewer per 1.000** (from 89 fewer to 36 more)	⊕⊕◯◯ Low
**Yogurt substituted with avocado**										
2	observational studies	serious[a]	not serious	not serious	serious[c]	none	14274/110487 (12.9%)	**RR 0.56** (0.33 to 0.95)	**57 fewer per 1.000** (from 87 fewer to 6 fewer)	⊕⊕◯◯ Low

N of studies	Study design	Risk of bias	Inconsistency	Indirectness	Imprecision	Other considerations	Number of cases/participants	Relative effect (95% CI)	Absolute effect (95% CI)	Certainty
**Cheese substituted with avocado**										
2	observational studies	serious[a]	not serious	not serious	serious[b]	none	14274/110487 (12.9%)	**RR 0.90** (0.82 to 0.98)	**13 fewer per 1.000** (from 23 fewer to 3 fewer)	⨁⨁◯◯ Low
**Butter substituted with avocado**										
2	observational studies	serious[a]	not serious	not serious	serious[b]	none	14274/110487 (12.9%)	**RR 0.97** (0.94 to 0.99)	**4 fewer per 1.000** (from 8 fewer to 1 fewer)	⨁⨁◯◯ Low

CI: confidence interval; **RR:** risk ratio

Explanations

a. Downgraded by one level for risk of bias, since all studies were rated with a moderate risk of bias.

b. Downgraded by one level for imprecision, since 95% CI crosses threshold of a minimal important difference (absolute risk: 5 fewer and/or 5 more events per 1.000).

c. Downgraded by one level for imprecision, since the 95% CI did not cross the threshold of a minimal important difference (absolute risk: 5 fewer and/or 5 more events per 1.000) but the relative effect was large (RR<0.7), and the ratio of the upper to the lower boundary of the 95% CI was nearly three (2.9).

Supplementary table 7: GRADE assessment for the substitution analyses regarding cardiovascular disease mortality

N of studies	Study design	Risk of bias	Inconsistency	Indirectness	Imprecision	Other considerations	Number of cases/participants	Relative effect (95% CI)	Absolute effect (95% CI)	Certainty
**Butter substituted with olive oil**										
3	observational studies	serious[a]	not serious	not serious	not serious	none	47568/613503 (7.8%)	**RR 0.96** (0.94 to 0.98)	**3 fewer per 1.000** (from 5 fewer to 2 fewer)	⊕⊕⊕◯ Moderate
**Dairy substituted with red and processed meat**										
3	observational studies	serious[a]	not serious[b]	not serious	serious[c]	none	12143/217755 (5.6%)	**RR 1.08** (1.02 to 1.15)	**4 more per 1.000** (from 1 more to 8 more)	⊕⊕◯◯ Low
**Dairy substituted with poultry**										
3	observational studies	serious[a]	not serious[b]	not serious	serious[c]	none	12143/217755 (5.6%)	**RR 1.01** (0.84 to 1.20)	**1 more per 1.000** (from 9 fewer to 11 more)	⊕⊕◯◯ Low

N of studies	Study design	Risk of bias	Inconsistency	Indirectness	Imprecision	Other considerations	Number of cases/participants	Relative effect (95% CI)	Absolute effect (95% CI)	Certainty
**Dairy substituted with nuts and legumes**										
3	observational studies	serious[a]	not serious[b]	serious[d]	serious[c]	none	12143/217755 (5.6%)	**RR 0.89** (0.84 to 0.93)	**6 fewer per 1.000** (from 9 fewer to 4 fewer)	⨁◯◯◯ Very low
**Dairy substituted with whole grains**										
3	observational studies	serious[a]	not serious[b]	not serious	serious[c]	none	12143/217755 (5.6%)	**RR 0.93** (0.85 to 1.01)	**4 fewer per 1.000** (from 8 fewer to 1 more)	⨁⨁◯◯ Low

CI: confidence interval; **RR:** risk ratio

Explanations

a. Downgraded by one level for risk of bias, since all studies were rated with a moderate risk of bias.

b. Although RR and 95% CI of the individual cohort studies are not available (only pooled results are available), and therefore we were unable to calculate I^2 and tau, we did not assume statistical and clinical heterogeneity, due to the homogeneity in the design of the Harvard cohort studies.

c. Downgraded by one level for imprecision, since 95% CI crosses threshold of a minimal important difference (absolute risk: 5 fewer and/or 5 more events per 1.000).

d. Downgraded by one level for indirectness, since the food category was "nuts and legumes", as the health association is difficult to assign to one these two foods.

Supplementary table 8: GRADE assessment for the substitution analyses regarding fatal and non-fatal coronary artery disease

N of studies	Study design	Risk of bias	Inconsistency	Indirectness	Imprecision	Other considerations	Number of cases/participants	Relative effect (95% CI)	Absolute effect (95% CI)	Certainty
**Whole fat dairy substituted with red meat**										
3	observational studies	serious[a]	not serious	not serious	serious[b]	none	8765/139474 (6.3%)	**RR 1.12** (1.07 to 1.17)	**8 more per 1.000** (from 4 more to 11 more)	⊕⊕◯◯ Low
**Low fat dairy substituted with red meat**										
3	observational studies	serious[a]	not serious	not serious	not serious	none	8765/139474 (6.3%)	**RR 1.13** (1.08 to 1.18)	**8 more per 1.000** (from 5 more to 11 more)	⊕⊕⊕◯ Moderate
**Whole fat dairy substituted with poultry**										
2	observational studies	serious[a]	not serious	not serious	serious[b]	none	4309/96202 (4.5%)	**RR 0.90** (0.79 to 1.02)	**4 fewer per 1.000** (from 9 fewer to 1 more)	⊕⊕◯◯ Low

N of studies	Study design	Risk of bias	Inconsistency	Indirectness	Imprecision	Other considerations	Number of cases/participants	Relative effect (95% CI)	Absolute effect (95% CI)	Certainty
**Low fat dairy substituted with poultry**										
2	observational studies	serious[a]	not serious	not serious	serious[b]	none	4309/96202 (4.5%)	**RR 0.94** (0.82 to 1.07)	**3 fewer per 1.000** (from 8 fewer to 3 more)	⊕⊕◯◯ Low
**Whole fat dairy substituted with fish/seafood**										
2	observational studies	serious[a]	not serious	not serious	serious[b]	none	4309/96202 (4.5%)	**RR 0.93** (0.73 to 1.18)	**3 fewer per 1.000** (from 12 fewer to 8 more)	⊕⊕◯◯ Low
**Low fat dairy substituted with fish/seafood**										
2	observational studies	serious[a]	not serious	not serious	serious[b]	none	4309/96202 (4.5%)	**RR 0.89** (0.76 to 1.05)	**5 fewer per 1.000** (from 11 fewer to 2 more)	⊕⊕◯◯ Low
**Butter substituted with olive oil**										

N of studies	Study design	Risk of bias	Inconsistency	Indirectness	Imprecision	Other considerations	Number of cases/participants	Relative effect (95% CI)	Absolute effect (95% CI)	Certainty
2	observational studies	serious[a]	not serious	not serious	serious[b]	none	6034/92978 (6.5%)	**RR 0.94** (0.89 to 1.01)	**4 fewer per 1.000** (from 7 fewer to 1 more)	⊕⊕◯◯ Low
**Dairy substituted with avocado**										
2	observational studies	serious[a]	not serious	not serious	serious[b]	none	9185/110487 (8.3%)	**RR 0.42** (0.17 to 1.05)	**48 fewer per 1.000** (from 69 fewer to 4 more)	⊕⊕◯◯ Low
**Yogurt substituted with avocado**										
2	observational studies	serious[a]	not serious	not serious	very serious[d]	none	9185/110487 (8.3%)	**RR 0.31** (0.16 to 0.59)	**57 fewer per 1.000** (from 70 fewer to 34 fewer)	⊕◯◯◯ Very low
**Cheese substituted with avocado**										

N of studies	Study design	Risk of bias	Inconsistency	Indirectness	Imprecision	Other considerations	Number of cases/participants	Relative effect (95% CI)	Absolute effect (95% CI)	Certainty
2	observational studies	serious[a]	not serious	not serious	not serious	none	9185/110487 (8.3%)	**RR 0.81** (0.72 to 0.90)	**16 fewer per 1.000** (from 23 fewer to 8 fewer)	⊕⊕⊕◯ Moderate
**Butter substituted with avocado**										
2	observational studies	serious[a]	not serious	not serious	serious[b]	none	9185/110487 (8.3%)	**RR 0.95** (0.92 to 0.99)	**4 fewer per 1.000** (from 7 fewer to 1 fewer)	⊕⊕◯◯ Low
**Milk substituted with sugar sweetened beverages**										
6	observational studies	serious[a]	serious[c]	not serious	not serious	none	5878/284345 (2.1%)	**RR 1.01** (0.98 to 1.05)	**0 fewer per 1.000** (from 0 fewer to 1 more)	⊕⊕◯◯ Low
**Whole fat milk substituted with sugar sweetened beverages**										

N of studies	Study design	Risk of bias	Inconsistency	Indirectness	Imprecision	Other considerations	Number of cases/participants	Relative effect (95% CI)	Absolute effect (95% CI)	Certainty
6	observational studies	serious[a]	not serious	not serious	not serious	none	5878/284345 (2.1%)	**RR 1.01** (0.96 to 1.06)	**0 fewer per 1.000** (from 1 fewer to 1 more)	⊕⊕⊕◯ Moderate
**Low fat milk substituted with sugar sweetened beverages**										
6	observational studies	serious[a]	not serious	not serious	not serious	none	5878/284345 (2.1%)	**RR 1.02** (0.98 to 1.05)	**0 fewer per 1.000** (from 0 fewer to 1 more)	⊕⊕⊕◯ Moderate

CI: confidence interval; **RR:** risk ratio

Explanations

a. Downgraded by one level for risk of bias, since all studies were rated with a moderate risk of bias.

b. Downgraded by one level for imprecision, since 95% CI crosses threshold of a minimal important difference (absolute risk: 5 fewer and/or 5 more events per 1.000).

c. Downgraded by one level for inconsistency, since I^2 was substantial.

d. Downgraded by two levels for imprecision, since the 95% CI did not cross the threshold of a minimal important difference (absolute risk: 5 fewer and/or 5 more events per 1.000) since the relative effect was large (RR<0.7), and the ratio of the upper to the lower boundary of the 95% CI was more than three.

Supplementary table 9: GRADE assessment for the substitution analyses regarding fatal and non-fatal stroke

N of studies	Study design	Risk of bias	Inconsistency	Indirectness	Imprecision	Other considerations	Number of cases/participants	Relative effect (95% CI)	Absolute effect (95% CI)	Certainty
**Whole fat milk substituted with low fat milk**										
2	observational studies	serious[a]	not serious	not serious	not serious	none	3156/92097 (3.4%)	**RR 0.97** (0.91 to 1.04)	**1 fewer per 1.000** (from 3 fewer to 1 more)	⊕⊕⊕◯ Moderate
**Whole fat milk substituted with cheese**										
2	observational studies	serious[a]	not serious	not serious	not serious	none	3156/92097 (3.4%)	**RR 0.94** (0.88 to 1.00)	**2 fewer per 1.000** (from 4 fewer to 0 fewer)	⊕⊕⊕◯ Moderate
**Buttermilk substituted with low fat milk**										
2	observational studies	serious[a]	not serious	not serious	not serious	none	3156/92097 (3.4%)	**RR 0.99** (0.92 to 1.06)	**0 fewer per 1.000** (from 3 fewer to 2 more)	⊕⊕⊕◯ Moderate

N of studies	Study design	Risk of bias	Inconsistency	Indirectness	Imprecision	Other considerations	Number of cases/participants	Relative effect (95% CI)	Absolute effect (95% CI)	Certainty
**Buttermilk substituted with whole fat milk**										
2	observational studies	serious[a]	not serious	not serious	not serious	none	3156/92097 (3.4%)	**RR 1.01** (0.92 to 1.10)	**0 fewer per 1.000** (from 3 fewer to 3 more)	⊕⊕⊕◯ Moderate
**Buttermilk substituted with cheese**										
2	observational studies	serious[a]	not serious	not serious	not serious	none	3156/92097 (3.4%)	**RR 0.96** (0.89 to 1.04)	**1 fewer per 1.000** (from 4 fewer to 1 more)	⊕⊕⊕◯ Moderate
**Butter substituted with cheese**										
2	observational studies	serious[a]	not serious	not serious	not serious	none	3156/92097 (3.4%)	**RR 0.98** (0.95 to 1.01)	**1 fewer per 1.000** (from 2 fewer to 0 fewer)	⊕⊕⊕◯ Moderate
**Whole fat dairy substituted with red meat**										

N of studies	Study design	Risk of bias	Inconsistency	Indirectness	Imprecision	Other considerations	Number of cases/participants	Relative effect (95% CI)	Absolute effect (95% CI)	Certainty
2	observational studies	serious[a]	not serious[b]	not serious	not serious	none	4030/127160 (3.2%)	**RR 1.10** (1.04 to 1.17)	**3 more per 1.000** (from 1 more to 5 more)	⊕⊕⊕◯ Moderate
**Low fat dairy substituted with red meat**										
2	observational studies	serious[a]	not serious[b]	not serious	not serious	none	4030/127160 (3.2%)	**RR 1.11** (1.04 to 1.17)	**3 more per 1.000** (from 1 more to 5 more)	⊕⊕⊕◯ Moderate
**Butter substituted with olive oil**										
2	observational studies	serious[a]	not serious	not serious	not serious	none	3802/92978 (4.1%)	**RR 0.96** (0.89 to 1.04)	**2 fewer per 1.000** (from 4 fewer to 2 more)	⊕⊕⊕◯ Moderate
**Dairy substituted with avocado**										

N of studies	Study design	Risk of bias	Inconsistency	Indirectness	Imprecision	Other considerations	Number of cases/participants	Relative effect (95% CI)	Absolute effect (95% CI)	Certainty
2	observational studies	serious[a]	not serious	not serious	serious[c]	none	5290/110487 (4.8%)	**RR 0.90** (0.31 to 2.82)	**5 fewer per 1.000** (from 33 fewer to 87 more)	⊕⊕◯◯ Low
**Yogurt substituted with avocado**										
2	observational studies	serious[a]	not serious	not serious	serious[c]	none	5290/110487 (4.8%)	**RR 1.28** (0.62 to 2.70)	**13 more per 1.000** (from 18 fewer to 81 more)	⊕⊕◯◯ Low
**Cheese substituted with avocado**										
2	observational studies	serious[a]	not serious	not serious	serious[c]	none	5290/110487 (4.8%)	**RR 1.05** (0.92 to 1.20)	**2 more per 1.000** (from 4 fewer to 10 more)	⊕⊕◯◯ Low
**Butter substituted with avocado**										

N of studies	Study design	Risk of bias	Inconsistency	Indirectness	Imprecision	Other considerations	Number of cases/participants	Relative effect (95% CI)	Absolute effect (95% CI)	Certainty
2	observational studies	serious[a]	not serious	not serious	not serious	none	5290/110487 (4.8%)	**RR 0.99** (0.94 to 1.03)	**0 fewer per 1.000** (from 3 fewer to 1 more)	⊕⊕⊕◯ Moderate

CI: confidence interval; **RR:** risk ratio

Explanations

a. Downgraded by one level for risk of bias, since all studies were rated with a moderate risk of bias.

b. Although RR and 95% CI of the individual cohort studies are not available (only pooled results are available), and therefore we were unable to calculate I^2 and tau, we did not assume statistical and clinical heterogeneity, due to the homogeneity in the design of the Harvard cohort studies.

c. Downgraded by one level for imprecision, since 95% CI crosses threshold of a minimal important difference (absolute risk: 5 fewer and/or 5 more events per 1.000).

Supplementary table 10: GRADE assessment for the substitution analyses regarding type 2 diabetes

N of studies	Study design	Risk of bias	Inconsistency	Indirectness	Imprecision	Other considerations	Number of cases/participants	Relative effect (95% CI)	Absolute effect (95% CI)	Certainty
**Whole fat milk substituted with low fat milk**										
2	observational studies	serious[a]	not serious	not serious	not serious	none	8604/90259 (9.5%)	**RR 0.99** (0.96 to 1.03)	**1 fewer per 1.000** (from 4 fewer to 3 more)	⊕⊕⊕◯ Moderate
**Buttermilk substituted with low fat milk**										
2	observational studies	serious[a]	not serious	not serious	serious[b]	none	8604/90259 (9.5%)	**RR 0.99** (0.94 to 1.04)	**1 fewer per 1.000** (from 6 fewer to 4 more)	⊕⊕◯◯ Low
**Buttermilk substituted with whole fat milk**										
2	observational studies	serious[a]	not serious	not serious	serious[b]	none	8604/90259 (9.5%)	**RR 1.00** (0.94 to 1.06)	**0 fewer per 1.000** (from 6 fewer to 6 more)	⊕⊕◯◯ Low
**Whole fat milk substituted with whole fat yogurt**										

N of studies	Study design	Risk of bias	Inconsistency	Indirectness	Imprecision	Other considerations	Number of cases/participants	Relative effect (95% CI)	Absolute effect (95% CI)	Certainty
2	observational studies	serious[a]	not serious	not serious	serious[b]	none	8604/90259 (9.5%)	**RR 0.93** (0.77 to 1.12)	**7 fewer per 1.000** (from 22 fewer to 11 more)	⊕⊕◯◯ Low
**Buttermilk substituted with whole fat yogurt**										
2	observational studies	serious[a]	not serious	not serious	serious[b]	none	8604/90259 (9.5%)	**RR 0.92** (0.78 to 1.08)	**8 fewer per 1.000** (from 21 fewer to 8 more)	⊕⊕◯◯ Low
**Low fat milk substituted with whole fat yogurt**										
2	observational studies	serious[a]	not serious	not serious	serious[b]	none	8604/90259 (9.5%)	**RR 0.89** (0.83 to 0.96)	**10 fewer per 1.000** (from 16 fewer to 4 fewer)	⊕⊕◯◯ Low
**Milk substituted with sugar sweetened beverages**										

N of studies	Study design	Risk of bias	Inconsistency	Indirectness	Imprecision	Other considerations	Number of cases/participants	Relative effect (95% CI)	Absolute effect (95% CI)	Certainty
2	observational studies	very serious[c]	not serious	not serious	not serious	none	15051/110564 (13.6%)	**RR 1.11** (1.07 to 1.16)	**15 more per 1.000** (from 10 more to 22 more)	⊕⊕◯◯ Low
**Milk substituted with fruit juice**										
2	observational studies	very serious[c]	serious[d]	not serious	serious[b]	none	15051/110564 (13.6%)	**RR 1.07** (0.91 to 1.25)	**10 more per 1.000** (from 12 fewer to 34 more)	⊕◯◯◯ Very low
**Low fat dairy substituted with red meat**										
3	observational studies	serious[a]	not serious[e]	not serious	not serious	none	13759/204157 (6.7%)	**RR 1.20** (1.16 to 1.25)	**13 more per 1.000** (from 11 more to 17 more)	⊕⊕⊕◯ Moderate
**Low fat dairy substituted with processed red meat**										

N of studies	Study design	Risk of bias	Inconsistency	Indirectness	Imprecision	Other considerations	Number of cases/participants	Relative effect (95% CI)	Absolute effect (95% CI)	Certainty
3	observational studies	serious[a]	not serious[e]	not serious	not serious	none	13759/204157 (6.7%)	**RR 1.41** (1.33 to 1.49)	**28 more per 1.000** (from 22 more to 33 more)	⊕⊕⊕◯ Moderate
**Low fat dairy substituted with unprocessed red meat**										
3	observational studies	serious[a]	not serious[e]	not serious	not serious	none	13759/204157 (6.7%)	**RR 1.19** (1.15 to 1.23)	**13 more per 1.000** (from 10 more to 16 more)	⊕⊕⊕◯ Moderate
**Dairy substituted with whole grains**										
3	observational studies	serious[a]	not serious[e]	not serious	not serious	none	15580/205802 (7.6%)	**RR 0.97** (0.95 to 1.00)	**2 fewer per 1.000** (from 4 fewer to 0 fewer)	⊕⊕⊕◯ Moderate
**Dairy substituted with nuts/peanuts**										

N of studies	Study design	Risk of bias	Inconsistency	Indirectness	Imprecision	Other considerations	Number of cases/participants	Relative effect (95% CI)	Absolute effect (95% CI)	Certainty
3	observational studies	serious[a]	not serious[e]	not serious	not serious	none	15580/205802 (7.6%)	**RR 0.98** (0.93 to 1.03)	**2 fewer per 1.000** (from 5 fewer to 2 more)	⊕⊕⊕◯ Moderate
**Milk substituted with red meat**										
3	observational studies	serious[a]	not serious	not serious	serious[b]	none	28246/169757 (16.6%)	**RR 1.03** (1.00 to 1.07)	**5 more per 1.000** (from 0 fewer to 12 more)	⊕⊕◯◯ Low
**Milk substituted with processed meat**										
3	observational studies	serious[a]	not serious	not serious	not serious	none	28246/169757 (16.6%)	**RR 1.10** (1.07 to 1.14)	**17 more per 1.000** (from 12 more to 23 more)	⊕⊕⊕◯ Moderate
**Milk substituted with eggs**										

N of studies	Study design	Risk of bias	Inconsistency	Indirectness	Imprecision	Other considerations	Number of cases/participants	Relative effect (95% CI)	Absolute effect (95% CI)	Certainty
2	observational studies	serious[a]	serious[d]	not serious	serious[b]	none	16505/143297 (11.5%)	**RR 1.01** (0.78 to 1.31)	**1 more per 1.000** (from 25 fewer to 36 more)	⊕◯◯◯ Very low
**Milk substituted with poultry**										
2	observational studies	serious[a]	not serious	not serious	serious[b]	none	16505/143297 (11.5%)	**RR 0.98** (0.85 to 1.14)	**2 fewer per 1.000** (from 17 fewer to 16 more)	⊕⊕◯◯ Low
**Yogurt substituted with red meat**										
3	observational studies	serious[a]	not serious	not serious	not serious	none	28246/169757 (16.6%)	**RR 1.21** (1.05 to 1.41)	**35 more per 1.000** (from 8 more to 68 more)	⊕⊕⊕◯ Moderate
**Yogurt substituted with processed meat**										

N of studies	Study design	Risk of bias	Inconsistency	Indirectness	Imprecision	Other considerations	Number of cases/participants	Relative effect (95% CI)	Absolute effect (95% CI)	Certainty
3	observational studies	serious[a]	not serious	not serious	not serious	none	28246/169757 (16.6%)	**RR 1.43** (1.27 to 1.62)	**72 more per 1.000** (from 45 more to 103 more)	⊕⊕⊕◯ Moderate
**Yogurt substituted with eggs**										
2	observational studies	serious[a]	not serious	not serious	not serious	none	16505/143297 (11.5%)	**RR 1.44** (1.28 to 1.62)	**51 more per 1.000** (from 32 more to 71 more)	⊕⊕⊕◯ Moderate
**Yogurt substituted with poultry**										
2	observational studies	serious[a]	not serious	not serious	serious[b]	none	16505/143297 (11.5%)	**RR 1.11** (0.99 to 1.23)	**13 more per 1.000** (from 1 fewer to 26 more)	⊕⊕◯◯ Low
**Cheese substituted with red meat**										

N of studies	Study design	Risk of bias	Inconsistency	Indirectness	Imprecision	Other considerations	Number of cases/participants	Relative effect (95% CI)	Absolute effect (95% CI)	Certainty
3	observational studies	serious[a]	not serious	not serious	serious[b]	none	28246/169757 (16.6%)	**RR 1.08** (1.01 to 1.15)	**13 more per 1.000** (from 2 more to 25 more)	⊕⊕◯◯ Low

Cheese substituted with processed meat

3	observational studies	serious[a]	not serious	not serious	not serious	none	28246/169757 (16.6%)	**RR 1.14** (1.08 to 1.20)	**23 more per 1.000** (from 13 more to 33 more)	⊕⊕⊕◯ Moderate

Cheese substituted with eggs

2	observational studies	serious[a]	not serious	not serious	not serious	none	16505/143297 (11.5%)	**RR 1.14** (1.10 to 1.19)	**16 more per 1.000** (from 12 more to 22 more)	⊕⊕⊕◯ Moderate

Cheese substituted with poultry

N of studies	Study design	Risk of bias	Inconsistency	Indirectness	Imprecision	Other considerations	Number of cases/participants	Relative effect (95% CI)	Absolute effect (95% CI)	Certainty
2	observational studies	serious[a]	not serious	not serious	serious[b]	none	16505/143297 (11.5%)	**RR 1.08** (0.96 to 1.22)	**9 more per 1.000** (from 5 fewer to 25 more)	⊕⊕◯◯ Low
**Butter substituted with olive oil**										
2	observational studies	very serious[f]	not serious[e]	not serious	not serious	none	9652/145087 (6.7%)	**RR 0.95** (0.92 to 0.98)	**3 fewer per 1.000** (from 5 fewer to 1 fewer)	⊕⊕◯◯ Low

CI: confidence interval; **RR:** risk ratio

Explanations

a. Downgraded by one level for risk of bias, since all studies were rated with a moderate risk of bias.

b. Downgraded by one level for imprecision, since 95% CI crosses threshold of a minimal important difference (absolute risk: 5 fewer and/or 5 more events per 1.000).

c. Downgraded by two levels for risk of bias, since one study was rated with a serious risk of bias, due to confounding (effect estimates not adjusted for energy intake).

d. Downgraded by one level for inconsistency since point estimates and 95% CI did not fully overlap between studies and I^2 was substantial.

e. Although RR and 95% CI of the individual cohort studies are not available (only pooled results are available), and therefore we were unable to calculate I^2 and tau, we did not assume statistical and clinical heterogeneity, due to the homogeneity in the design of the Harvard cohort studies.

f. Downgraded by two levels for risk of bias, since all studies were rated with a serious risk of bias, due to confounding (effect estimates not adjusted for Body Mass Index).

Replacement	Replacement unit	Study	Cohort	No of participants	No of outcome events	Summary risk ratio (95% CI)	Certainty of Evidence
**All-cause mortality**							
Dairy (200 g/d) replaced with							
Red and processed meat	130 g/d	Ding 2019	NHS, NHS II, HPFS	217'755	51'438	1.05 (1.01, 1.09)	Low
Poultry	125 g/d	Ding 2019	NHS, NHS II, HPFS	217'755	51'438	0.93 (0.85, 1.03)	Low
Nuts and legumes	28 g/d	Ding 2019	NHS, NHS II, HPFS	217'755	51'438	0.86 (0.78, 0.95)	Low
Whole grains	30 g/d	Ding 2019	NHS, NHS II, HPFS	217'755	51'438	0.89 (0.84, 0.93)	Moderate
Low fat dairy (200 g/d) replaced with							
Red meat	142 g/d	Pan 2012	NHS, HPFS	121'342	23'926	1.11 (1.06, 1.16)	Moderate
Processed red meat	85 g/d	Pan 2012	NHS, HPFS	121'342	23'926	1.19 (1.11, 1.28)	Moderate
Unprocessed red meat	85 g/d	Pan 2012	NHS, HPFS	121'342	23'926	1.10 (1.02, 1.19)	Low

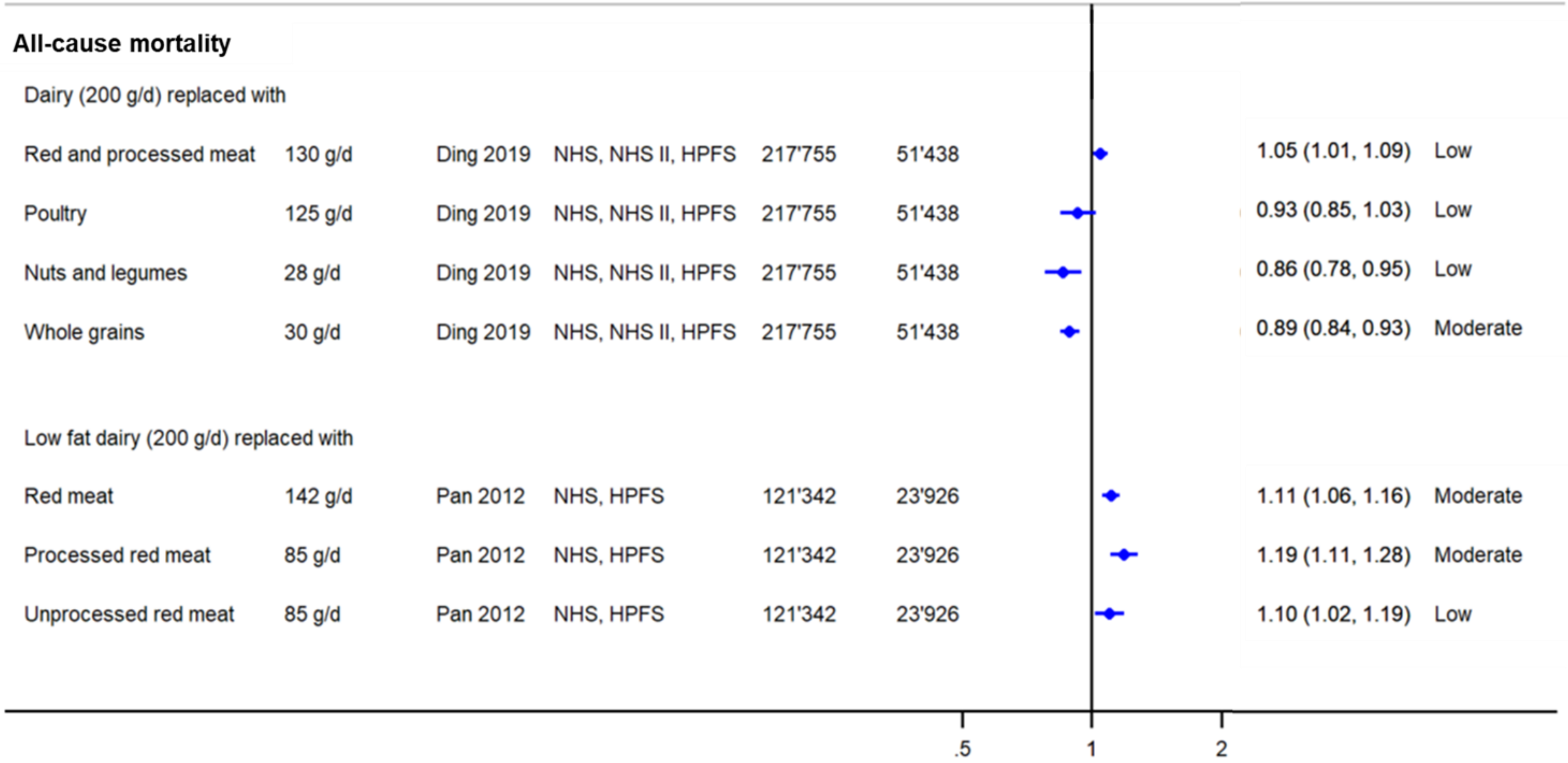

Supplementary figure 2: Extracted pooled results for all-cause mortality (replacement and substitution are used synonymously)

Author	Year	Cohort	Food	Food unit	Replacement	Replacement unit	SRR (95% CI)	% Weight
Zhang	2021	NIH-AARP Diet and Health Study	Butter	5 g/d	Olive oil	5 g/d	0.96 (0.95, 0.98)	48.31
Guasch-Ferre	2022	NHS, HPFS	Butter	5 g/d	Olive oil	5 g/d	0.93 (0.92, 0.94)	51.69
Overall (I-squared = 90.6%, p = 0.001)							0.94 (0.92, 0.97)	100.00
Inestimable predictive distribution with <3 studies							. (- , -)	

NOTE: Weights are from random effects analysis

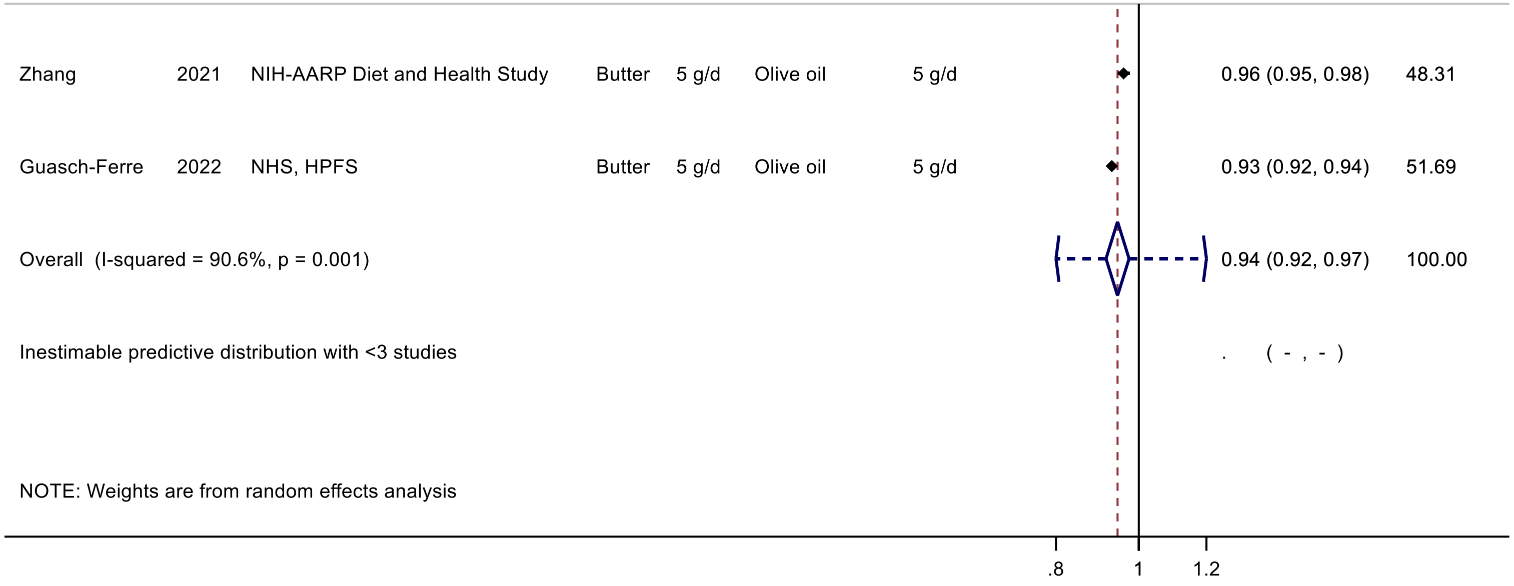

Supplementary figure 3: Meta-analysis comparing the substituting effect of substituting butter with an equal amount of olive oil on all-cause mortality (replacement and substitution are used synonymously)

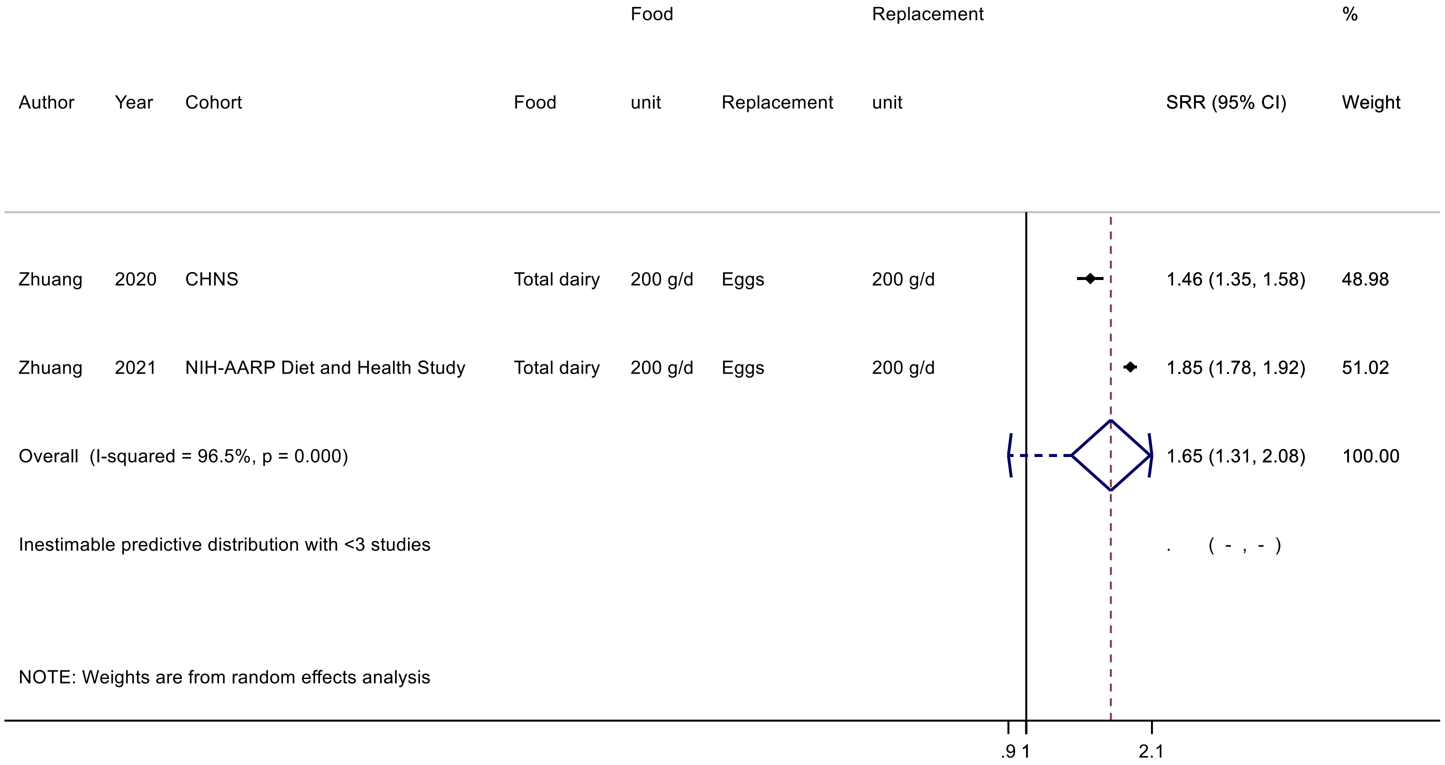

Supplementary figure 4: Meta-analysis comparing the substituting effect of substituting dairy with an equal amount of eggs on all-cause mortality (replacement and substitution are used synonymously)

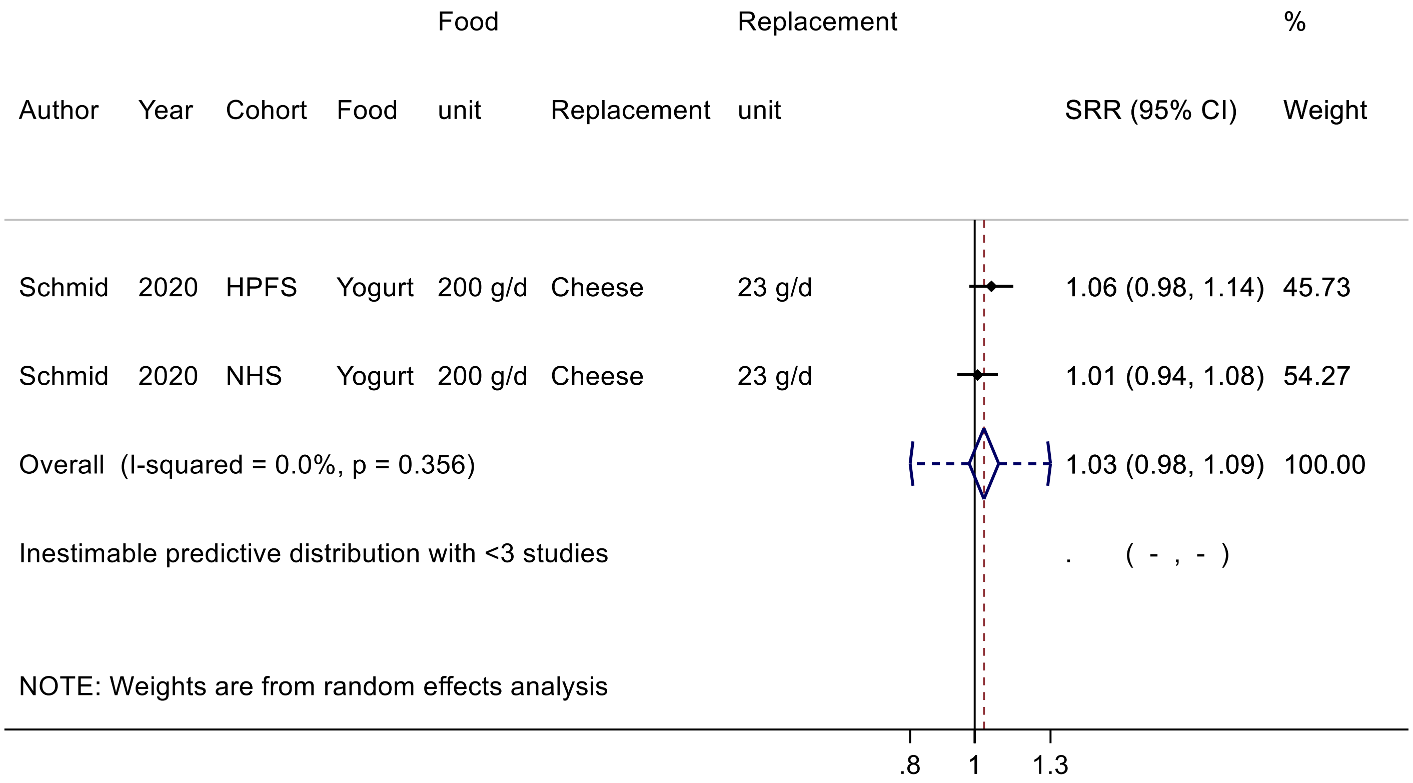

Supplementary figure 5: Meta-analysis comparing the substituting effect of substituting yogurt with cheese on all-cause mortality (replacement and substitution are used synonymously)

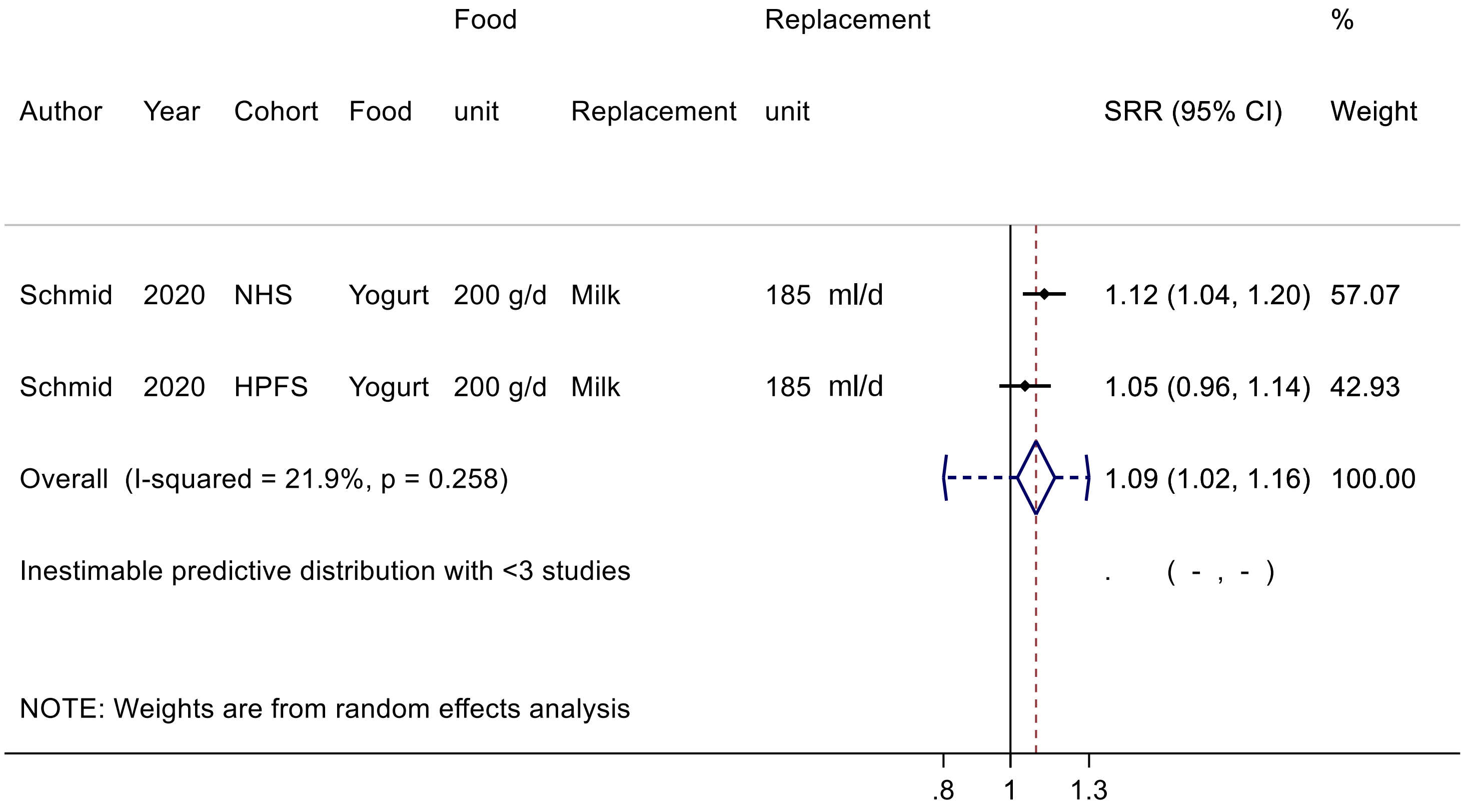

Supplementary figure 6: Meta-analysis comparing the substituting effect of substituting yogurt with milk on all-cause mortality (replacement and substitution are used synonymously)

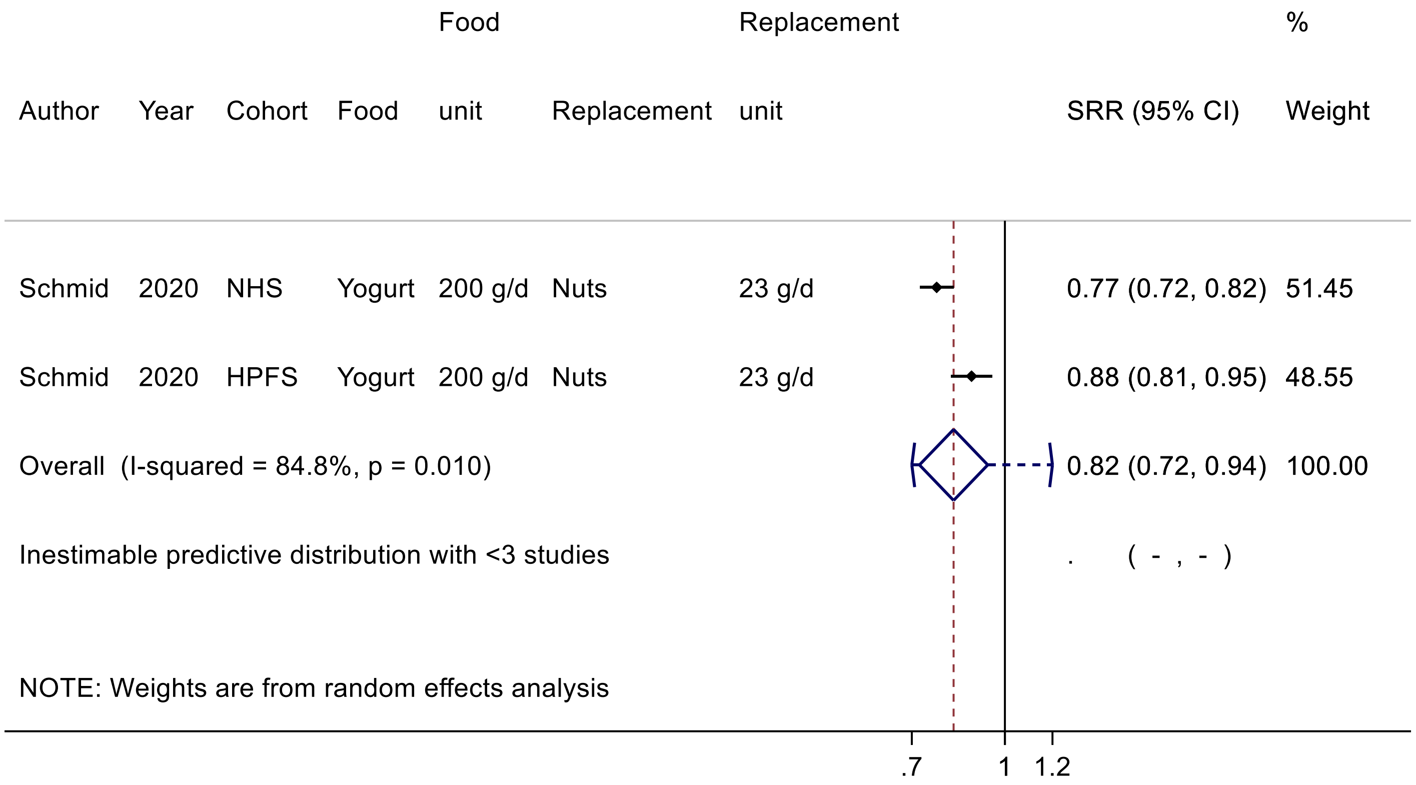

Supplementary figure 7: Meta-analysis comparing the substituting effect of substituting yogurt with nuts on all-cause mortality
(replacement and substitution are used synonymously)

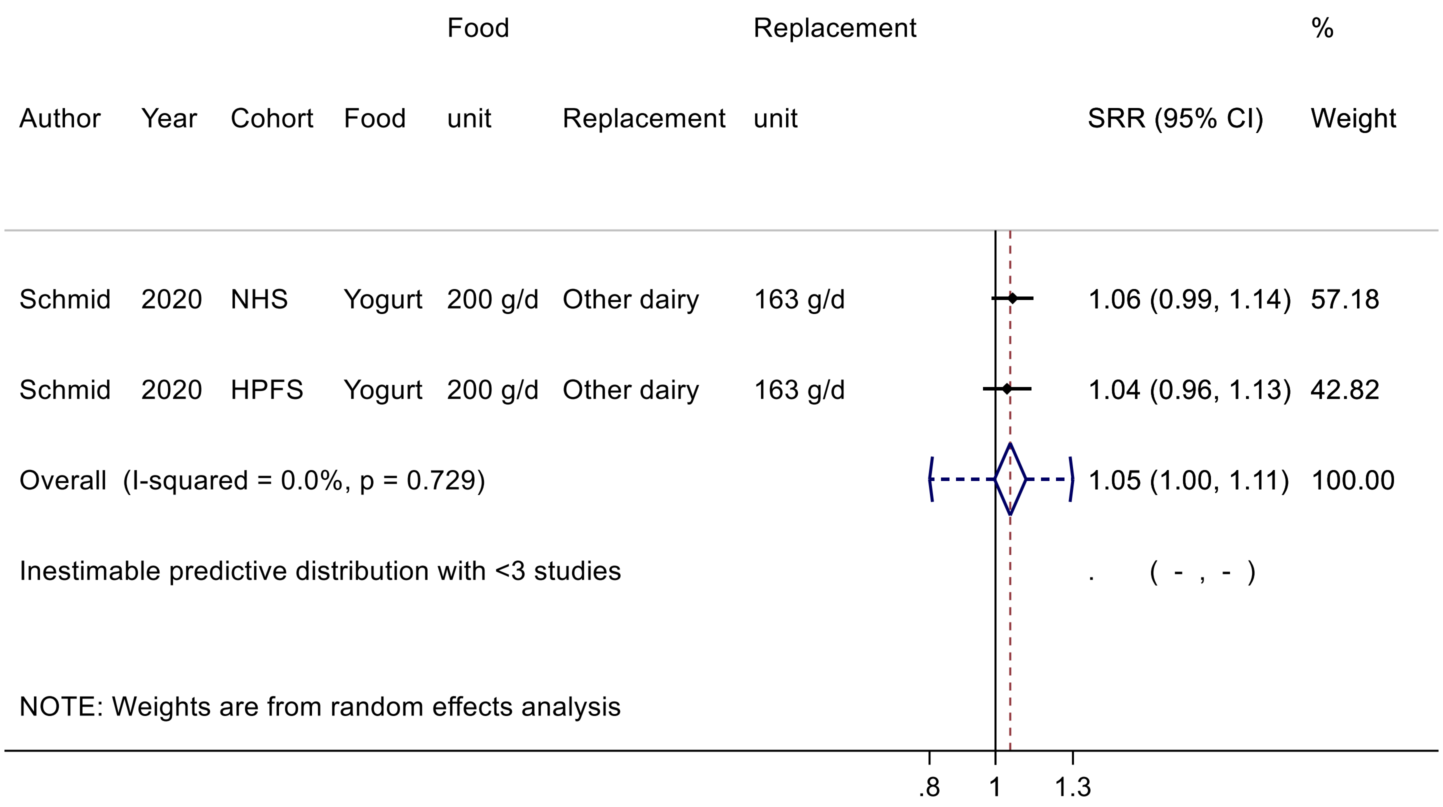

Supplementary figure 8: Meta-analysis comparing the substituting effect of substituting yogurt of other dairy on all-cause mortality (replacement and substitution are used synonymously)

Author	Year	Cohort	Food	Food unit	Replacement	Replacement unit	SRR (95% CI)	% Weight
Schmid	2020	HPFS	Yogurt	200 g/d	Processed meat	30 g/d	1.13 (1.04, 1.23)	47.49
Schmid	2020	NHS	Yogurt	200 g/d	Processed meat	30 g/d	1.25 (1.16, 1.34)	52.51
Overall (I-squared = 68.4%, p = 0.075)							1.19 (1.08, 1.32)	100.00
Inestimable predictive distribution with <3 studies							. (- , -)	

NOTE: Weights are from random effects analysis

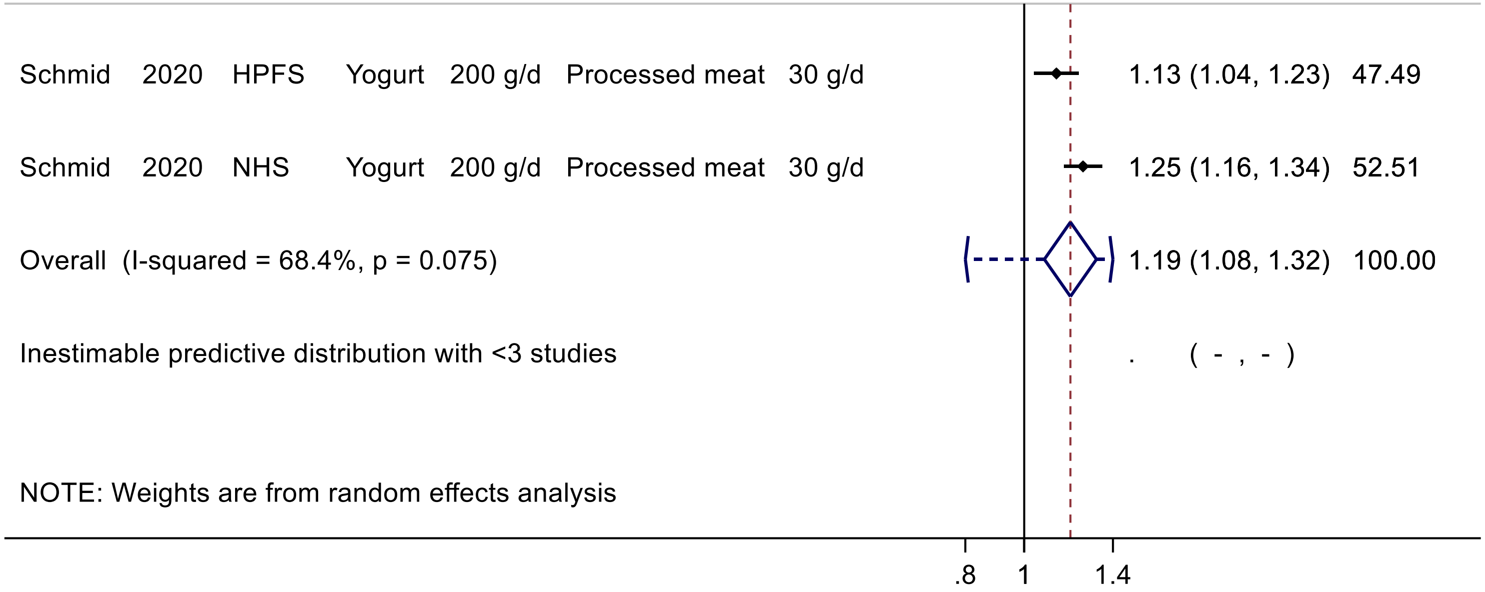

Supplementary figure 9: Meta-analysis comparing the substituting effect of substituting yogurt with processed meat on all-cause mortality (replacement and substitution are used synonymously)

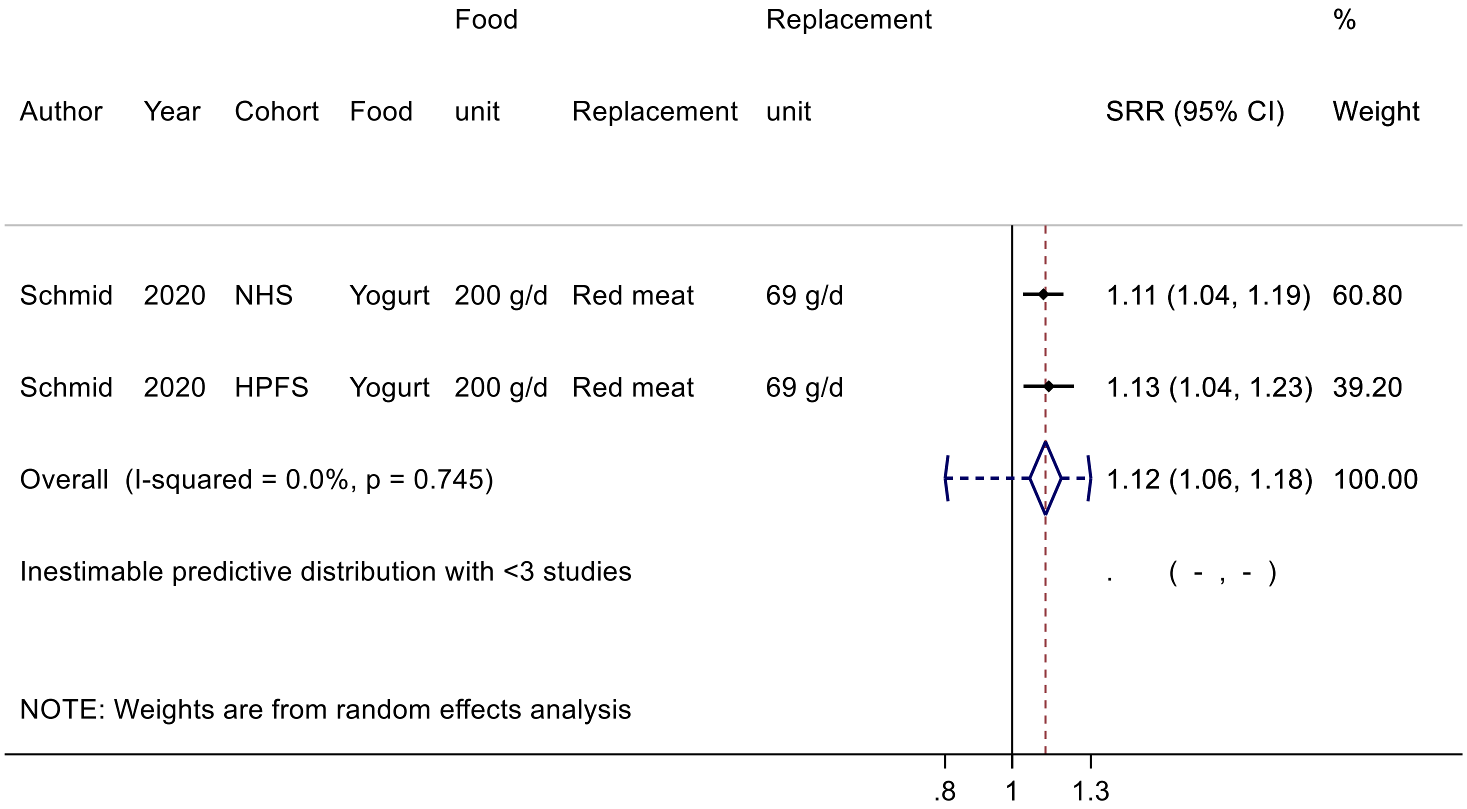

Supplementary figure 10: Meta-analysis comparing the substituting effect of substituting yogurt with red meat on all-cause mortality
(replacement and substitution are used synonymously)

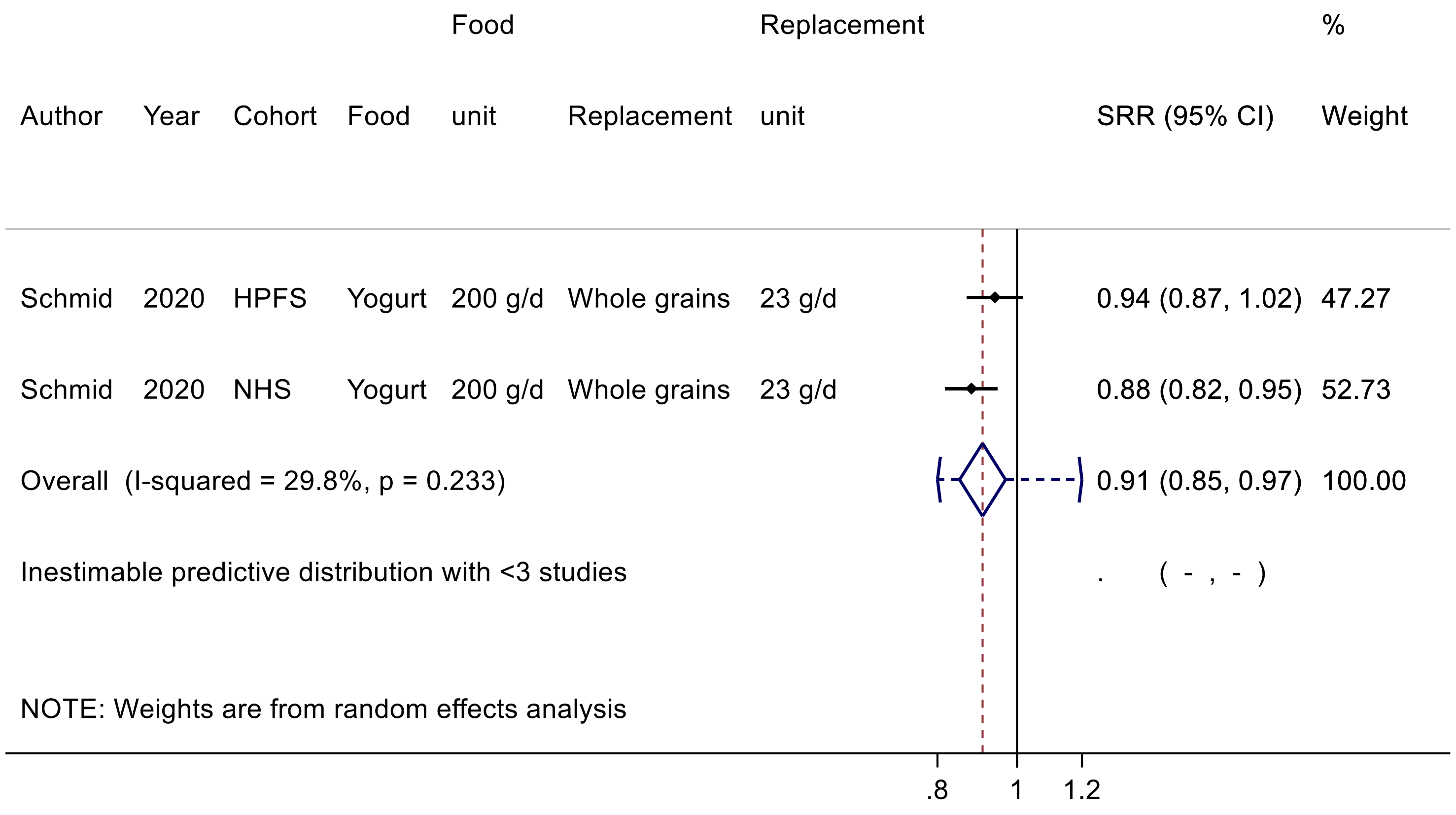

Supplementary figure 11: Meta-analysis comparing the substituting effect of substituting yogurt with whole grains on all-cause mortality (replacement and substitution are used synonymously)

Replacement	Replacement unit	Study	Cohort	No of participants	No of outcome events	Summary risk ratio (95% CI)	Certainty of Evidence
**CVD mortality**							
Dairy (200 g/d) replaced with							
Red and processed meat	130 g/d	Ding 2019	NHS, NHS II, HPFS	217'755	12'143	1.08 (1.02, 1.15)	Low
Poultry	125 g/d	Ding 2019	NHS, NHS II, HPFS	217'755	12'143	1.01 (0.84, 1.20)	Low
Nuts and legumes	28-50 g/d	Ding 2019	NHS, NHS II, HPFS	217'755	12'143	0.89 (0.84, 0.93)	Very low
Whole grains	30 g/d	Ding 2019	NHS, NHS II, HPFS	217'755	12'143	0.93 (0.85, 1.01)	Low
**CVD**							
Dairy (200 g/d) replaced with							
Avocado	200 g/d	Pacheco 2022*	NHS, HPFS	110'487	14'274	0.62 (0.31, 1.28)	Low
Yogurt(200 g/d) replaced with							
Avocado	200 g/d	Pacheco 2022*	NHS, HPFS	110'487	14'274	0.56 (0.33, 0.95)	Low
Cheese(30 g/d) replaced with							
Avocado	30 g/d	Pacheco 2022*	NHS, HPFS	110'487	14'274	0.90 (0.82, 0.98)	Low
Butter(5 g/d) replaced with							
Olive oil	5 g/d	Guasch-Ferre 2020	NHS, HPFS	92'978	9'797	0.95 (0.91, 1.00)	Low
Avocado	5 g/d	Pacheco 2022*	NHS, HPFS	110'487	14'274	0.97 (0.94, 0.99)	Low

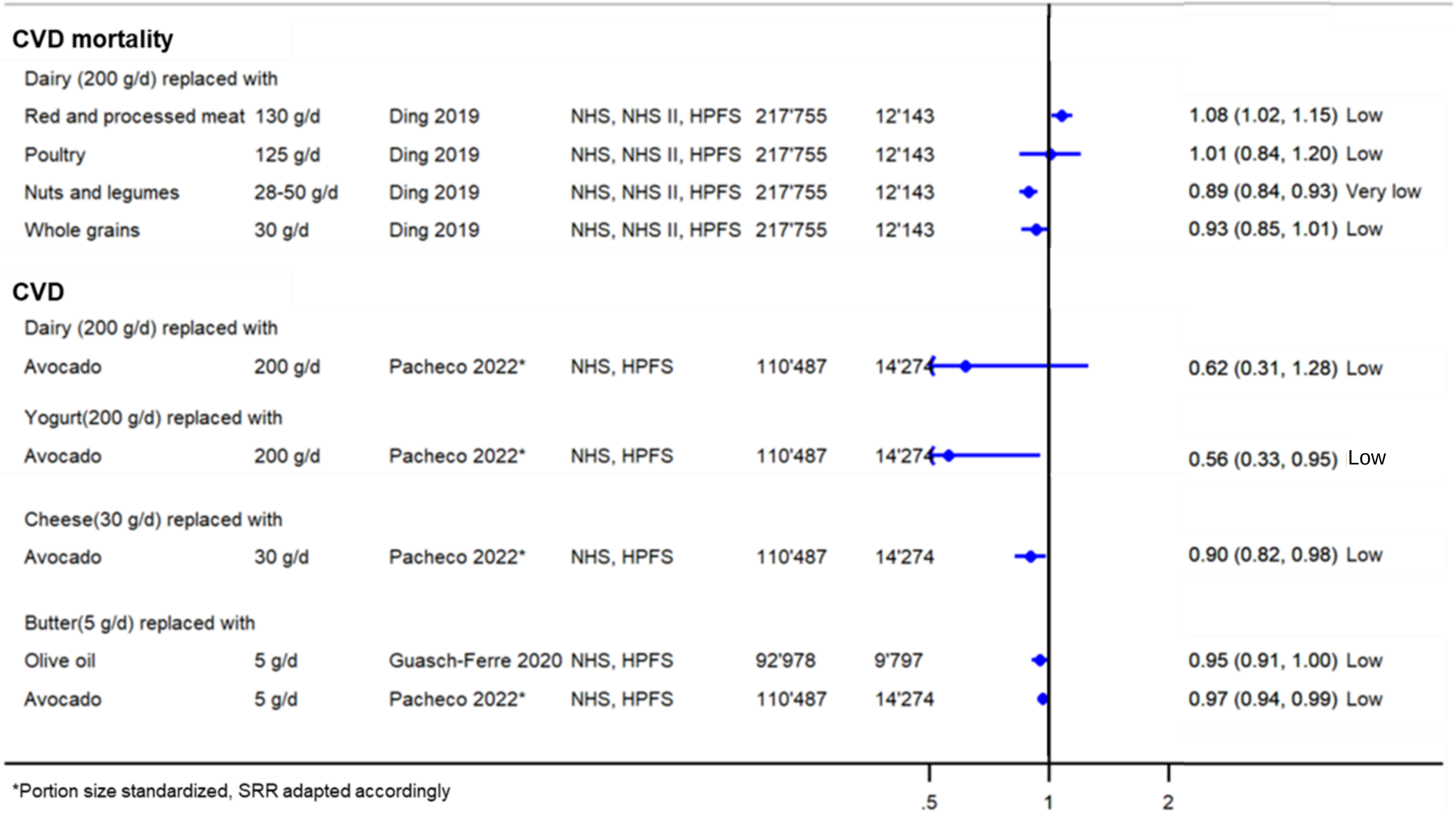

*Portion size standardized, SRR adapted accordingly

Supplementary figure 12: Extracted pooled results for cardiovascular disease (fatal and non-fatal)
(replacement and substitution are used synonymously)

Replacement	Replacement unit	Study	Cohort	No of participants	No of outcomes events	Summary risk ratio (95% CI)	Certainty of Evidence
**CHD**							
Dairy (200 g/d) replaced with Avocado	200 g/d	Pacheco 2022*	NHS, HPFS	110'487	9'185	0.42 (0.17, 1.05)	Low
Milk (200 ml/d) replaced with Sugar-sweetened beverages	200 ml/d	Keller 2020*	HPP	284'345	5'878	1.01 (0.98, 1.05)	Low
Whole fat milk (200 ml/d) replaced with Sugar-sweetened beverages	200 ml/d	Keller 2020*	HPP	284'345	5'878	1.01 (0.96, 1.06)	Moderate
Low fat milk (200 ml/d) replaced with Sugar-sweetened beverages	200 ml/d	Keller 2020*	HPP	284'345	5'878	1.02 (0.98, 1.05)	Moderate
Yogurt (200 g/d) replaced with Avocado	200 g/d	Pacheco 2022*	NHS, HPFS	110'487	9'185	0.31 (0.16, 0.59)	Very low
Cheese (30 g/d) replaced with Avocado	30 g/d	Pacheco 2022*	NHS, HPFS	110'487	9'185	0.81 (0.72, 0.90)	Moderate
Butter (5 g/d) replaced with Olive oil	5 g/d	Guasch-Ferre 2020	NHS, HPFS	92'978	6'034	0.94 (0.89, 1.01)	Low
Avocado	5 g/d	Pacheco 2022*	NHS, HPFS	110'487	9'185	0.95 (0.92, 0.99)	Low
**Stroke**							
Dairy (200 g/d) replaced with Avocado	200 g/d	Pacheco 2022*	NHS, HPFS	110'487	5'290	0.90 (0.31, 2.82)	Low
Whole fat dairy (200 g/d) replaced with Red meat	125 g/d	Bernstein 2012*	NHS, HPFS	127'160	4'030	1.10 (1.04, 1.17)	Moderate
Low fat dairy (200 g/d) replaced with Red meat	125 g/d	Bernstein 2012*	NHS, HPFS	127'160	4'030	1.11 (1.04, 1.17)	Moderate
Yogurt (200 g/d) replaced with Avocado	200 g/d	Pacheco 2022*	NHS, HPFS	110'487	5'290	1.28 (0.62, 2.70)	Low
Cheese (30 g/d) replaced with Avocado	30 g/d	Pacheco 2022*	NHS, HPFS	110'487	5'290	1.05 (0.92, 1.20)	Low
Butter (5 g/d) replaced with Olive oil	5 g/d	Guasch-Ferre 2020	NHS, HPFS	92'978	3'802	0.96 (0.89, 1.04)	Moderate
Avocado	5 g/d	Pacheco 2022*	NHS, HPFS	110'487	5'290	0.99 (0.94, 1.03)	Moderate

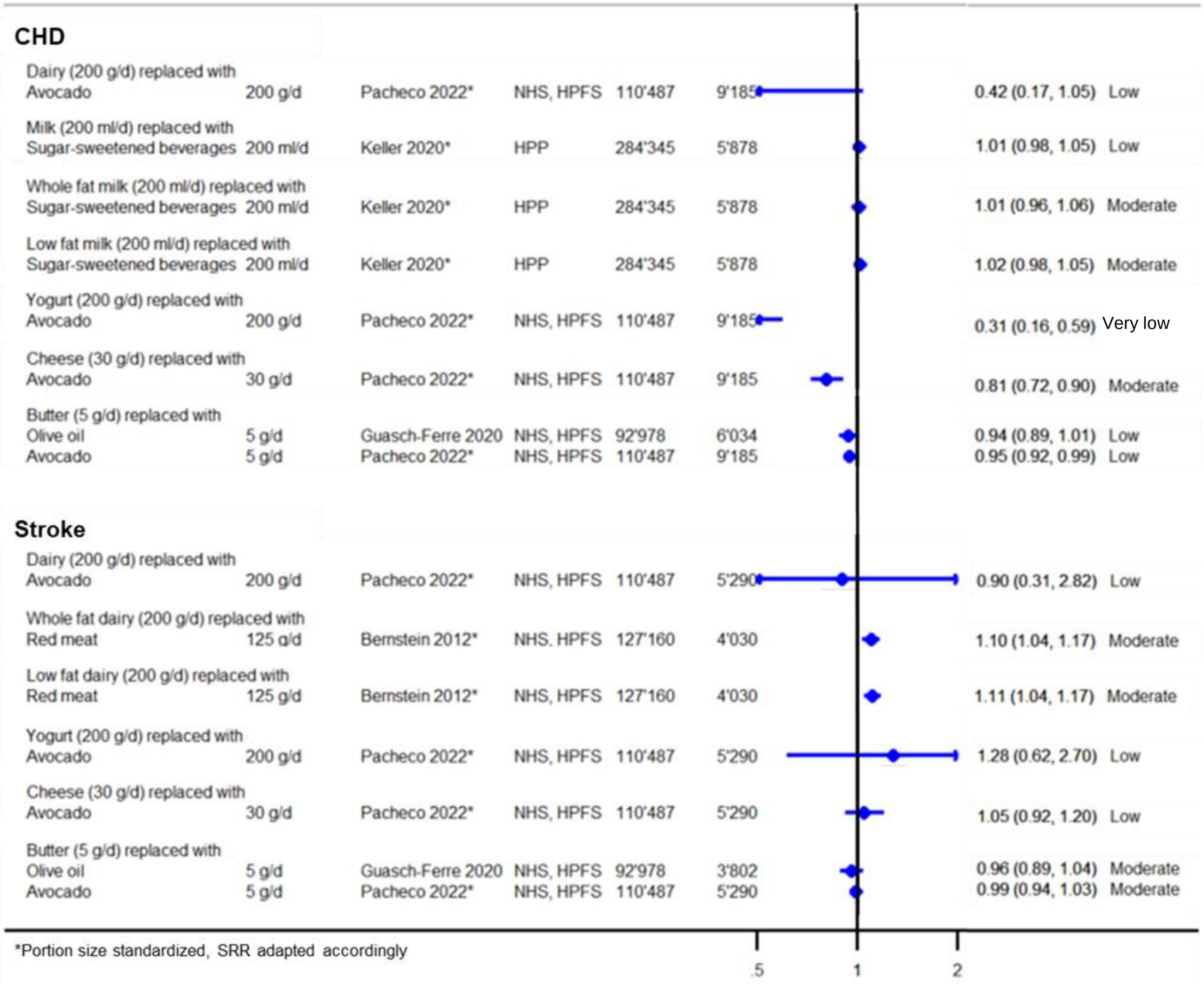

*Portion size standardized, SRR adapted accordingly

Supplementary figure 13: Extracted pooled results for coronary artery disease (fatal and non-fatal), and stroke (fatal-non-fatal) (replacement and substitution are used synonymously)

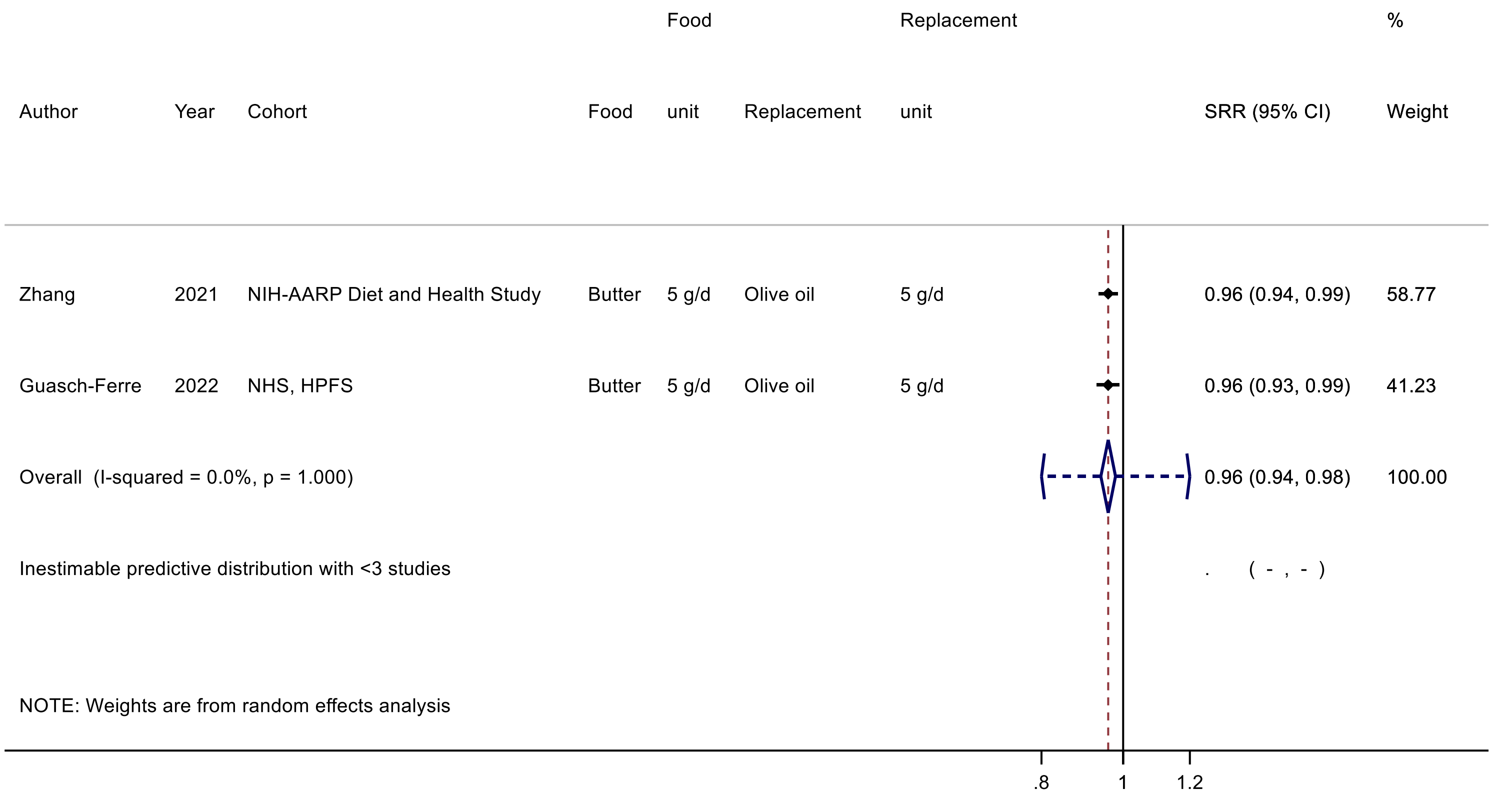

Supplementary figure 14: Meta-analysis comparing the substituting effect of substituting butter with an equal amount of olive oil on cardiovascular disease risk (replacement and substitution are used synonymously)

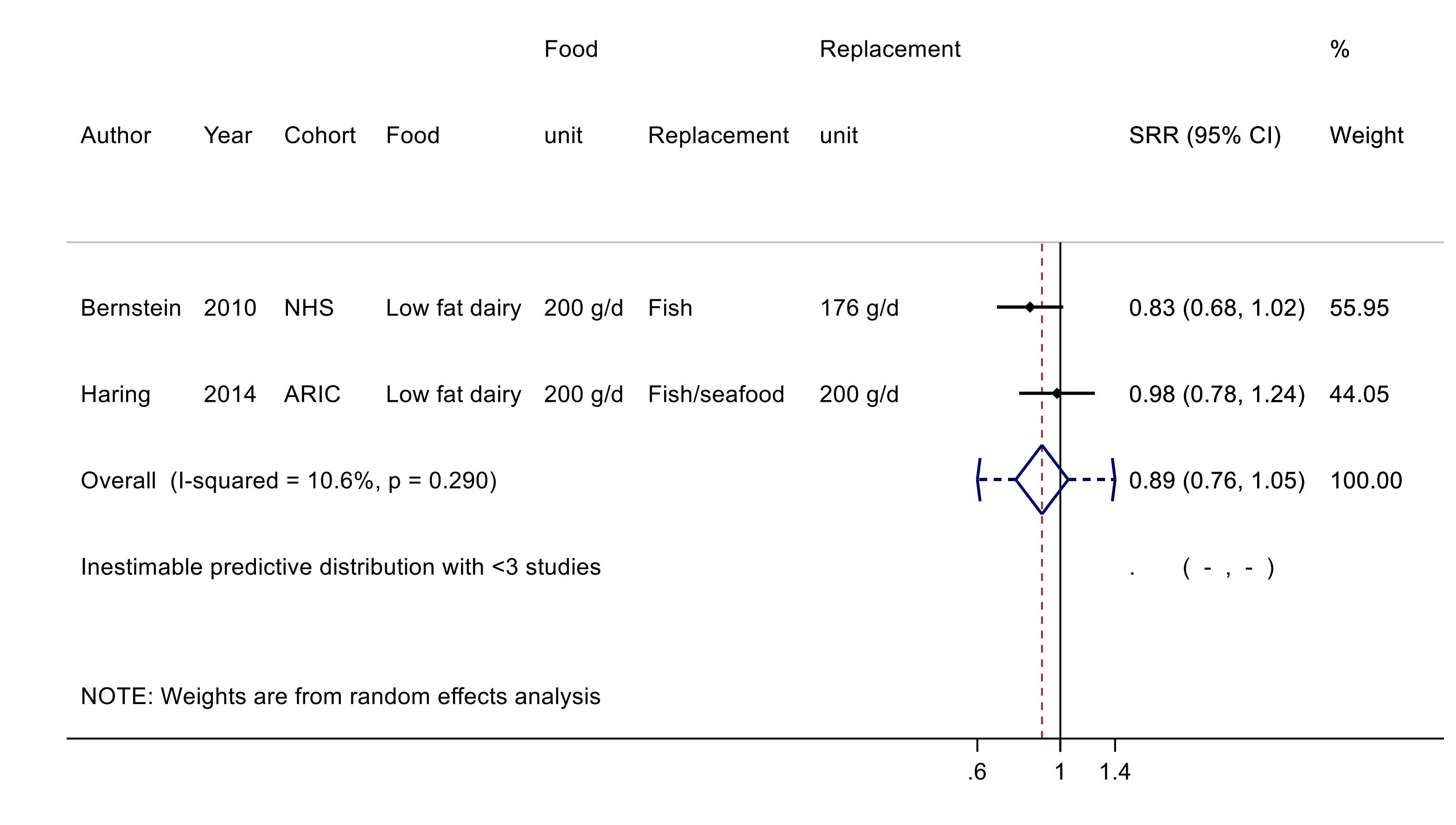

Supplementary figure 15: Meta-analysis comparing the substituting effect of substituting low-fat dairy with fish/seafood on coronary artery disease risk (replacement and substitution are used synonymously)

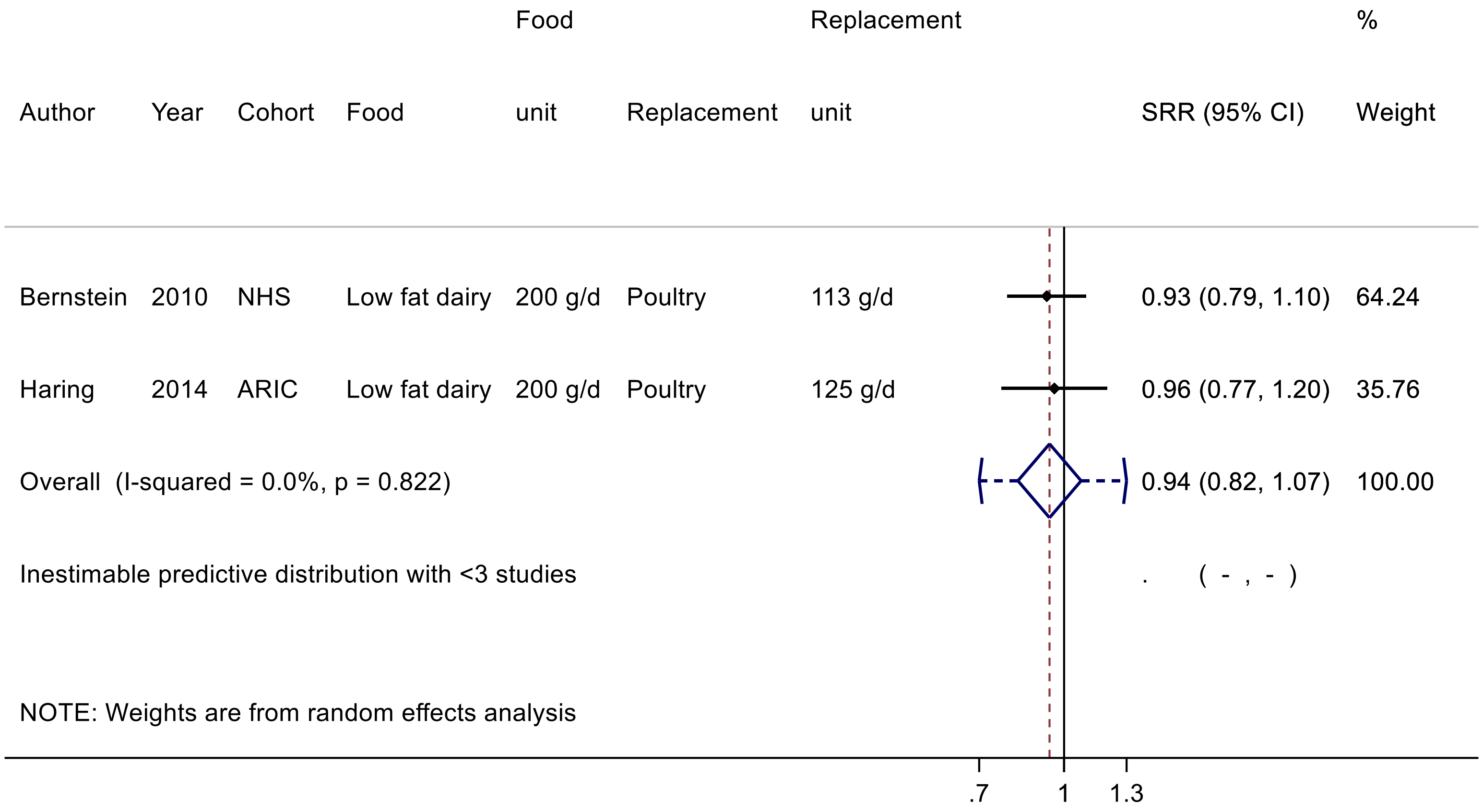

Supplementary figure 16: Meta-analysis comparing the substituting effect of substituting low-fat dairy with poultry on coronary artery disease risk (replacement and substitution are used synonymously)

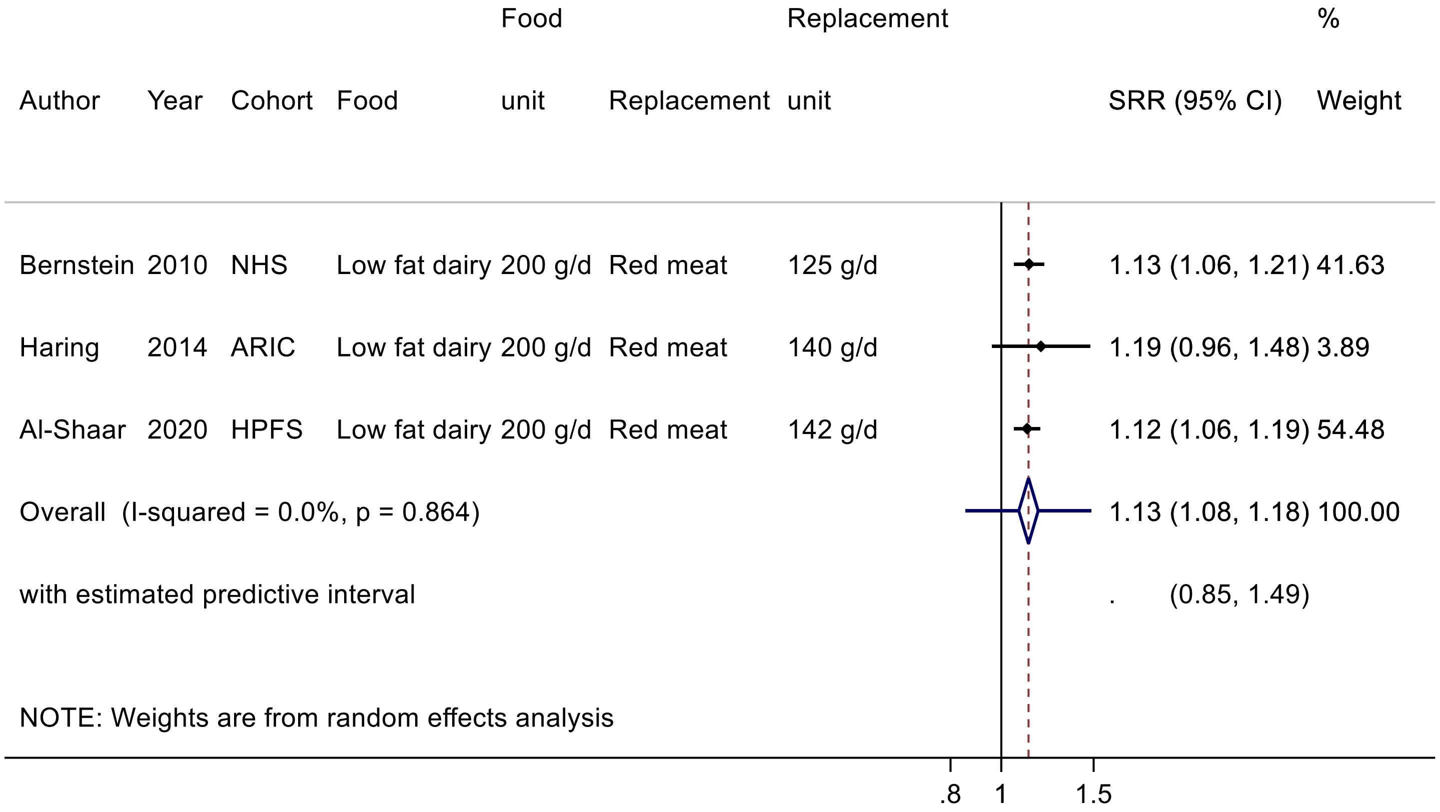

Supplementary figure 17: Meta-analysis comparing the substituting effect of substituting low-fat dairy with red meat on coronary artery disease risk (replacement and substitution are used synonymously)

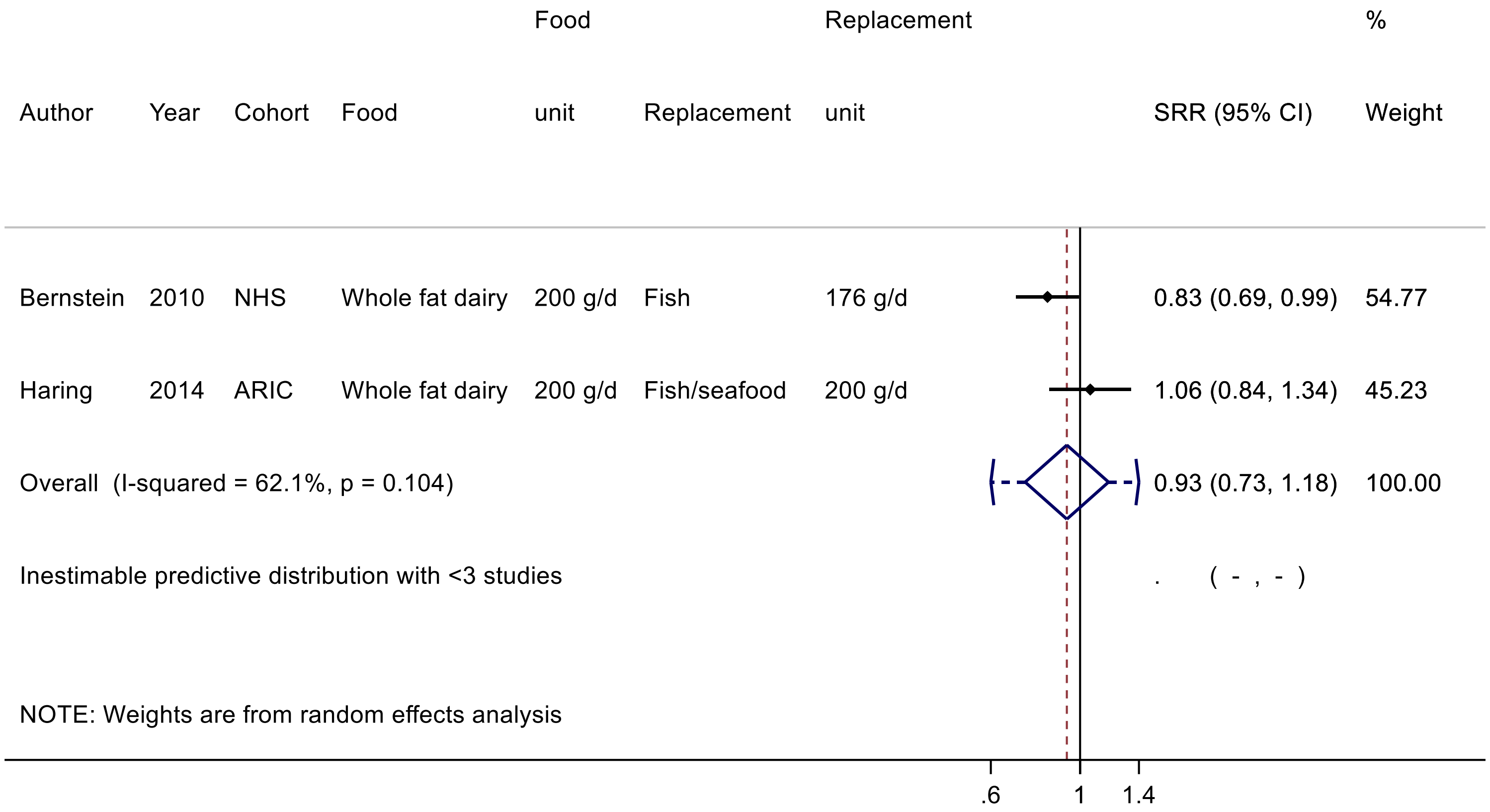

Supplementary figure 18: Meta-analysis comparing the substituting effect of substituting whole-fat dairy with fish/seafood on coronary artery disease risk (replacement and substitution are used synonymously)

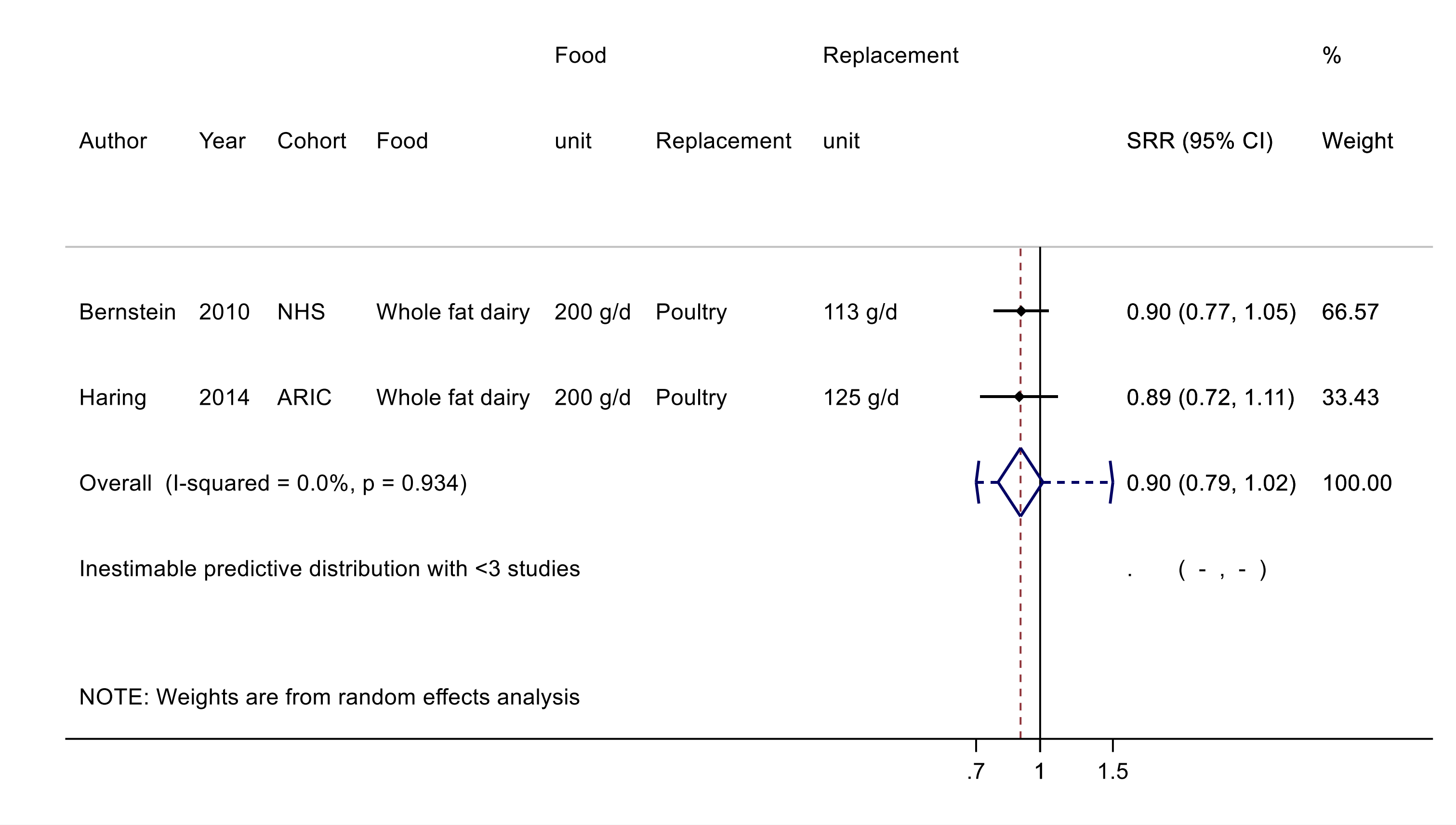

Supplementary figure 19: Meta-analysis comparing the substituting effect of substituting whole-fat dairy with poultry on coronary artery disease risk (replacement and substitution are used synonymously)

Author	Year	Cohort	Food	Food unit	Replacement	Replacement unit	SRR (95% CI)	% Weight
Bernstein	2010	NHS	Whole fat dairy	200 g/d	Red meat	125 g/d	1.11 (1.05, 1.18)	56.12
Haring	2014	ARIC	Whole fat dairy	200 g/d	Red meat	140 g/d	1.10 (0.89, 1.35)	4.49
Al-Shaar	2020	HPFS	Whole fat dairy	200 g/d	Red meat	142 g/d	1.14 (1.06, 1.22)	39.39
Overall (I-squared = 0.0%, p = 0.836)							1.12 (1.07, 1.17)	100.00
with estimated predictive interval							. (0.84, 1.49)	

NOTE: Weights are from random effects analysis

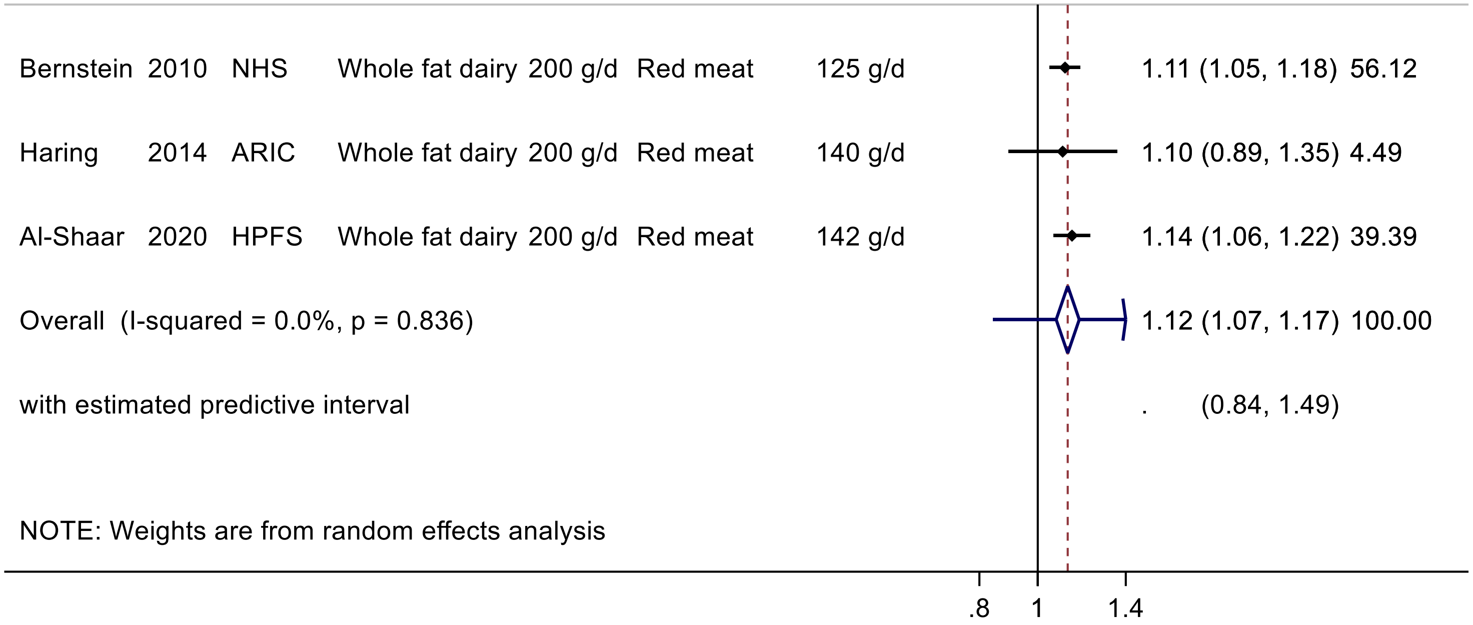

Supplementary figure 20: Meta-analysis comparing the substituting effect of substituting whole-fat dairy with red meat on coronary artery disease risk (replacement and substitution are used synonymously)

Author	Year	Cohort	Food	Food unit	Replacement	Replacement unit	SRR (95% CI)	% Weight
Laursen	2018	DCH	Butter	5 g/d	Cheese	17 g/d	0.97 (0.94, 1.01)	78.88
Laursen	2019	EPIC-NL	Butter	5 g/d	Cheese	20 g/d	1.00 (0.93, 1.07)	21.12
Overall (I-squared = 0.0%, p = 0.450)							0.98 (0.95, 1.01)	100.00
Inestimable predictive distribution with <3 studies							. (- , -)	

NOTE: Weights are from random effects analysis

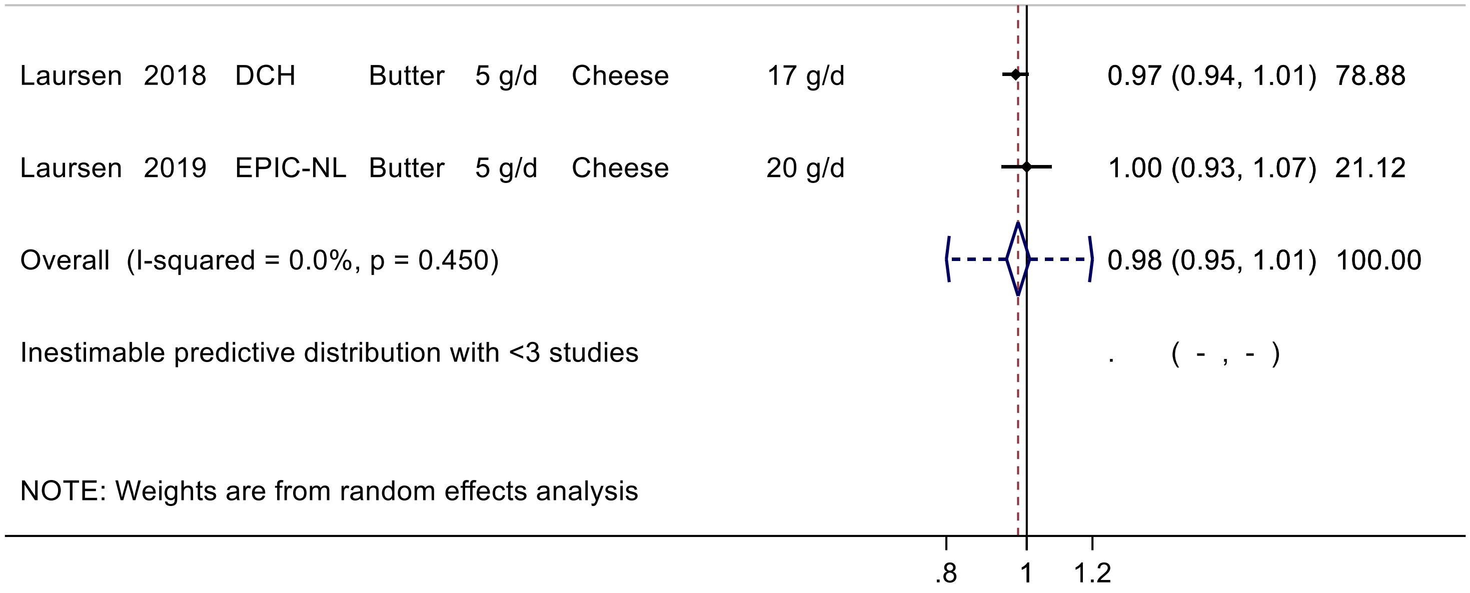

Supplementary figure 21: Meta-analysis comparing the substituting effect of substituting butter with cheese on stroke risk (replacement and substitution are used synonymously)

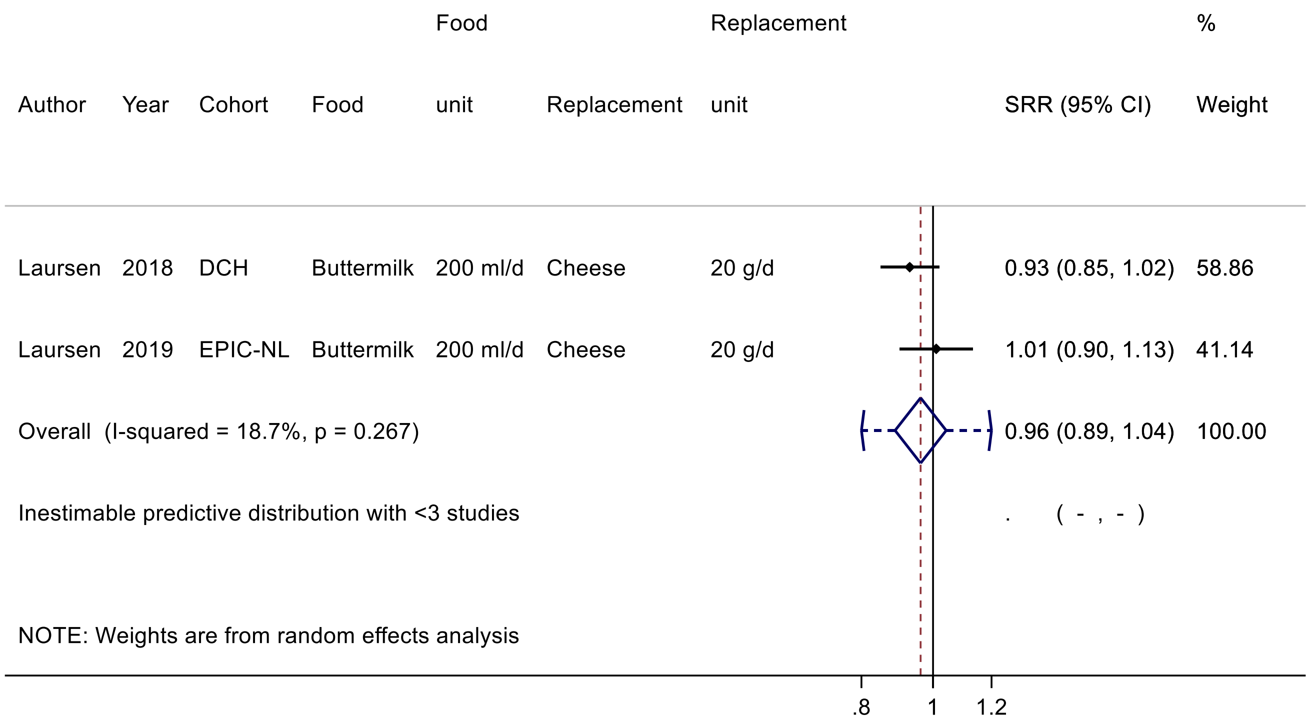

Supplementary figure 22: Meta-analysis comparing the substituting effect of substituting buttermilk with cheese on stroke risk (replacement and substitution are used synonymously)

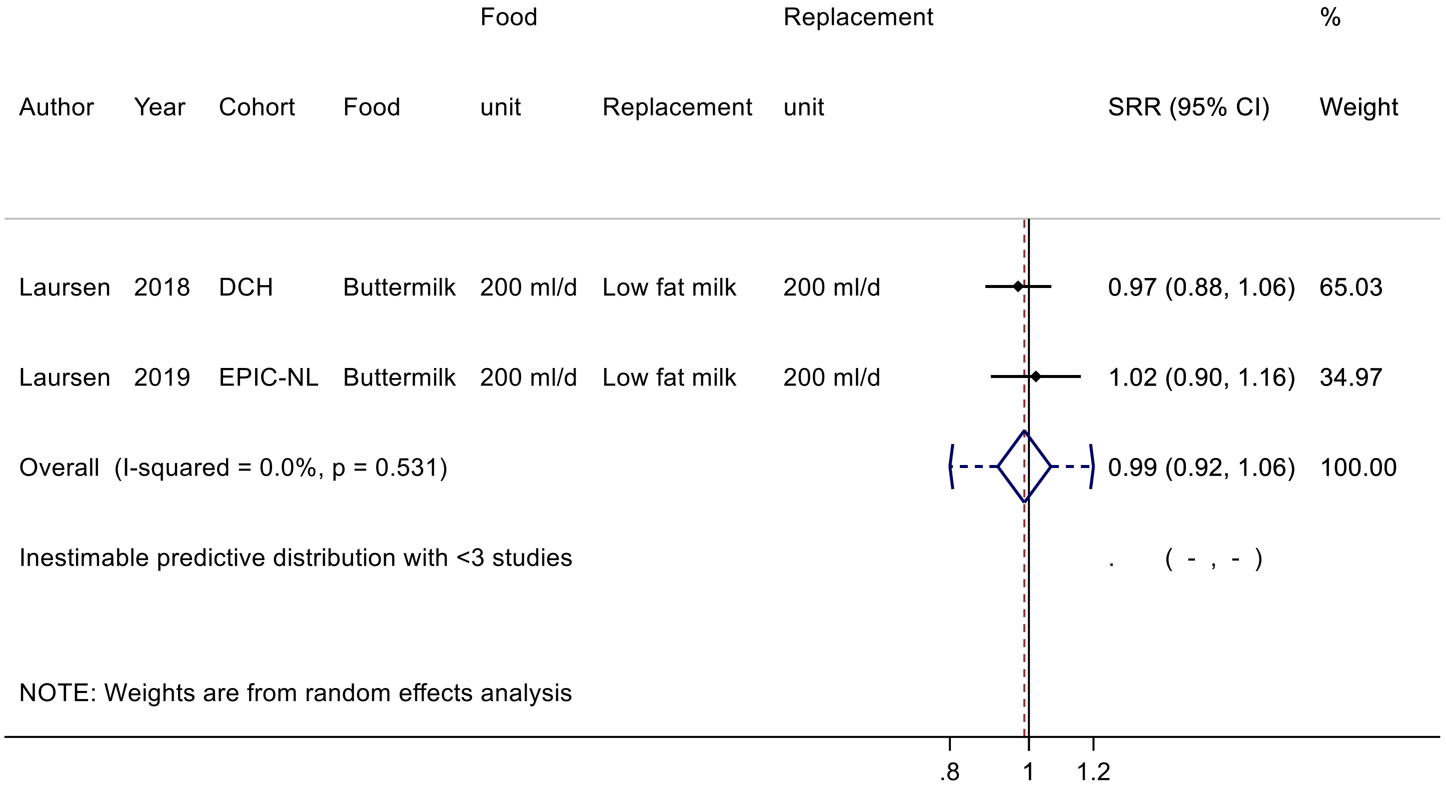

Supplementary figure 23: Meta-analysis comparing the substituting effect of substituting buttermilk with an equal amount of low-fat milk on stroke risk (replacement and substitution are used synonymously)

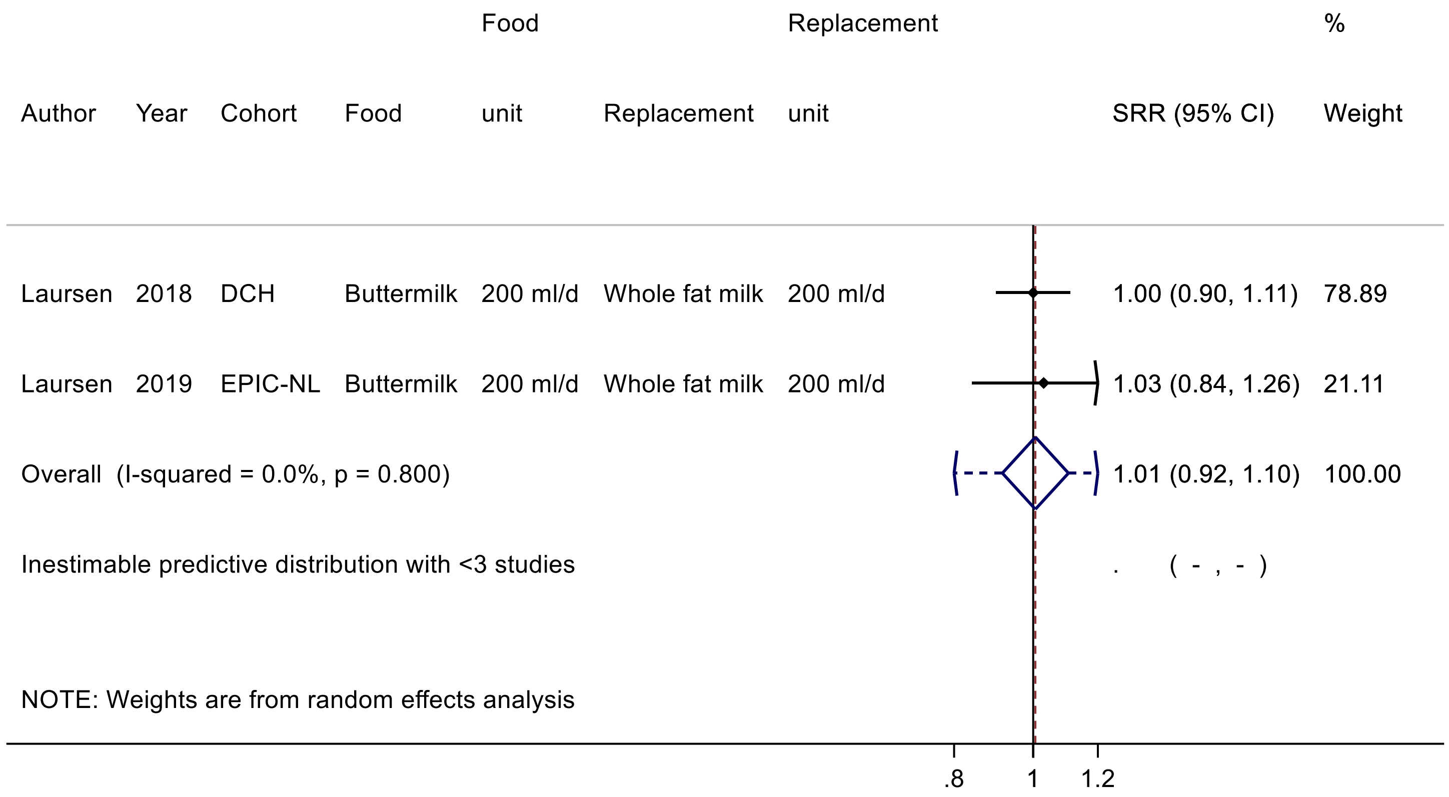

Supplementary figure 24: Meta-analysis comparing the substituting effect of substituting buttermilk with an equal amount of whole-fat milk on stroke risk (replacement and substitution are used synonymously)

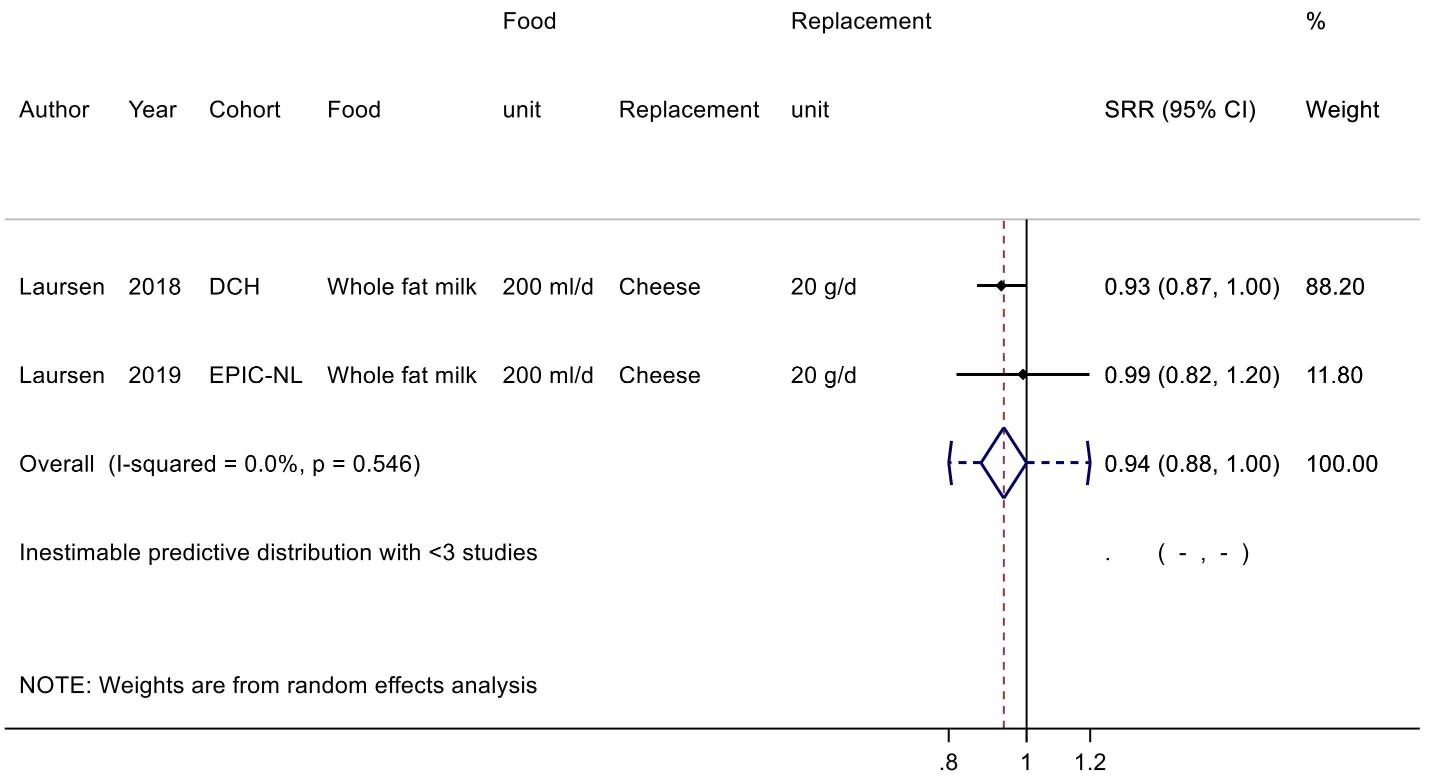

Supplementary figure 25: Meta-analysis comparing the substituting effect of substituting whole-fat milk with cheese on stroke risk (replacement and substitution are used synonymously)

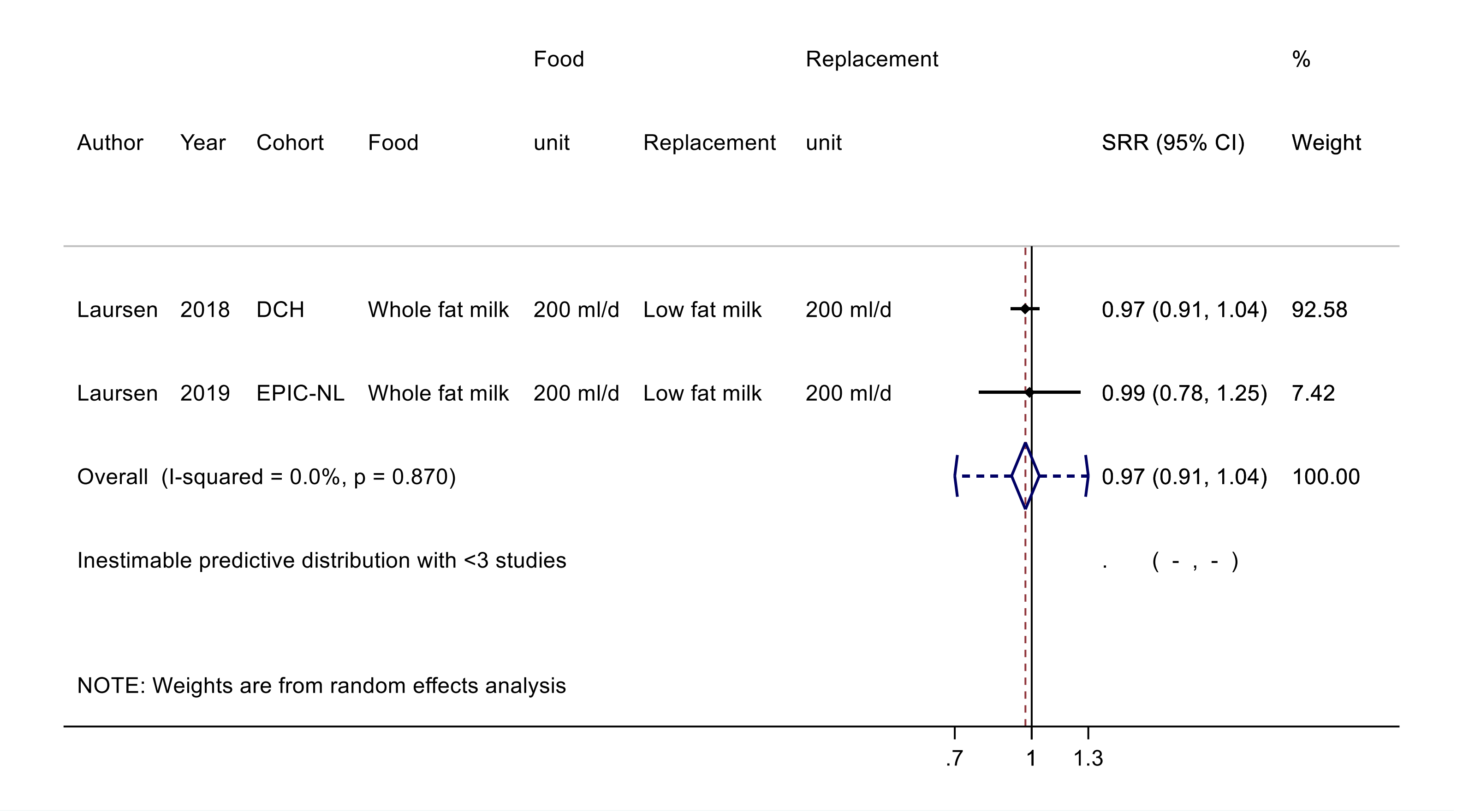

Supplementary figure 26: Meta-analysis comparing the substituting effect of substituting whole-fat milk with an equal amount of low-fat milk on stroke risk (replacement and substitution are used synonymously)

Replacement	Replacement unit	Study	Cohort	No of participants	No of cases	Summary risk ratio (95% CI)	Certainty of Evidence
**Type 2 diabetes incidence**							
Dairy (200 g/d) replaced with							
Whole grains	30 g/d	Malik 2016	NHS, NHS II, HPFS	205'802	15'580	0.97 (0.95, 1.00)	Moderate
Peanuts and peanut butter	28 g/d	Malik 2016	NHS, NHS II, HPFS	205'802	15'580	0.98 (0.93, 1.03)	Moderate
Low fat dairy (200 g/d) replaced with							
Red meat	140 g/d	Pan 2011	NHS, NHS II, HPFS	204'157	13'759	1.20 (1.16, 1.25)	Moderate
Processed red meat	50 g/d	Pan 2011	NHS, NHS II, HPFS	204'157	13'759	1.41 (1.33, 1.49)	Moderate
Unprocessed red meat	85 g/d	Pan 2011	NHS, NHS II, HPFS	204'157	13'759	1.19 (1.15, 1.23)	Moderate
Butter (5 g/d) replaced with							
Olive oil	5 g/d	Guasch-Ferre 2015*	NHS, NHS II	145'087	9'652	0.95 (0.92, 0.98)	Low

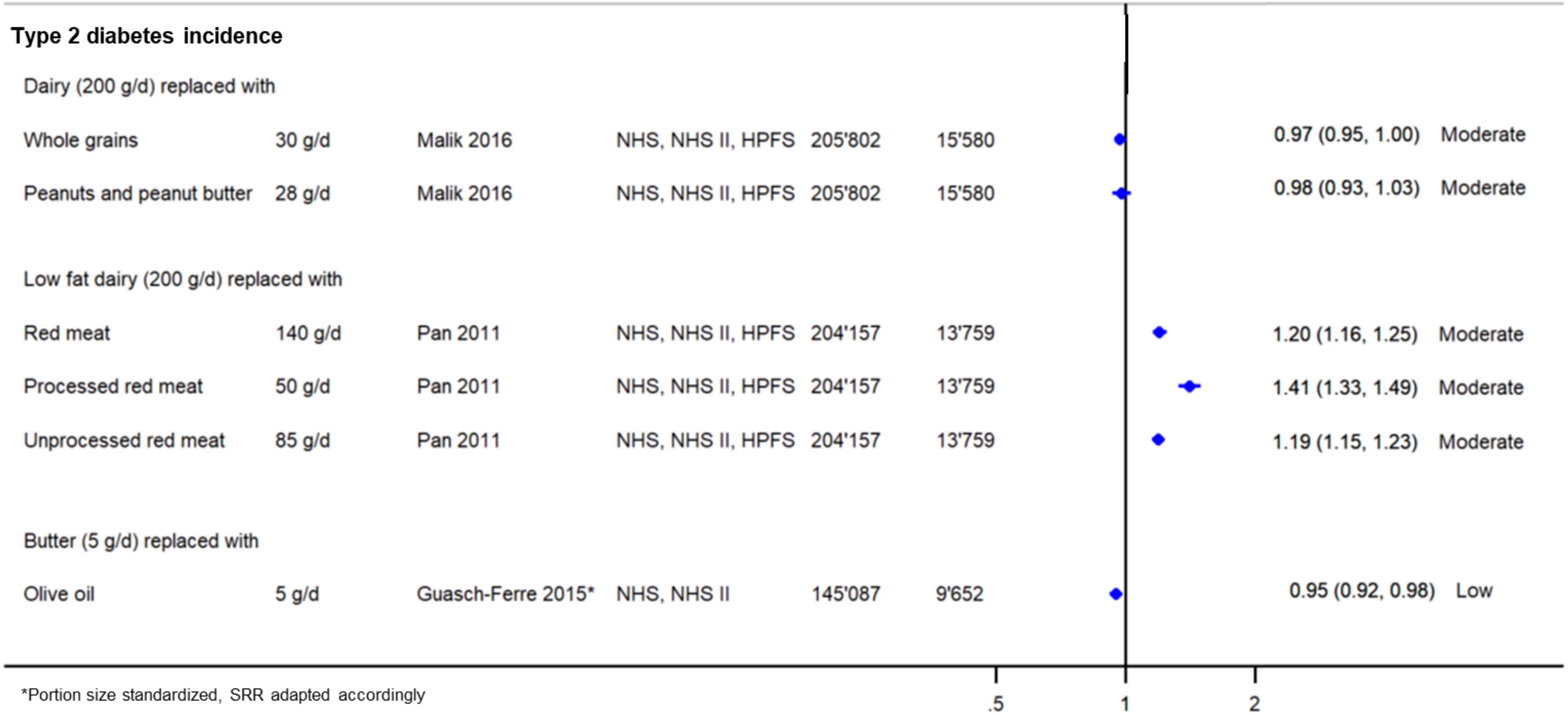

*Portion size standardized, SRR adapted accordingly

Supplementary figure 27: Extracted pooled results for type 2 diabetes risk (replacement and substitution are used synonymously)

Author	Year	Cohort	Food	Food unit	Replacement	Replacement unit	SRR (95% CI)	% Weight
Ibsen	2017	DCH	Buttermilk	200 ml/d	Low fat milk	200 ml/d	0.99 (0.94, 1.05)	75.35
Stuber	2021	EPIC-NL	Buttermilk	200 ml/d	Low fat milk	200 ml/d	0.98 (0.89, 1.08)	24.65
Overall (I-squared = 0.0%, p = 0.858)							0.99 (0.94, 1.04)	100.00
Inestimable predictive distribution with <3 studies							. (- , -)	

NOTE: Weights are from random effects analysis

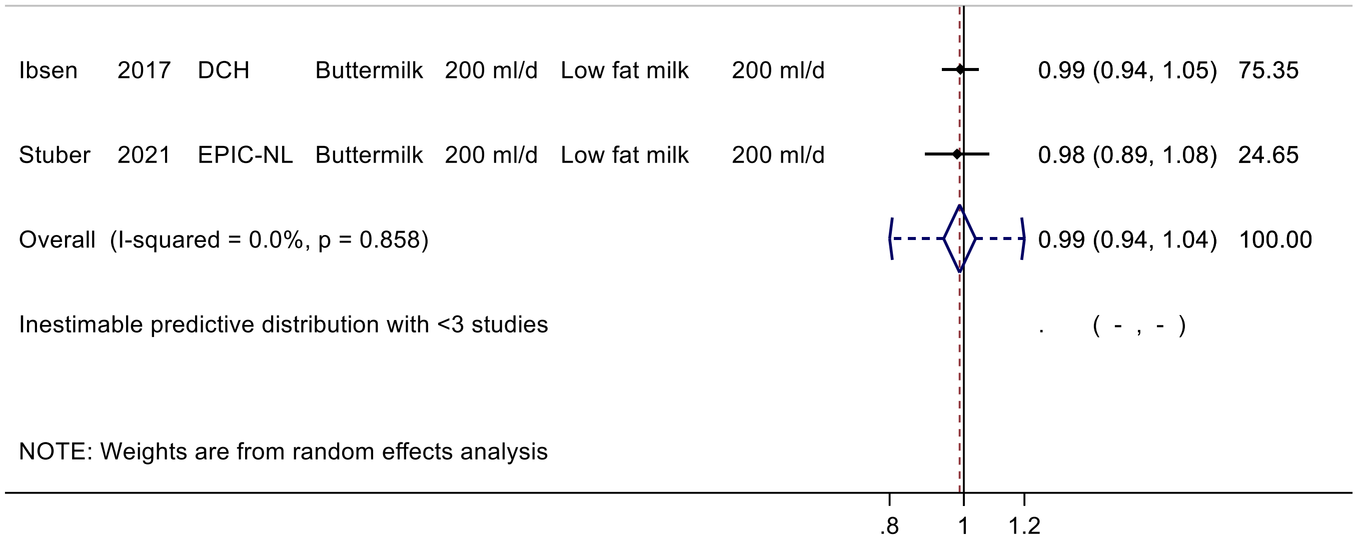

Supplementary figure 28: Meta-analysis comparing the substituting effect of substituting buttermilk with an equal amount of low-fat milk on type 2 diabetes risk (replacement and substitution are used synonymously)

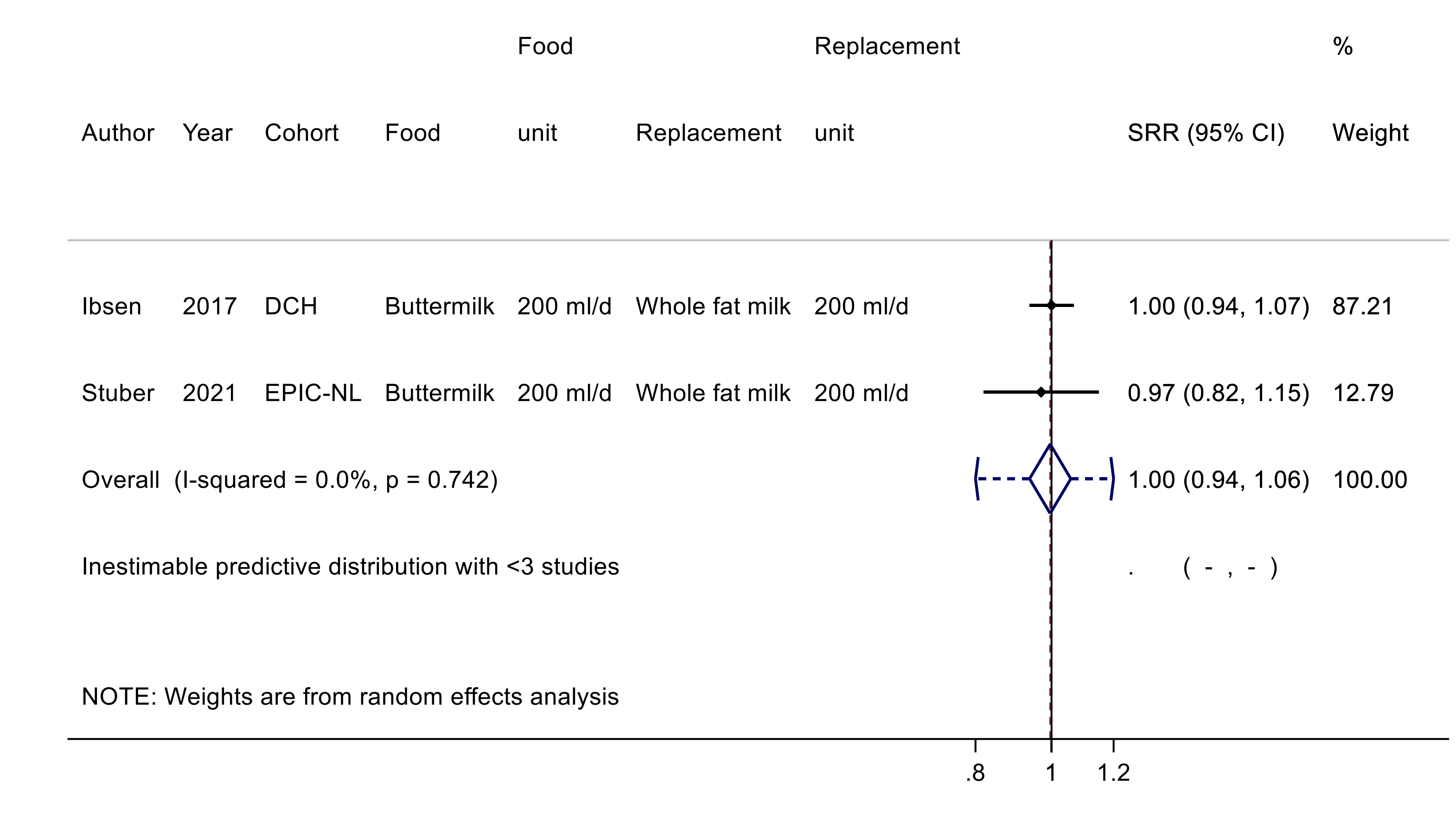

Supplementary figure 29: Meta-analysis comparing the substituting effect of substituting buttermilk with an equal amount of whole-fat milk on type 2 diabetes risk (replacement and substitution are used synonymously)

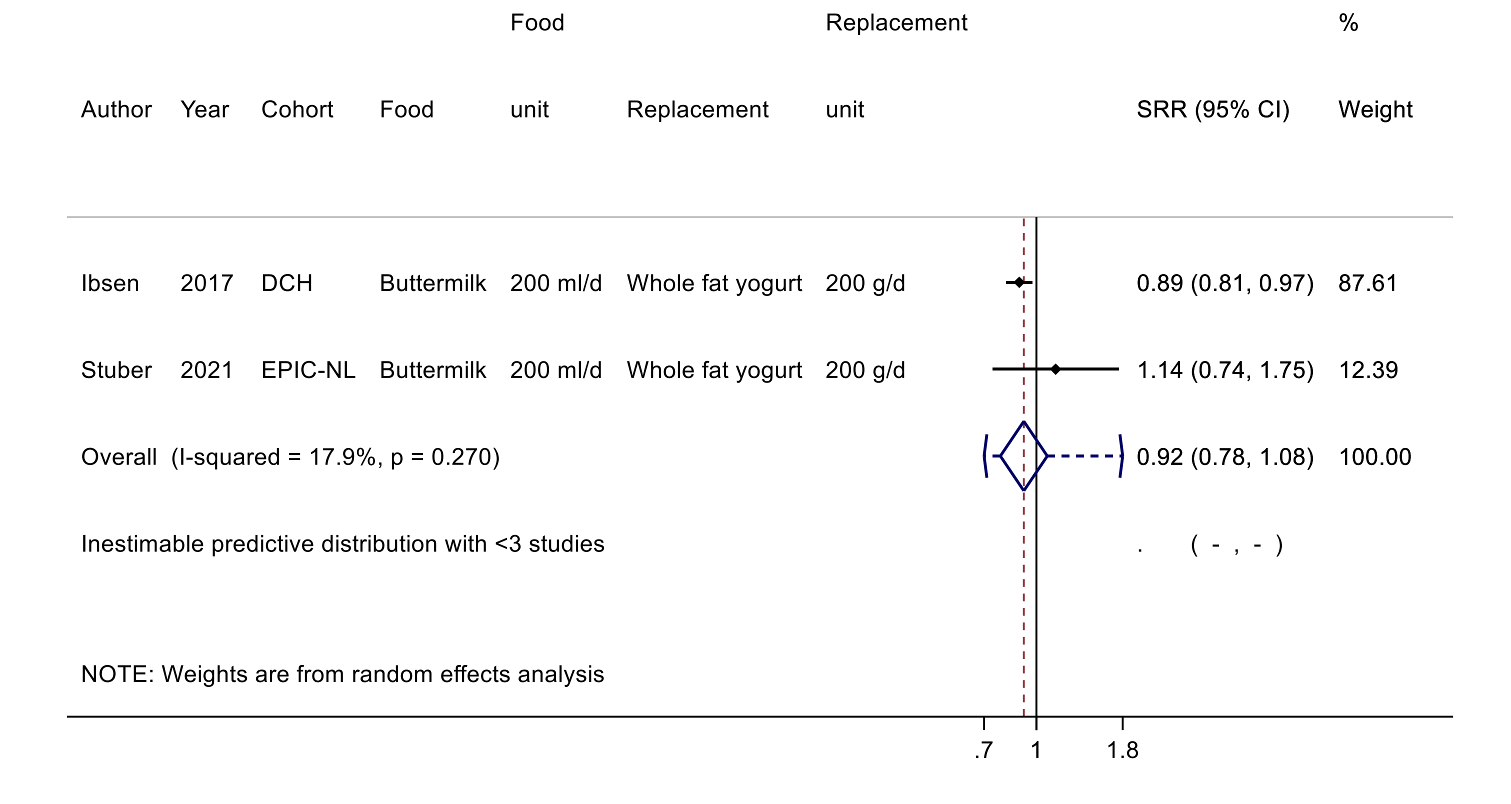

Supplementary figure 30: Meta-analysis comparing the substituting effect of substituting buttermilk with an equal amount of whole-fat yogurt on type 2 diabetes risk (replacement and substitution are used synonymously)

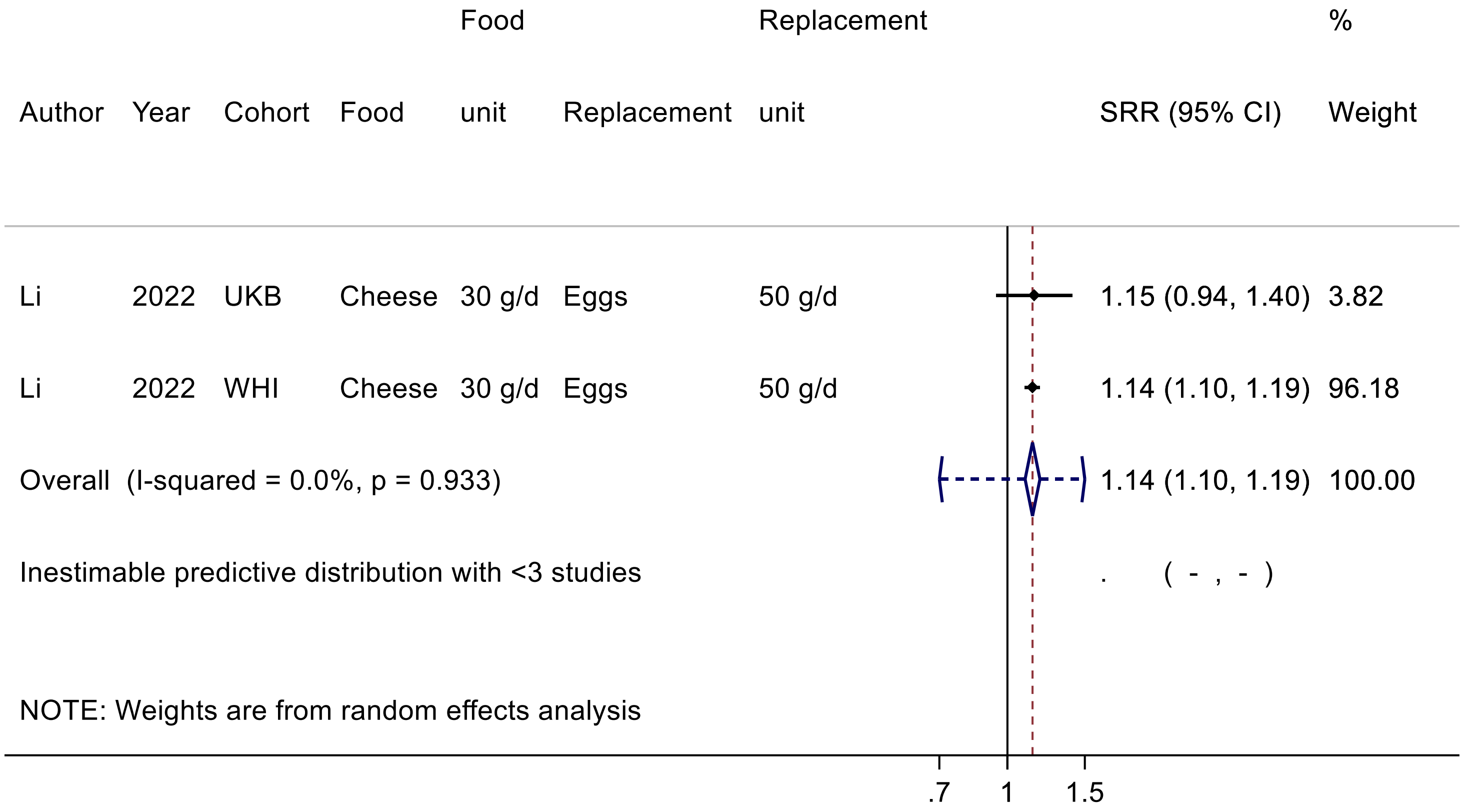

Supplementary figure 31: Meta-analysis comparing the substituting effect of substituting cheese with eggs on type 2 diabetes risk (replacement and substitution are used synonymously)

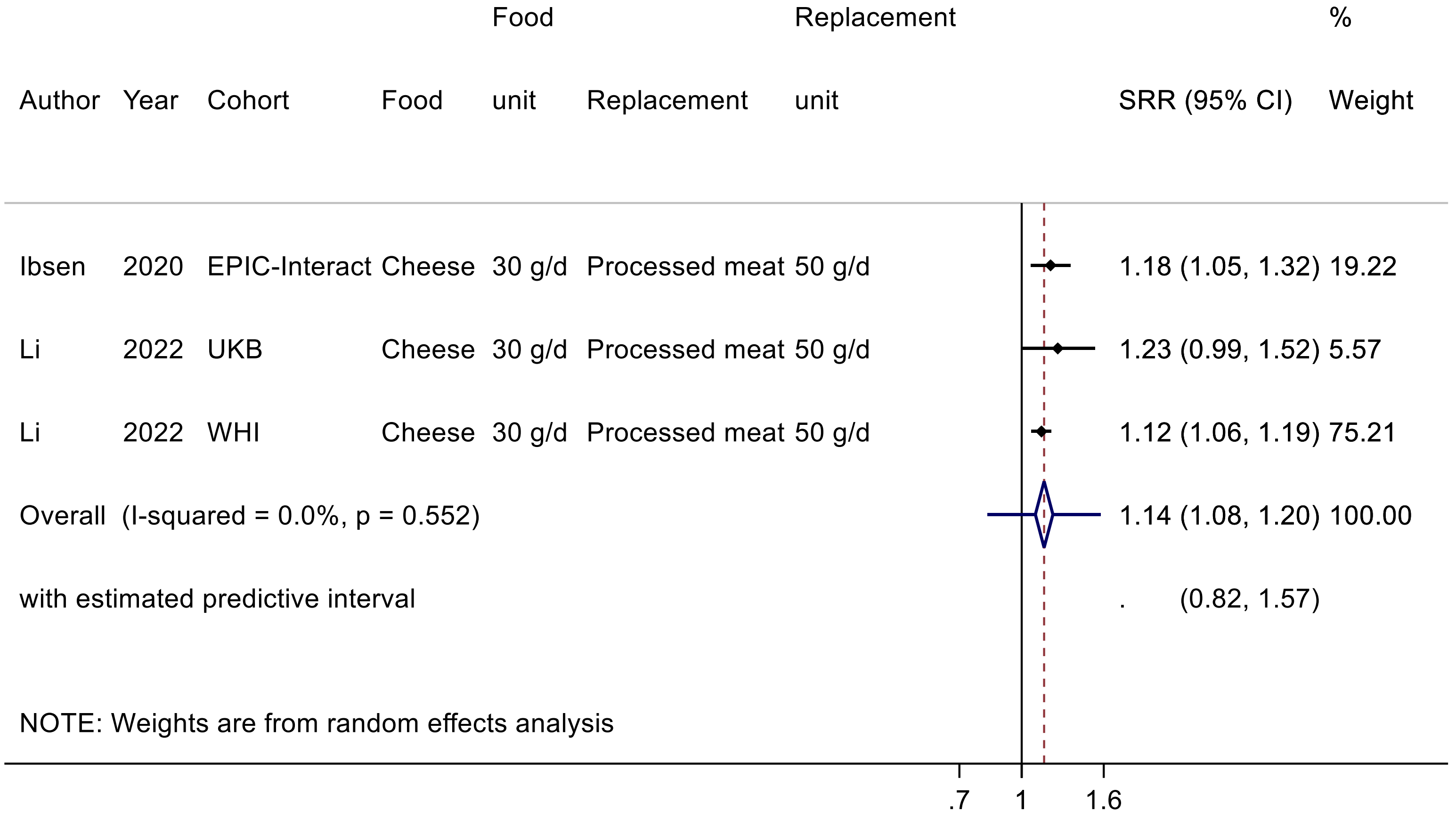

Supplementary figure 32: Meta-analysis comparing the substituting effect of substituting cheese with processed meat on type 2 diabetes risk (replacement and substitution are used synonymously)

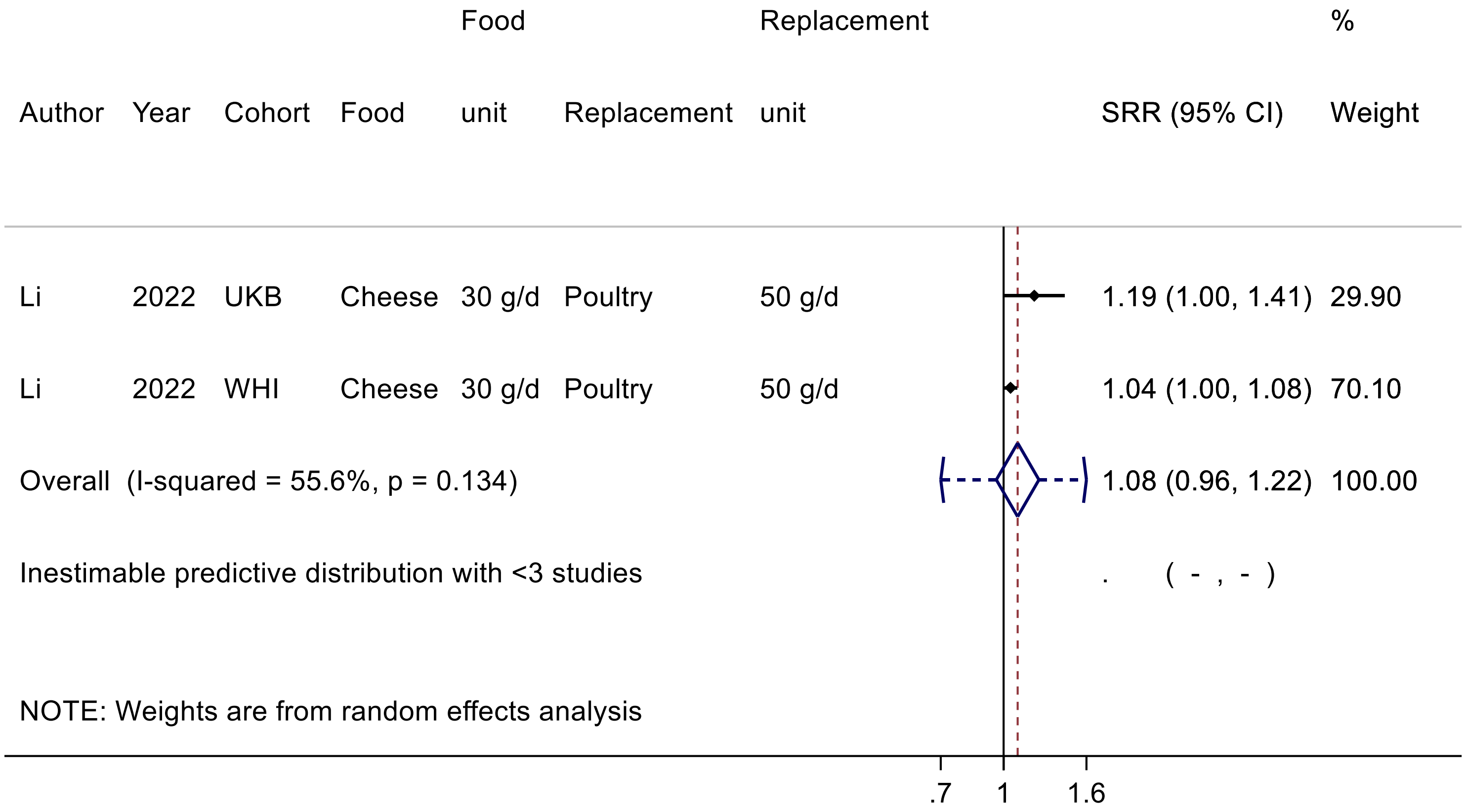

Supplementary figure 33: Meta-analysis comparing the substituting effect of substituting cheese with poultry on type 2 diabetes risk (replacement and substitution are used synonymously)

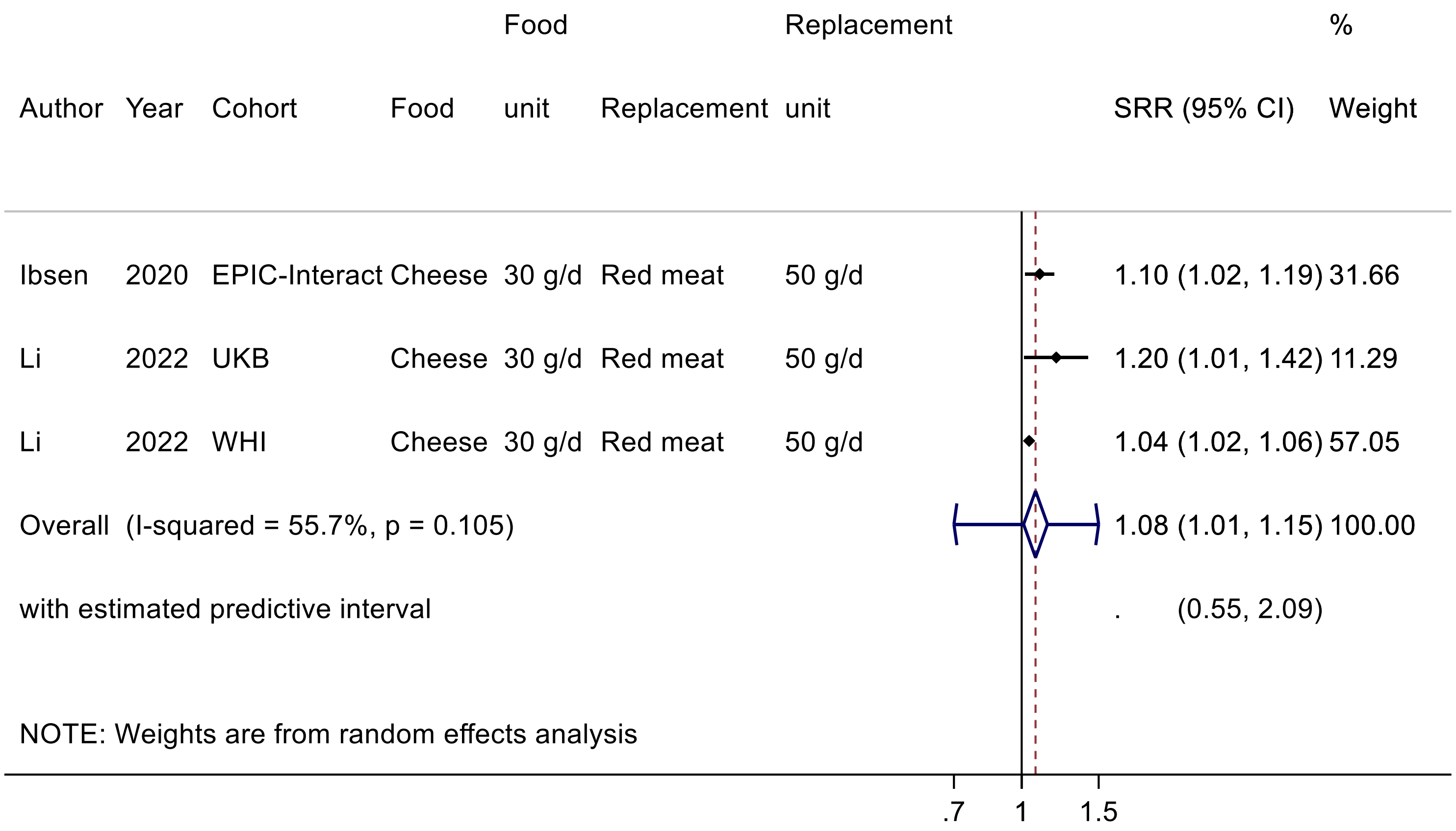

Supplementary figure 34: Meta-analysis comparing the substituting effect of substituting cheese with red meat on type 2 diabetes risk
(replacement and substitution are used synonymously)

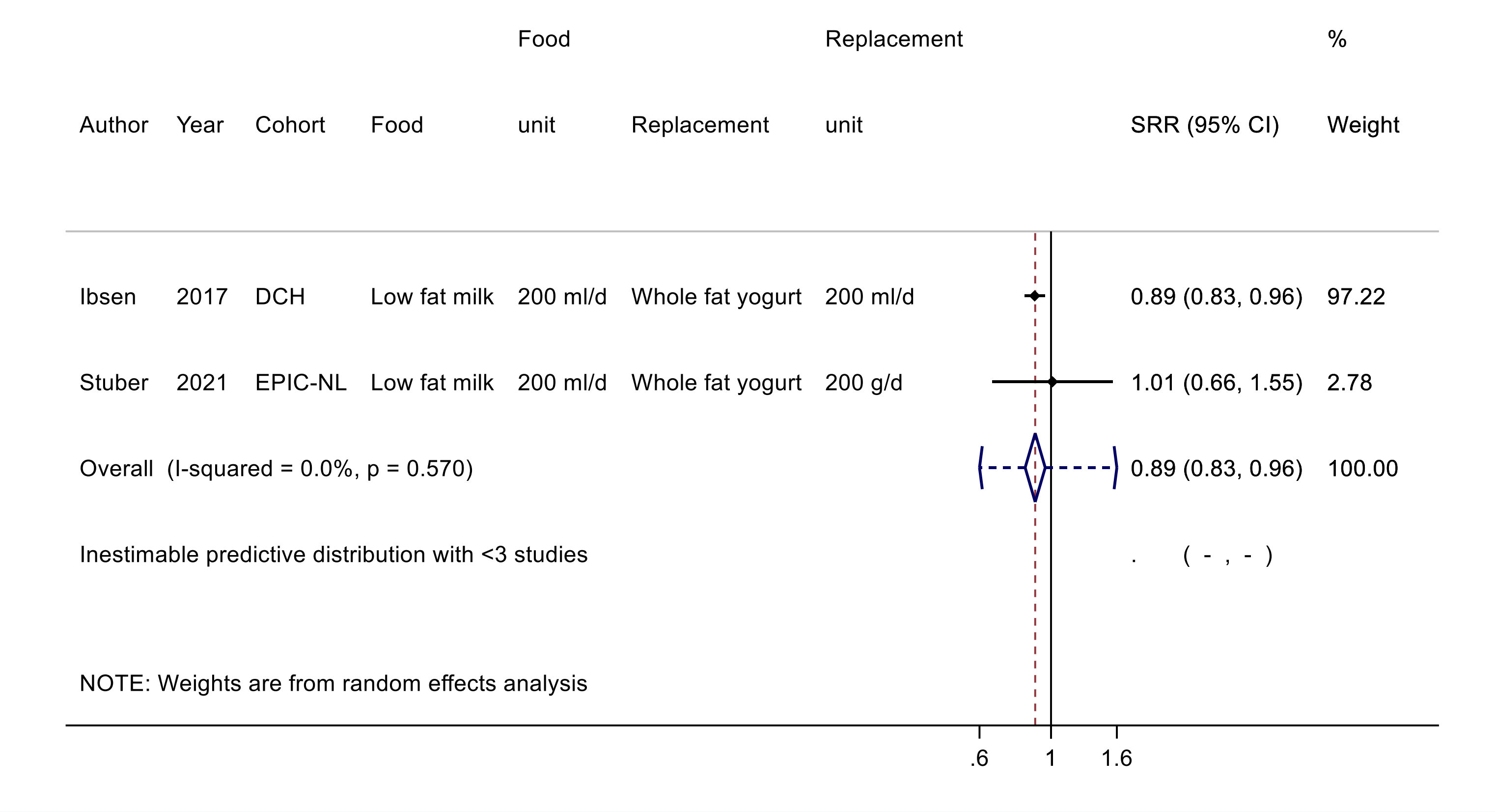

Supplementary figure 35: Meta-analysis comparing the substituting effect of substituting low-fat milk with an equal amount of whole-fat yogurt on type 2 diabetes risk (replacement and substitution are used synonymously)

Author	Year	Cohort	Food	Food unit	Replacement	Replacement unit	SRR (95% CI)	% Weight
Li	2022	WHI	Milk	200 ml/d	Eggs	50 g/d	1.14 (1.10, 1.19)	55.30
Li	2022	UKB	Milk	200 ml/d	Eggs	50 g/d	0.87 (0.73, 1.04)	44.70
Overall (I-squared = 88.3%, p = 0.003)							1.01 (0.78, 1.31)	100.00
Inestimable predictive distribution with <3 studies							. (- , -)	

NOTE: Weights are from random effects analysis

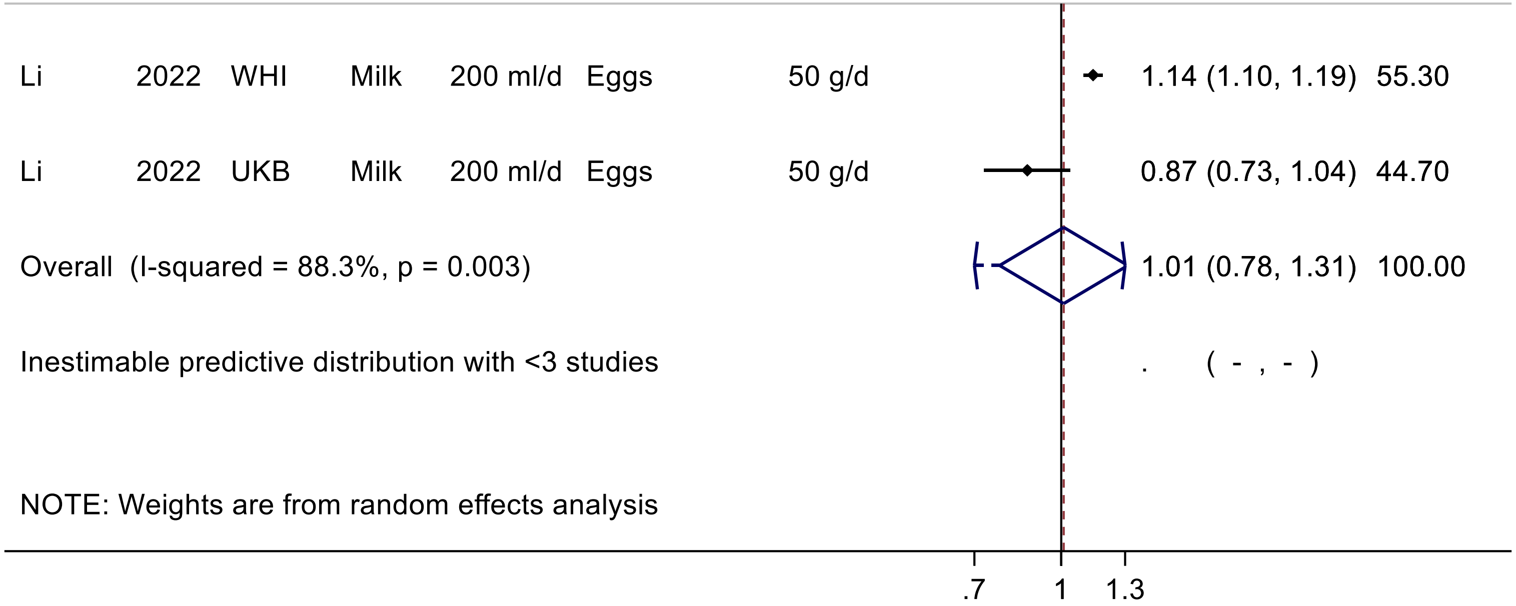

Supplementary figure 36: Meta-analysis comparing the substituting effect of substituting milk with eggs on type 2 diabetes risk
(replacement and substitution are used synonymously)

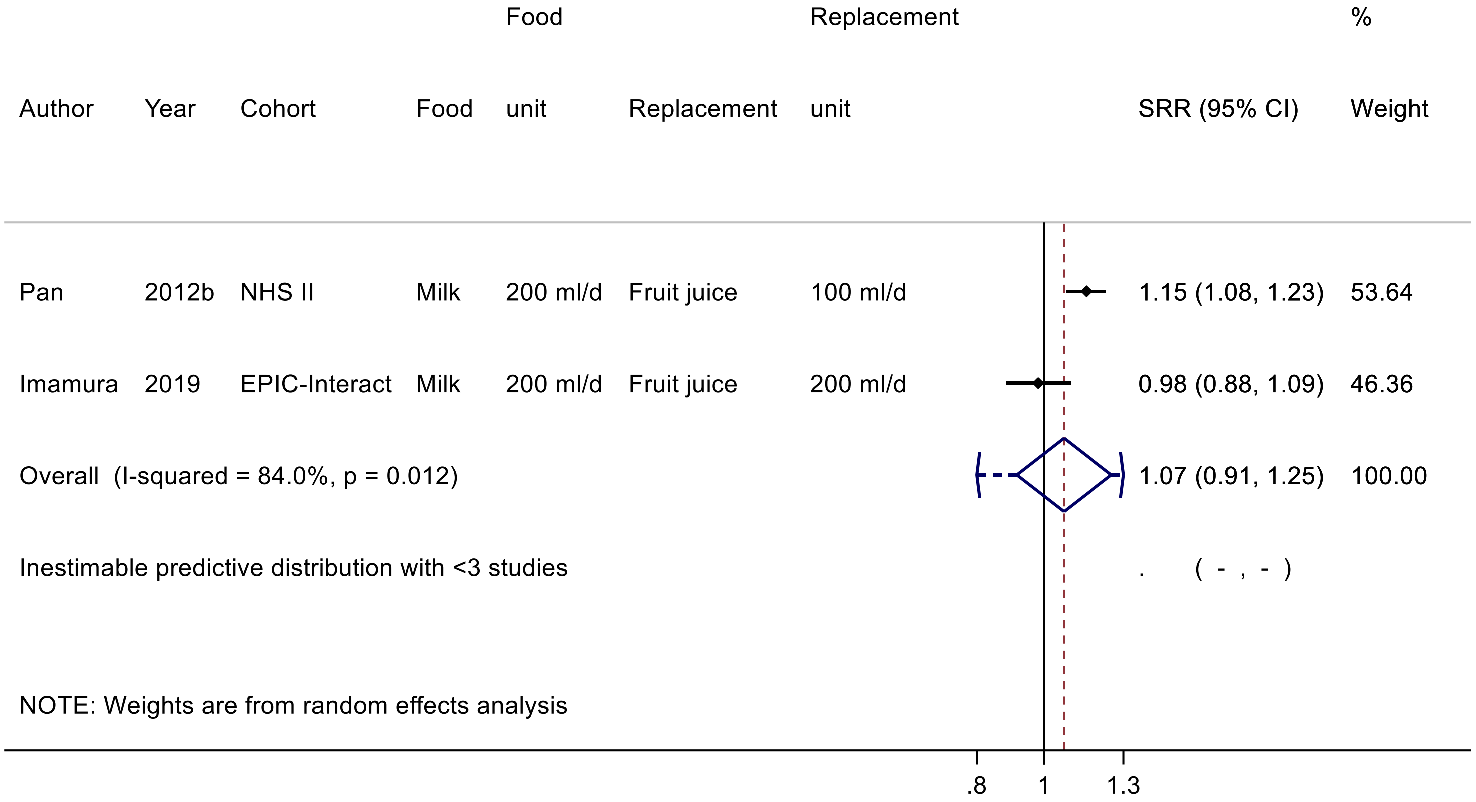

Supplementary figure 37: Meta-analysis comparing the substituting effect of substituting milk with fruit juice on type 2 diabetes risk (replacement and substitution are used synonymously)

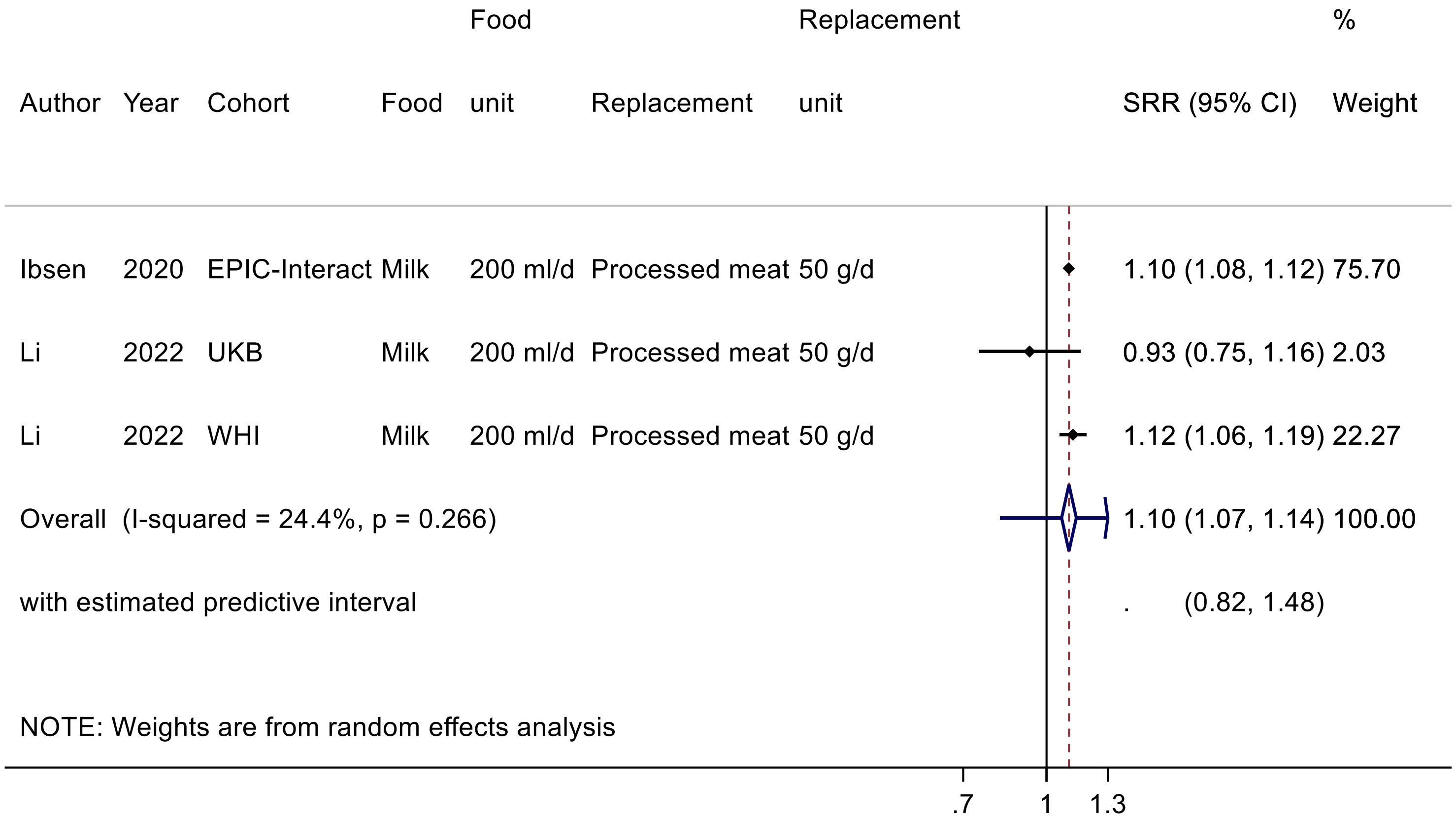

Supplementary figure 38: Meta-analysis comparing the substituting effect of substituting milk with processed meat on type 2 diabetes risk (replacement and substitution are used synonymously)

Author	Year	Cohort	Food	Food unit	Replacement	Replacement unit	SRR (95% CI)	% Weight
Li	2022	WHI	Milk	200 ml/d	Poultry	50 g/d	1.04 (1.00, 1.08)	63.22
Li	2022	UKB	Milk	200 ml/d	Poultry	50 g/d	0.89 (0.76, 1.05)	36.78
Overall (I-squared = 70.4%, p = 0.066)							0.98 (0.85, 1.14)	100.00
Inestimable predictive distribution with <3 studies							. (- , -)	

NOTE: Weights are from random effects analysis

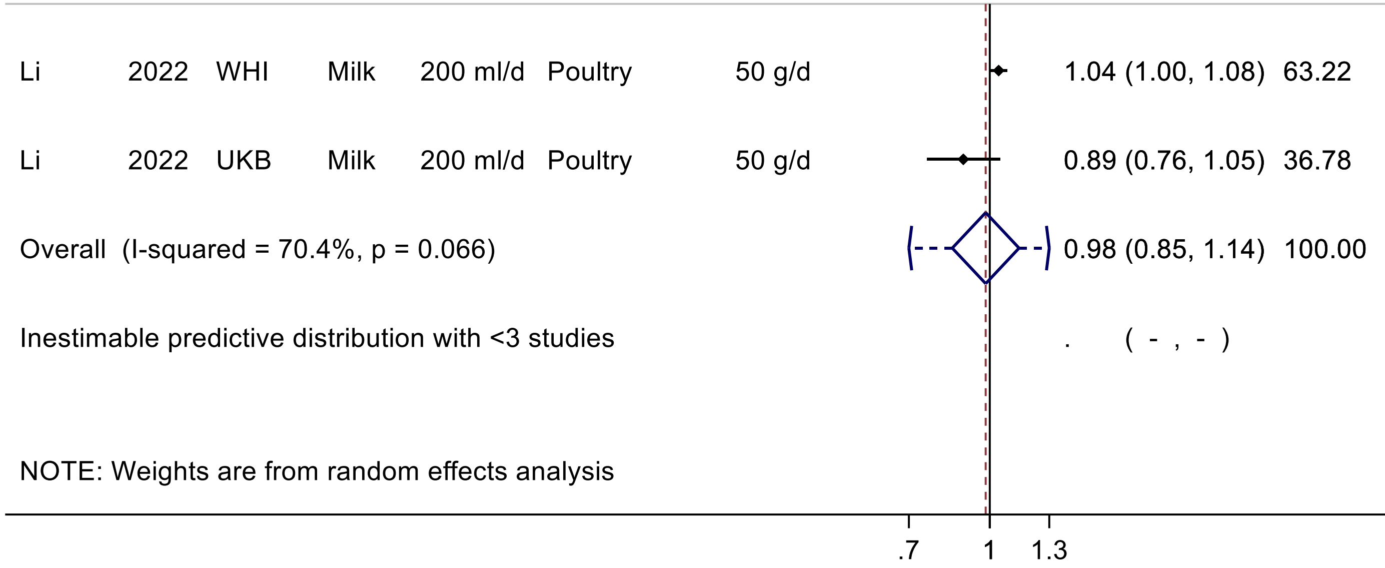

Supplementary figure 39: Meta-analysis comparing the substituting effect of substituting milk with poultry on type 2 diabetes risk
(replacement and substitution are used synonymously)

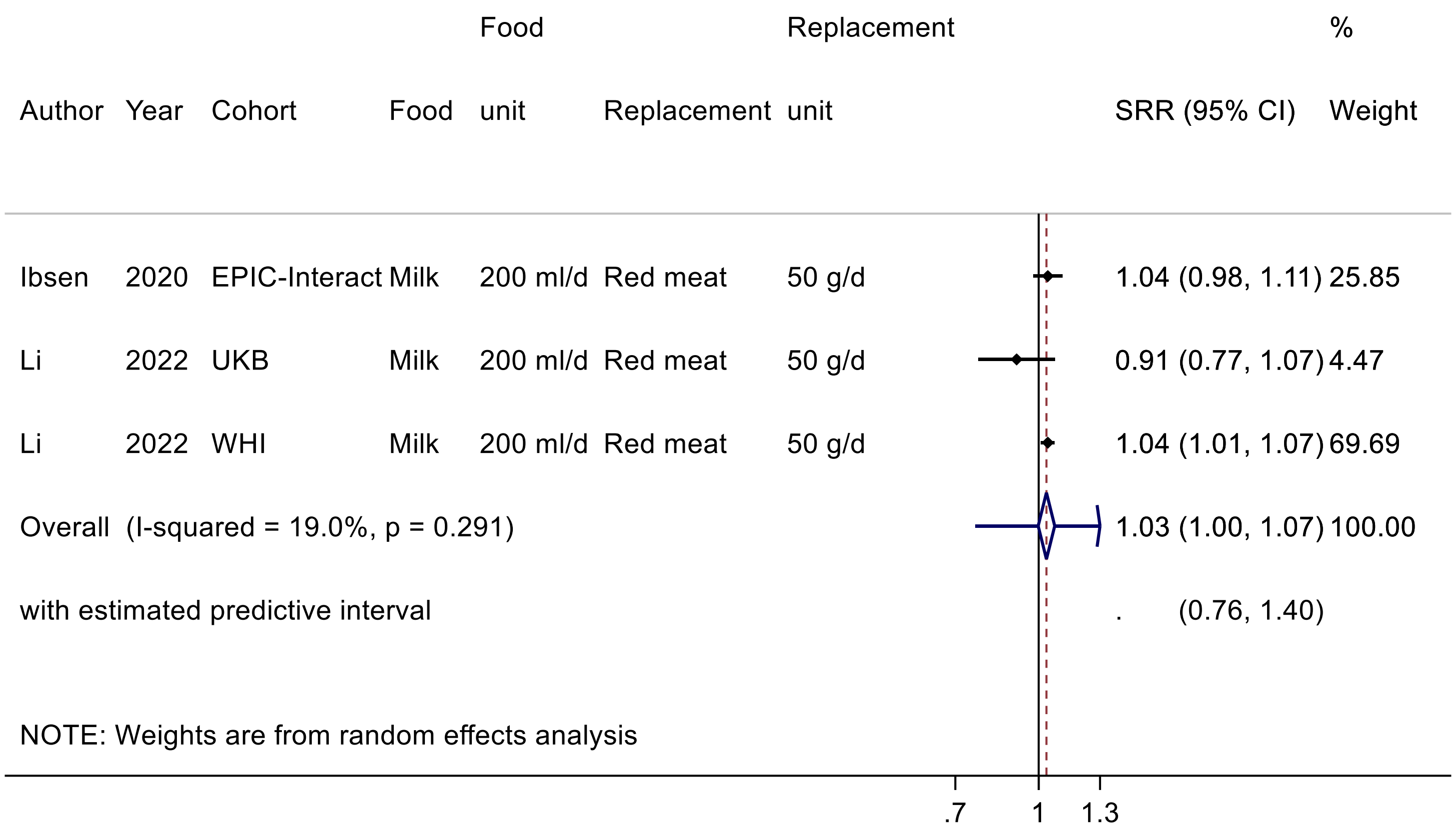

Supplementary figure 40: Meta-analysis comparing the substituting effect of substituting milk with red meat on type 2 diabetes risk
(replacement and substitution are used synonymously)

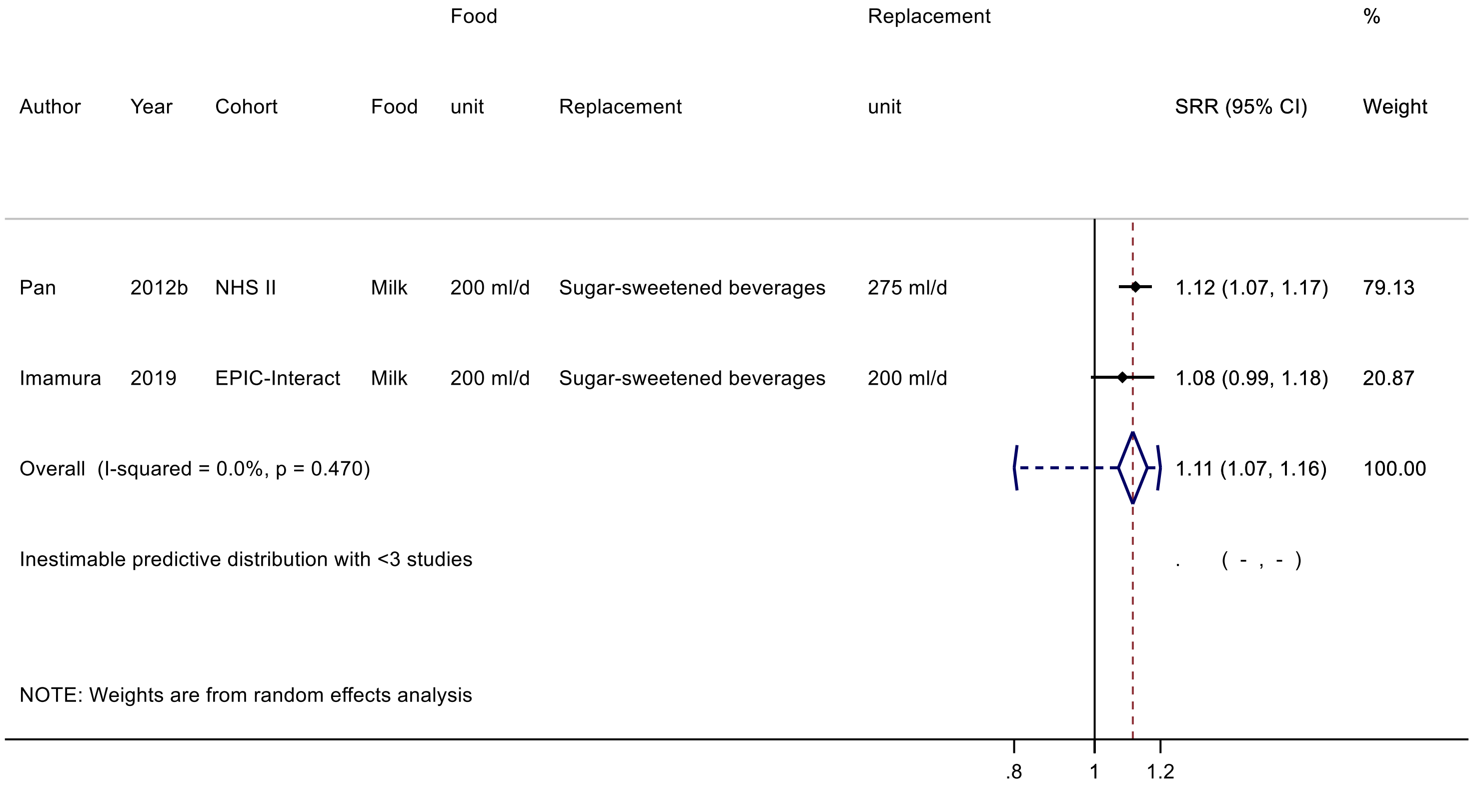

Supplementary figure 41: Meta-analysis comparing the substituting effect of substituting milk with sugar sweetened beverages on type 2 diabetes risk (replacement and substitution are used synonymously)

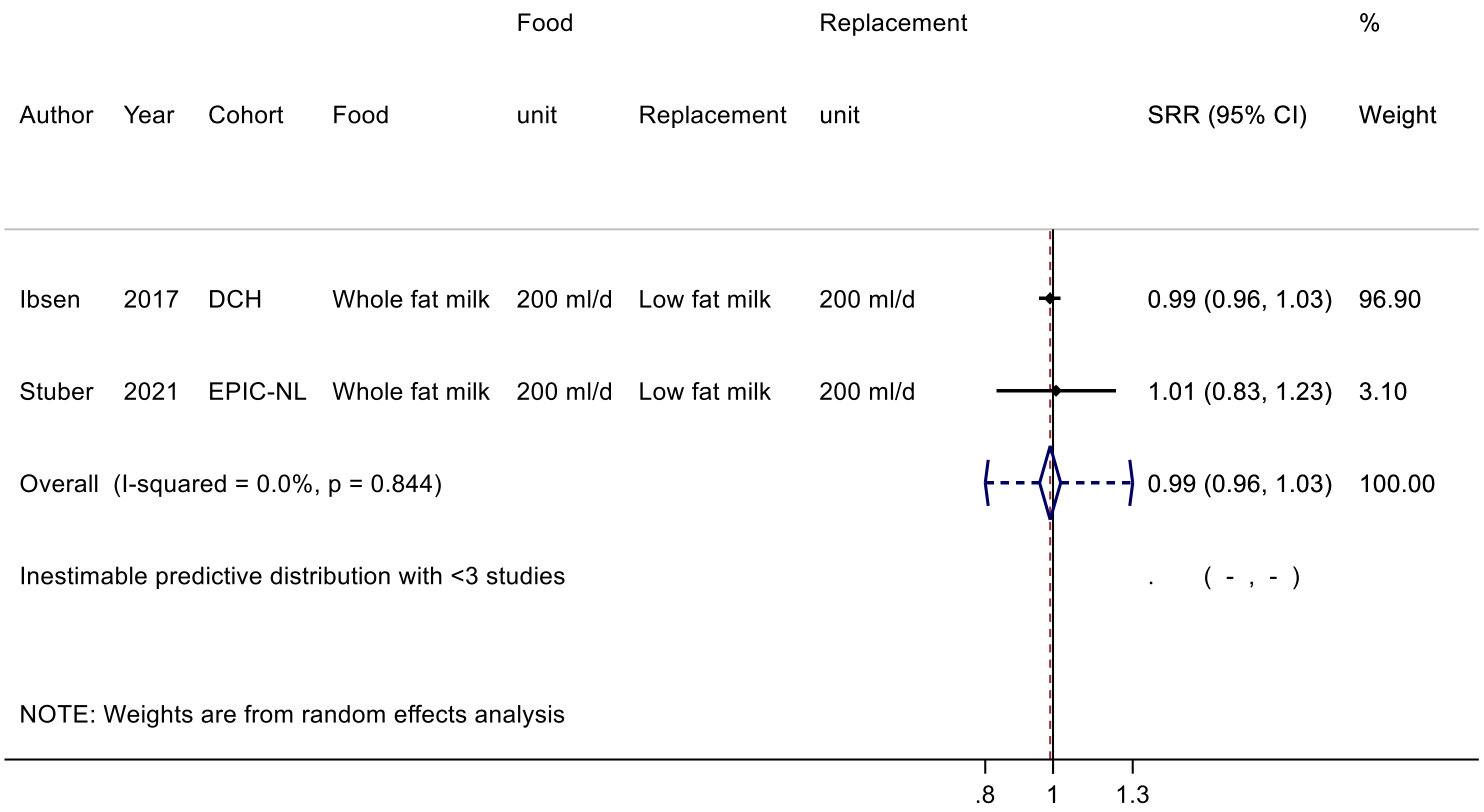

Supplementary figure 42: Meta-analysis comparing the substituting effect of substituting whole-fat milk with an equal amount of low-fat milk on type 2 diabetes risk (replacement and substitution are used synonymously)

Author	Year	Cohort	Food	Food unit	Replacement	Replacement unit	SRR (95% CI)	% Weight
Ibsen	2017	DCH	Whole fat milk	200 ml/d	Whole fat yogurt	200 g/d	0.89 (0.82, 0.96)	85.00
Stuber	2021	EPIC-NL	Whole fat milk	200 ml/d	Whole fat yogurt	200 g/d	1.17 (0.74, 1.84)	15.00
Overall (I-squared = 25.7%, p = 0.246)							0.93 (0.77, 1.12)	100.00
Inestimable predictive distribution with <3 studies							. (- , -)	

NOTE: Weights are from random effects analysis

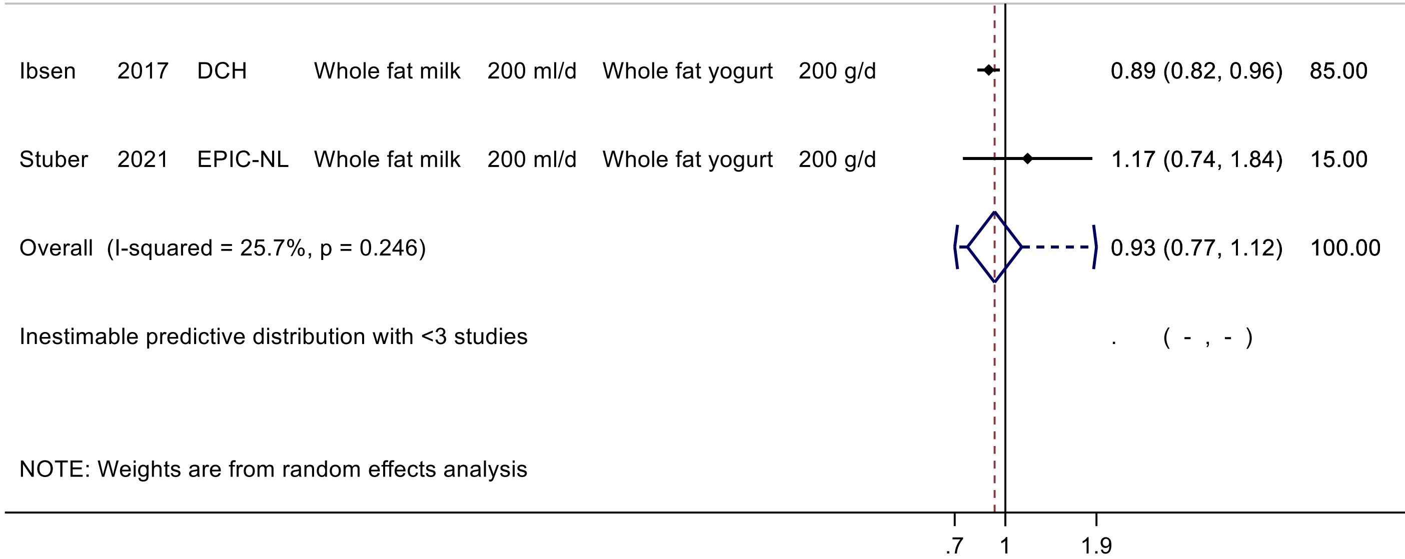

Supplementary figure 43: Meta-analysis comparing the substituting effect of substituting whole-fat milk with an equal amount of whole-fat yogurt on type 2 diabetes risk (replacement and substitution are used synonymously)

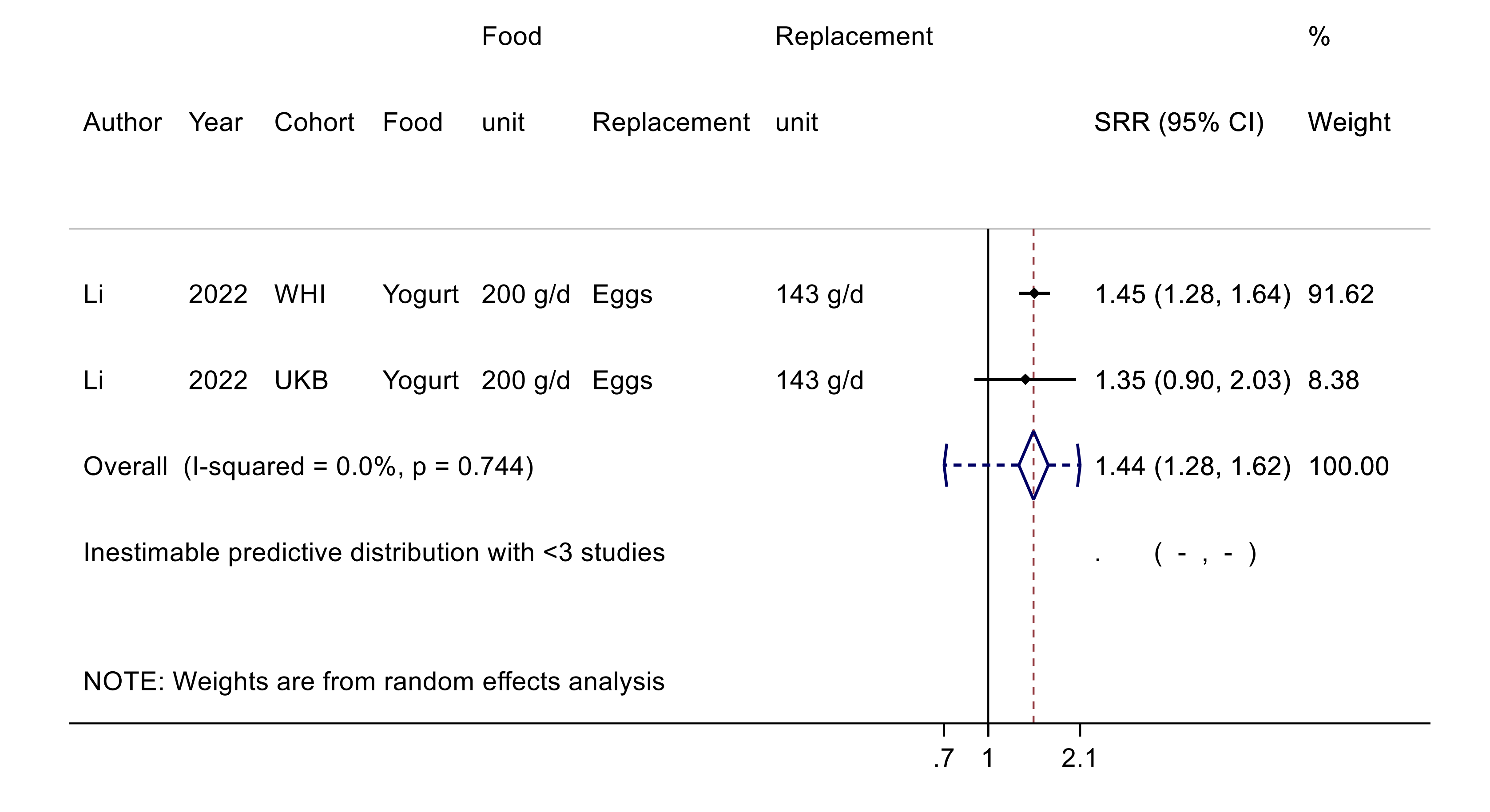

Supplementary figure 44: Meta-analysis comparing the substituting effect of substituting yogurt with eggs on type 2 diabetes risk (replacement and substitution are used synonymously)

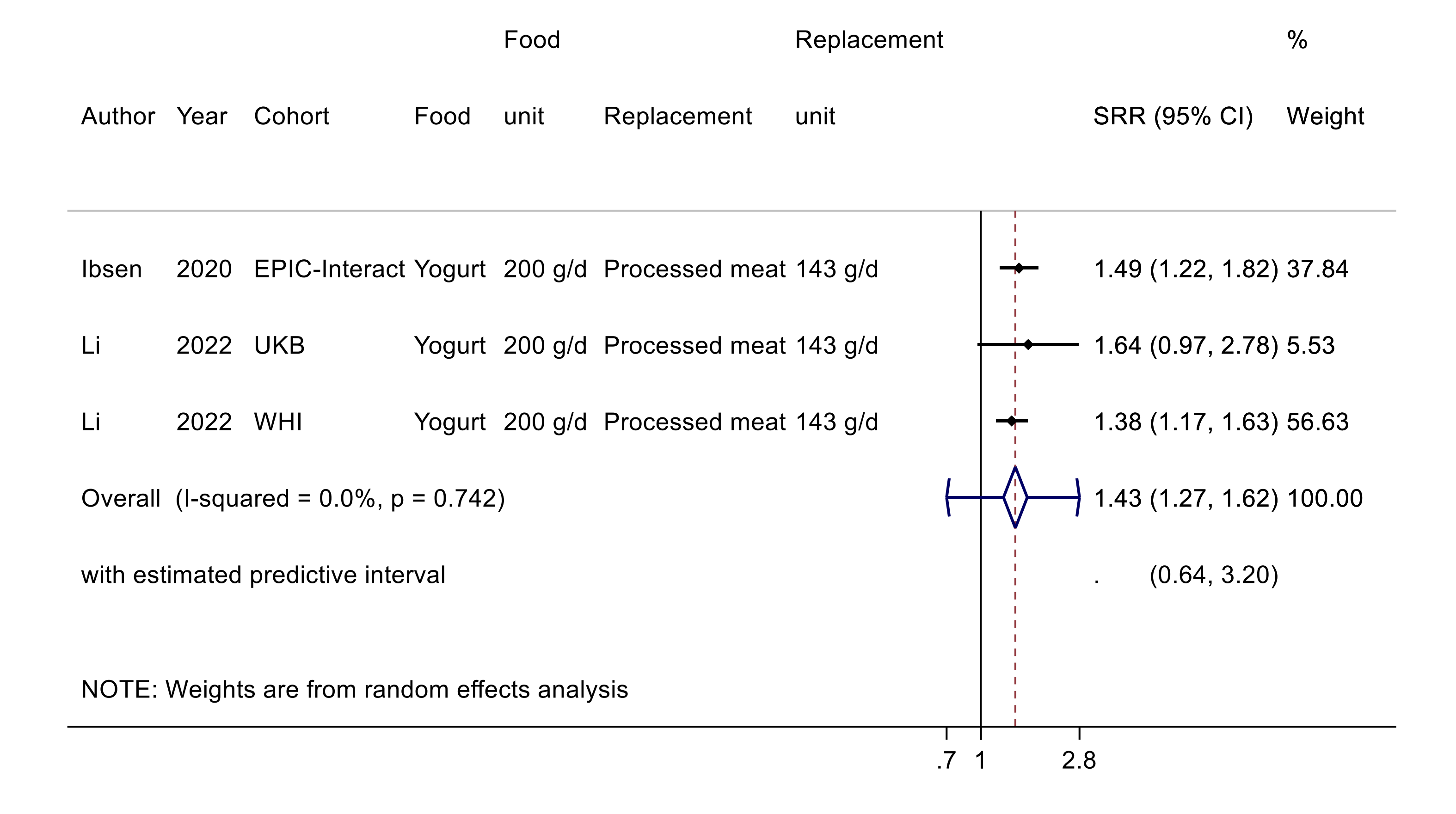

Supplementary figure 45: Meta-analysis comparing the substituting effect of substituting yogurt with processed meat on type 2 diabetes risk (replacement and substitution are used synonymously)

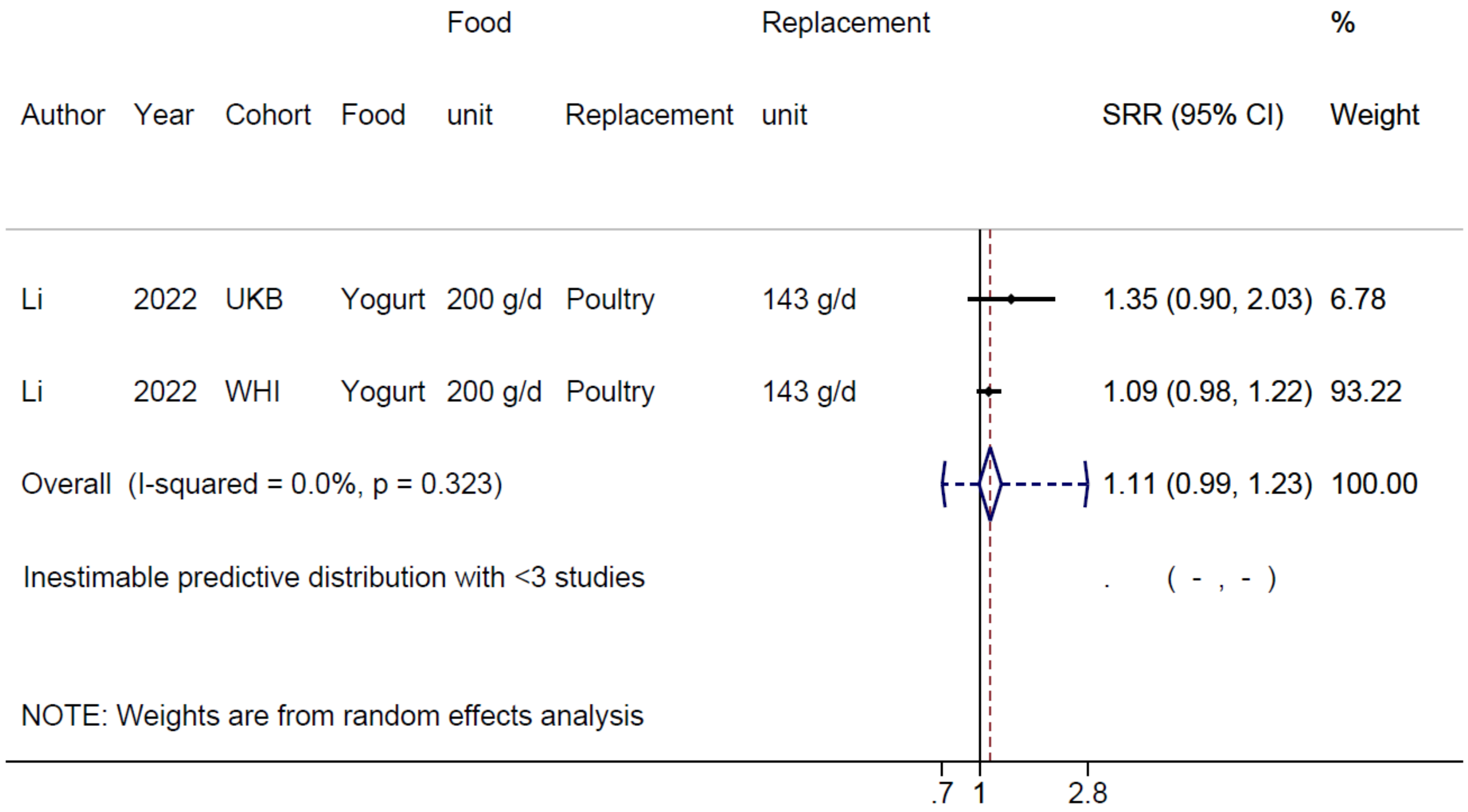

Supplementary figure 46: Meta-analysis comparing the substituting effect of substituting yogurt with poultry on type 2 diabetes risk (replacement and substitution are used synonymously)

Author	Year	Cohort	Food	Food unit	Replacement	Replacement unit	SRR (95% CI)	% Weight
Ibsen	2020	EPIC-Interact	Yogurt	200 g/d	Red meat	143 g/d	1.28 (1.04, 1.58)	28.29
Li	2022	UKB	Yogurt	200 g/d	Red meat	143 g/d	1.49 (1.07, 2.08)	14.95
Li	2022	WHI	Yogurt	200 g/d	Red meat	143 g/d	1.12 (1.03, 1.21)	56.76
Overall (I-squared = 45.9%, p = 0.157)							1.21 (1.05, 1.41)	100.00
with estimated predictive interval							. (0.27, 5.37)	

NOTE: Weights are from random effects analysis

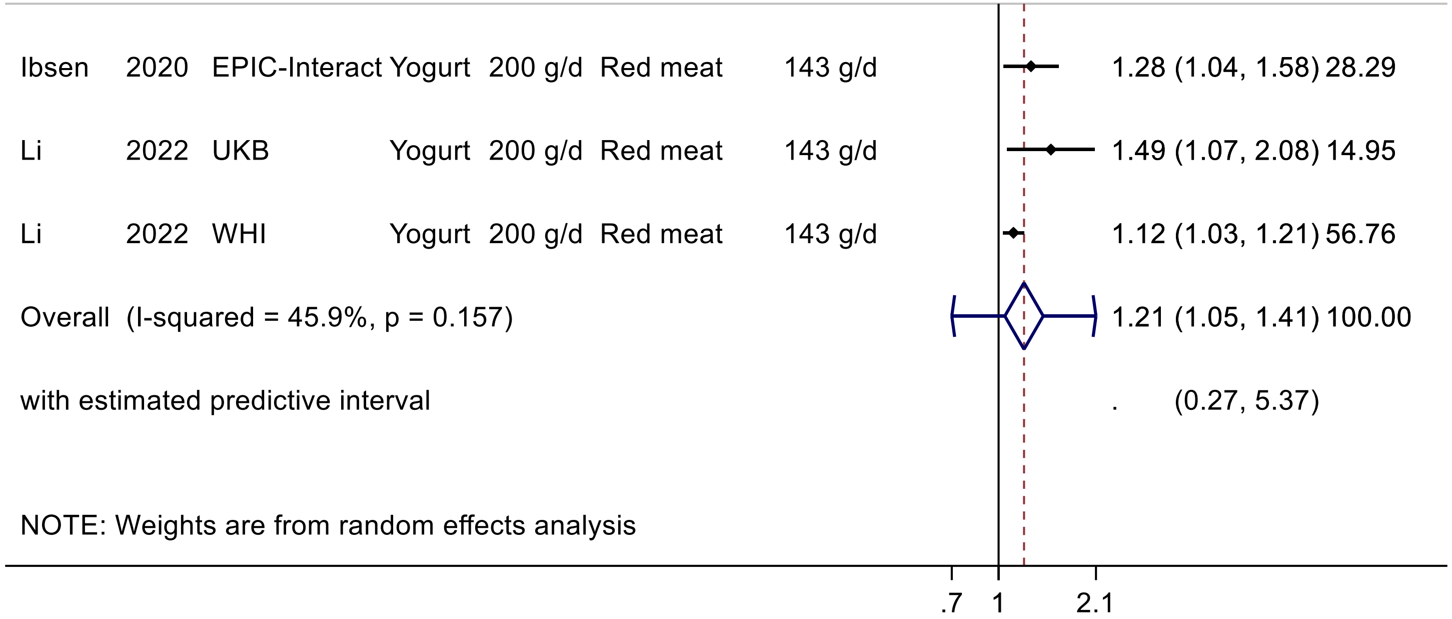

Supplementary figure 47: Meta-analysis comparing the substituting effect of substituting yogurt with red meat on type 2 diabetes risk
(replacement and substitution are used synonymously)

Supplementary References

1. Wu Y, Willett WC, Smith-Warner SA. Total red meat, unprocessed red meat, processed meat and risk of breast cancer-a pooled analysis of 23 cohort studies. Cancer Research. 2020;80(16_Supplement):3471-.

2. Yuzbashian E, Asghari G, Mirmiran P, Chan CB, Azizi F. Changes in dairy product consumption and subsequent type 2 diabetes among individuals with prediabetes: Tehran Lipid and Glucose Study. Nutrition Journal. 2021;20(1):88.

3. Ardisson Korat AV, Li Y, Sacks F, Rosner B, Willett WC, Hu FB, Sun Q. Dairy fat intake and risk of type 2 diabetes in 3 cohorts of US men and women. The American Journal of Clinical Nutrition. 2019;110(5):1192-200.

4. van den Brandt PA, Nieuwenhuis L. Tree nut, peanut, and peanut butter intake and risk of postmenopausal breast cancer: The Netherlands Cohort Study. Cancer Causes & Control. 2018;29(1):63-75.

5. Yang W, Sui J, Ma Y, Simon TG, Petrick JL, Lai M, et al. High Dietary Intake of Vegetable or Polyunsaturated Fats Is Associated With Reduced Risk of Hepatocellular Carcinoma. Clinical Gastroenterology and Hepatology. 2020;18(12):2775-83.e11.

6. Orlich MJ, Sabaté J, Mashchak A, Fresán U, Jaceldo-Siegl K, Miles F, Fraser GE. Ultra-processed food intake and animal-based food intake and mortality in the Adventist Health Study-2. The American Journal of Clinical Nutrition. 2022;115(6):1589-601.

7. Chen M, Li Y, Sun Q, Pan A, Manson JE, Rexrode KM, et al. Dairy fat and risk of cardiovascular disease in 3 cohorts of US adults1, 2, 3. The American Journal of Clinical Nutrition. 2016;104(5):1209-17.

8. de Oliveira Otto MC, Mozaffarian D, Kromhout D, Bertoni AG, Sibley CT, Jacobs DR, Nettleton JA. Dietary intake of saturated fat by food source and incident cardiovascular disease: the Multi-Ethnic Study of Atherosclerosis. The American Journal of Clinical Nutrition. 2012;96(2):397-404.

9. Vissers LET, Rijksen J, Boer JMA, Verschuren WMM, van der Schouw YT, Sluijs I. Fatty acids from dairy and meat and their association with risk of coronary heart disease. European Journal of Nutrition. 2019;58(7):2639-47.

10. Steur M, Johnson L, Sharp SJ, Imamura F, Sluijs I, Key TJ, et al. Dietary Fatty Acids, Macronutrient Substitutions, Food Sources and Incidence of Coronary Heart Disease: Findings From the EPIC-CVD Case-Cohort Study Across Nine European Countries. Journal of the American Heart Association. 2021;10(23):e019814.

11. Kant AK, Schatzkin A, Graubard BI, Schairer C. A Prospective Study of Diet Quality and Mortality in Women. JAMA. 2000;283(16):2109-15.

12. Seidelmann SB, Claggett B, Cheng S, Henglin M, Shah A, Steffen LM, et al. Dietary carbohydrate intake and mortality: a prospective cohort study and meta-analysis. The Lancet Public Health. 2018;3(9):e419-e28.

13. Haugsgjerd TR, Egeland GM, Nygård OK, Igland J, Sulo G, Lysne V, et al. Intake of carbohydrates and SFA and risk of CHD in middle-age adults: the Hordaland Health Study (HUSK). Public Health Nutrition. 2022;25(3):634-48.

14. Kelemen LE, Kushi LH, Jacobs DR, Jr., Cerhan JR. Associations of Dietary Protein with Disease and Mortality in a Prospective Study of Postmenopausal Women. American Journal of Epidemiology. 2005;161(3):239-49.

15. Liu S, van der Schouw YT, Soedamah-Muthu SS, Spijkerman AMW, Sluijs I. Intake of dietary saturated fatty acids and risk of type 2 diabetes in the European Prospective Investigation into Cancer and Nutrition-Netherlands cohort: associations by types, sources of fatty acids and substitution by macronutrients. European Journal of Nutrition. 2019;58(3):1125-36.

16. Jiang R, Manson JE, Stampfer MJ, Liu S, Willett WC, Hu FB. Nut and Peanut Butter Consumption and Risk of Type 2 Diabetes in Women. JAMA. 2002;288(20):2554-60.

17. Similä ME, Kontto JP, Valsta LM, Männistö S, Albanes D, Virtamo J. Carbohydrate substitution for fat or protein and risk of type 2 diabetes in male smokers. European Journal of Clinical Nutrition. 2012;66(6):716-21.

18. Praagman J, Beulens JWJ, Alssema M, Zock PL, Wanders AJ, Sluijs I, van der Schouw YT. The association between dietary saturated fatty acids and ischemic heart disease depends on the type and source of fatty acid in the European Prospective Investigation into Cancer and Nutrition–Netherlands cohort1,2. The American Journal of Clinical Nutrition. 2016;103(2):356-65.

19. Otto M, Jacobs DR, Mozaffarian D, Kromhout D, Bertoni AG, Sibley CT, Nettleton JA. Abstract MP004: Dietary Intakes of Saturated Fat and Cardiovascular Disease: Results from the Multi-Ethnic Study of Atherosclerosis. Circulation. 2012;125(suppl_10):AMP004-AMP.

20. O'Connor L, Imamura F, Lentjes MA, Khaw KT, Wareham NJ, Forouhi NG. Prospective associations and population impact of sweet beverage intake and type 2 diabetes, and effects of substitutions with alternative beverages. Diabetologia. 2015;58(7):1474-83.

21. Patterson E, Larsson SC, Wolk A, Åkesson A. Association between dairy food consumption and risk of myocardial infarction in women differs by type of dairy food. J Nutr. 2013;143(1):74-9.

22. Díaz-López A, Bulló M, Martínez-González MA, Corella D, Estruch R, Fitó M, et al. Dairy product consumption and risk of type 2 diabetes in an elderly Spanish Mediterranean population at high cardiovascular risk. European Journal of Nutrition. 2016;55(1):349-60.

23. Ibsen DB, Levitan EB, Åkesson A, Gigante B, Wolk A. The DASH diet is associated with a lower risk of heart failure: a cohort study. European Journal of Preventive Cardiology. 2022;29(7):1114-23.

24. Liang J, Zhao J-K, Wang J-P, Wang T. Association between animal source foods consumption and risk of hypertension: a cohort study. European Journal of Nutrition. 2021;60(5):2469-83.

25. Fraser GE, Jaceldo-Siegl K, Orlich M, Mashchak A, Sirirat R, Knutsen S. Dairy, soy, and risk of breast cancer: those confounded milks. Int J Epidemiol. 2020;49(5):1526-37.

26. Bostick RM, Potter JD, Kushi LH, Sellers TA, Steinmetz KA, McKenzie DR, et al. Sugar, meat, and fat intake, and non-dietary risk factors for colon cancer incidence in Iowa women (United States). Cancer Causes & Control. 1994;5(1):38-52.

27. Xu X, Kabir A, Barr ML, Schutte AE. Different Types of Long-Term Milk Consumption and Mortality in Adults with Cardiovascular Disease: A Population-Based Study in 7236 Australian Adults over 8.4 Years. Nutrients. 2022;14(3):704.

28. Ericson U, Hellstrand S, Brunkwall L, Schulz C-A, Sonestedt E, Wallström P, et al. Food sources of fat may clarify the inconsistent role of dietary fat intake for incidence of type 2 diabetes2, 3. The American Journal of Clinical Nutrition. 2015;101(5):1065-80.

29. Stasinopoulos LC, Zhou A, Hyppönen E. Association of supplemental calcium and dairy milk intake with all-cause and cause-specific mortality in the UK Biobank: a prospective cohort study. British Journal of Nutrition. 2020;123(5):574-82.

30. Avalos EE, Barrett-Connor E, Kritz-Silverstein D, Wingard DL, Bergstrom JN, Al-Delaimy WK. Is dairy product consumption associated with the incidence of CHD? Public Health Nutrition. 2013;16(11):2055-63.

31. Aguilera-Buenosvinos I, Fernandez-Lazaro CI, Romanos-Nanclares A, Gea A, Sánchez-Bayona R, Martín-Moreno JM, et al. Dairy Consumption and Incidence of Breast Cancer in the 'Seguimiento Universidad de Navarra' (SUN) Project. Nutrients. 2021;13(2):687.

32. Ibsen DB, Jakobsen MU, Halkjær J, Tjønneland A, Kilpeläinen TO, Parner ET, Overvad K. Replacing Red Meat with Other Nonmeat Food Sources of Protein is Associated with a Reduced Risk of Type 2 Diabetes in a Danish Cohort of Middle-Aged Adults. J Nutr. 2021b;151(5):1241-8.

33. Ibsen DB, Overvad K, Laursen ASD, Halkjær J, Tjønneland A, Kilpeläinen TO, et al. Changes in intake of dairy product subgroups and risk of type 2 diabetes: modelling specified food substitutions in the Danish Diet, Cancer and Health cohort. Eur J Nutr. 2021a;60(6):3449-59.

34. Drouin-Chartier JP, Li Y, Ardisson Korat AV, Ding M, Lamarche B, Manson JE, et al. Changes in dairy product consumption and risk of type 2 diabetes: results from 3 large prospective cohorts of US men and women. Am J Clin Nutr. 2019;110(5):1201-12.

35. Zheng Y, Li Y, Satija A, Pan A, Sotos-Prieto M, Rimm E, et al. Association of changes in red meat consumption with total and cause specific mortality among US women and men: two prospective cohort studies. BMJ. 2019;365:l2110.

36. Würtz AML, Jakobsen MU, Bertoia ML, Hou T, Schmidt EB, Willett WC, et al. Replacing the consumption of red meat with other major dietary protein sources and risk of type 2 diabetes mellitus: a prospective cohort study. Am J Clin Nutr. 2021;113(3):612-21.

37. Lim CG, Dam RMv. Abstract 10985: Replacing Dietary Carbohydrates and Refined Grains with Different Alternatives and Risk of Cardiovascular Diseases in a Multi-Ethnic Asian Population. Circulation. 2021;144(Suppl_1):A10985-A.

38. Poster Session 2. European Journal of Preventive Cardiology. 2020;26(1_suppl):S71-S127.

39. de Goede J, Soedamah-Muthu SS, Trichia E, Geleijnse JM, Kromhout D. Abstract P061: Dietary Intake of Saturated Fat by Food Source and Incident Coronary Heart Disease: the Zutphen Elderly Study. Circulation. 2015;131(suppl_1):AP061-AP.

40. DROUIN-CHARTIER J-P, LI Y, ARDISSON KORAT AV, DING M, LAMARCHE B, MANSON JE, et al. 159-OR: Changes in Dairy Product Consumption and Risk of Type 2 Diabetes among U.S. Men and Women. Diabetes. 2019;68(Supplement_1).

41. Abstracts of the EASD, Stockholm 2010. Diabetologia. 2010;53(1):1-556.

42. Abstracts of the 50th EASD Annual Meeting. Diabetologia. 2014;57(1):1-564.

43. Al-Shaar L, Wang M, Willett W, Smith-Warner S. Abstract MP55: Red And Processed Meat And Alternative Protein Sources In Relation To Risk Of Fatal Coronary Heart Disease: A Pooled Analysis Of 16 Prospective Cohort Studies. Circulation. 2021;143(Suppl_1):AMP55-AMP.

44. Guasch-Ferre M, Liu G, Li Y, Manson JE, Salas-Salvadó J, Martínez-González MA. Olive Oil Consumption and Cardiovascular Risk in U.S Adults. Journal of Vascular Surgery. 2020;72(3):1144.

45. Bols E, Smits L, Weijenberg M. Healthy Living: The European Congress of Epidemiology, 2015. European Journal of Epidemiology. 2015;30(8):709-1001.

46. Korat AA, Hu FB, Sun Q. Abstract P234: Dairy Fat Intake and Risk of Type 2 Diabetes in 3 Cohorts of US Adults. Circulation. 2018;137(suppl_1):AP234-AP.

47. Hur J, Otegbeye E, Joh H-K, Nimptsch K, Ng K, Ogino S, et al. Abstract 838: Sugar-sweetened beverage intake and risk of early-onset colorectal cancer. Cancer Research. 2021;81(13_Supplement):838-.

48. Chen M, Sun Q, Pan A, Li Y, Manson JE, Rexrode KM, et al. Abstract P282: Dairy Fat and Risk of Cardiovascular Disease in 3 Cohorts of US Adults. Circulation. 2016;133(suppl_1):AP282-AP.

49. Guasch M, Liu G, Li Y, Sampson L, Manson JE, Salas-Salvado J, et al. Abstract P509: Olive Oil Consumption and Risk of Cardiovascular Disease. Circulation. 2020;141(Suppl_1):AP509-AP.

50. Praagman J, Jonge EALd, Jong JCK-d, Beulens JWJ, Sluijs I, Schoufour JD, et al. Dietary Saturated Fatty Acids and Coronary Heart Disease Risk in a Dutch Middle-Aged and Elderly Population. Arteriosclerosis, Thrombosis, and Vascular Biology. 2016;36(9):2011-8.

51. Steur M. Abstract 50: Associations of Dietary Saturated Fatty Acids With Incident Coronary Heart Disease Depend on the Food Sources: EPIC-CVD Prospective Case-cohort Study in Nine European Countries. Circulation. 2020;141(Suppl_1):A50-A.

52. Pan A, Sun Q, Willett WC, Hu FB. Abstract 16109: Red Meat Consumption and Mortality: Results From Two Cohorts of American Adults. Circulation. 2011;124(suppl_21):A16109-A.

53. Feldman AL, Long GH, Johansson I, Weinehall L, Fhärm E, Wennberg P, et al. Change in lifestyle behaviors and diabetes risk: evidence from a population-based cohort study with 10 year follow-up. Int J Behav Nutr Phys Act. 2017;14(1):39.

54. VanWormer JJ, Boucher JL, Sidebottom AC, Sillah A, Knickelbine T. Lifestyle changes and prevention of metabolic syndrome in the Heart of New Ulm Project. Preventive Medicine Reports. 2017;6:242-5.

55. Al-Shaar L, Satija A, Wang DD, Rimm EB, Smith-Warner SA, Stampfer MJ, et al. Red meat intake and risk of coronary heart disease among US men: prospective cohort study. BMJ. 2020;371:m4141.

56. Bernstein AM, Sun Q, Hu FB, Stampfer MJ, Manson JE, Willett WC. Major dietary protein sources and risk of coronary heart disease in women. Circulation. 2010;122(9):876-83.

57. Bernstein AM, Pan A, Rexrode KM, Stampfer M, Hu FB, Mozaffarian D, Willett WC. Dietary protein sources and the risk of stroke in men and women. Stroke. 2012;43(3):637-44.

58. Ding M, Li J, Qi L, Ellervik C, Zhang X, Manson JE, et al. Associations of dairy intake with risk of mortality in women and men: three prospective cohort studies. BMJ. 2019;367:l6204.

59. Guasch-Ferré M, Hruby A, Salas-Salvadó J, Martínez-González MA, Sun Q, Willett WC, Hu FB. Olive oil consumption and risk of type 2 diabetes in US women. Am J Clin Nutr. 2015;102(2):479-86.

60. Guasch-Ferré M, Liu G, Li Y, Sampson L, Manson JE, Salas-Salvadó J, et al. Olive Oil Consumption and Cardiovascular Risk in U.S. Adults. J Am Coll Cardiol. 2020;75(15):1729-39.

61. Guasch-Ferré M, Li Y, Willett WC, Sun Q, Sampson L, Salas-Salvadó J, et al. Consumption of Olive Oil and Risk of Total and Cause-Specific Mortality Among U.S. Adults. J Am Coll Cardiol. 2022;79(2):101-12.

62. Haring B, Gronroos N, Nettleton JA, von Ballmoos MC, Selvin E, Alonso A. Dietary protein intake and coronary heart disease in a large community based cohort: results from the Atherosclerosis Risk in Communities (ARIC) study [corrected]. PLoS One. 2014;9(10):e109552.

63. Ibsen DB, Laursen ASD, Lauritzen L, Tjønneland A, Overvad K, Jakobsen MU. Substitutions between dairy product subgroups and risk of type 2 diabetes: the Danish Diet, Cancer and Health cohort. Br J Nutr. 2017;118(11):989-97.

64. Ibsen DB, Steur M, Imamura F, Overvad K, Schulze MB, Bendinelli B, et al. Replacement of Red and Processed Meat With Other Food Sources of Protein and the Risk of Type 2 Diabetes in European Populations: The EPIC-InterAct Study. Diabetes Care. 2020;43(11):2660-7.

65. Imamura F, Schulze MB, Sharp SJ, Guevara M, Romaguera D, Bendinelli B, et al. Estimated Substitution of Tea or Coffee for Sugar-Sweetened Beverages Was Associated with Lower Type 2 Diabetes Incidence in Case-Cohort Analysis across 8 European Countries in the EPIC-InterAct Study. J Nutr. 2019;149(11):1985-93.

66. Keller A, O'Reilly EJ, Malik V, Buring JE, Andersen I, Steffen L, et al. Substitution of sugar-sweetened beverages for other beverages and the risk of developing coronary heart disease: Results from the Harvard Pooling Project of Diet and Coronary Disease. Prev Med. 2020;131:105970.

67. Key TJ, Appleby PN, Bradbury KE, Sweeting M, Wood A, Johansson I, et al. Consumption of Meat, Fish, Dairy Products, and Eggs and Risk of Ischemic Heart Disease. Circulation. 2019;139(25):2835-45.

68. Kvist K, Laursen ASD, Overvad K, Jakobsen MU. Substitution of Milk with Whole-Fat Yogurt Products or Cheese Is Associated with a Lower Risk of Myocardial Infarction: The Danish Diet, Cancer and Health cohort. J Nutr. 2020;150(5):1252-8.

69. Laursen ASD, Dahm CC, Johnsen SP, Tjønneland A, Overvad K, Jakobsen MU. Substitutions of dairy product intake and risk of stroke: a Danish cohort study. Eur J Epidemiol. 2018;33(2):201-12.

70. Laursen ASD, Sluijs I, Boer JMA, Verschuren WMM, van der Schouw YT, Jakobsen MU. Substitutions between dairy products and risk of stroke: results from the European Investigation into Cancer and Nutrition-Netherlands (EPIC-NL) cohort. Br J Nutr. 2019;121(12):1398-404.

71. Laursen ASD, Thomsen AL, Beck A, Overvad K, Jakobsen MU. Theoretical substitutions between dairy products and all-cause and cause-specific mortality. Results from the Danish diet, cancer and health cohort. Br J Nutr. 2022;127(10):1557-66.

72. Li J, Glenn AJ, Yang Q, Ding D, Zheng L, Bao W, et al. Dietary Protein Sources, Mediating Biomarkers, and Incidence of Type 2 Diabetes: Findings From the Women's Health Initiative and the UK Biobank. Diabetes Care. 2022;45(8):1742-53.

73. Lim CGY, Tai ES, van Dam RM. Replacing dietary carbohydrates and refined grains with different alternatives and risk of cardiovascular diseases in a multi-ethnic Asian population. Am J Clin Nutr. 2022;115(3):854-63.

74. Liu Q, Rossouw JE, Roberts MB, Liu S, Johnson KC, Shikany JM, et al. Theoretical Effects of Substituting Butter with Margarine on Risk of Cardiovascular Disease. Epidemiology. 2017;28(1):145-56.

75. Lyskjær L, Overvad K, Tjønneland A, Dahm CC. Substitutions of Oatmeal and Breakfast Food Alternatives and the Rate of Stroke. Stroke. 2020;51(1):75-81.

76. Malik VS, Li Y, Tobias DK, Pan A, Hu FB. Dietary Protein Intake and Risk of Type 2 Diabetes in US Men and Women. Am J Epidemiol. 2016;183(8):715-28.

77. Pacheco LS, Li Y, Rimm EB, Manson JE, Sun Q, Rexrode K, et al. Avocado Consumption and Risk of Cardiovascular Disease in US Adults. J Am Heart Assoc. 2022;11(7):e024014.

78. Pan A, Sun Q, Bernstein AM, Schulze MB, Manson JE, Willett WC, Hu FB. Red meat consumption and risk of type 2 diabetes: 3 cohorts of US adults and an updated meta-analysis. Am J Clin Nutr. 2011;94(4):1088-96.

79. Pan A, Sun Q, Bernstein AM, Schulze MB, Manson JE, Stampfer MJ, et al. Red meat consumption and mortality: results from 2 prospective cohort studies. Arch Intern Med. 2012a;172(7):555-63.

80. Pan A, Malik VS, Schulze MB, Manson JE, Willett WC, Hu FB. Plain-water intake and risk of type 2 diabetes in young and middle-aged women. Am J Clin Nutr. 2012b;95(6):1454-60.

81. Schmid D, Song M, Zhang X, Willett WC, Vaidya R, Giovannucci EL, Michels KB. Yogurt consumption in relation to mortality from cardiovascular disease, cancer, and all causes: a prospective investigation in 2 cohorts of US women and men. Am J Clin Nutr. 2020;111(3):689-97.

82. Stuber JM, Vissers LET, Verschuren WMM, Boer JMA, van der Schouw YT, Sluijs I. Substitution among milk and yogurt products and the risk of incident type 2 diabetes in the EPIC-NL cohort. J Hum Nutr Diet. 2021;34(1):54-63.

83. Sun Y, Liu B, Snetselaar LG, Wallace RB, Shadyab AH, Kroenke CH, et al. Association of Major Dietary Protein Sources With All-Cause and Cause-Specific Mortality: Prospective Cohort Study. J Am Heart Assoc. 2021;10(5):e015553.

84. van den Brandt PA. Red meat, processed meat, and other dietary protein sources and risk of overall and cause-specific mortality in The Netherlands Cohort Study. Eur J Epidemiol. 2019;34(4):351-69.

85. Virtanen HEK, Koskinen TT, Voutilainen S, Mursu J, Tuomainen TP, Kokko P, Virtanen JK. Intake of different dietary proteins and risk of type 2 diabetes in men: the Kuopio Ischaemic Heart Disease Risk Factor Study. Br J Nutr. 2017;117(6):882-93.

86. Zhang Y, Zhuang P, Wu F, He W, Mao L, Jia W, et al. Cooking oil/fat consumption and deaths from cardiometabolic diseases and other causes: prospective analysis of 521,120 individuals. BMC Med. 2021;19(1):92.

87. Zhuang P, Jiao J, Wu F, Mao L, Zhang Y. Egg and egg-sourced cholesterol consumption in relation to mortality: Findings from population-based nationwide cohort. Clin Nutr. 2020;39(11):3520-7.

88. Zhuang P, Wu F, Mao L, Zhu F, Zhang Y, Chen X, et al. Egg and cholesterol consumption and mortality from cardiovascular and different causes in the United States: A population-based cohort study. PLoS Med. 2021;18(2):e1003508.
